# Psychological distress, depression, anxiety, and life satisfaction following COVID-19 infection: evidence from 11 UK longitudinal population studies

**DOI:** 10.1016/S2215-0366(22)00307-8

**Published:** 2022-11

**Authors:** Ellen J Thompson, Jean Stafford, Bettina Moltrecht, Charlotte F Huggins, Alex S F Kwong, Richard J Shaw, Paola Zaninotto, Kishan Patel, Richard J Silverwood, Eoin McElroy, Matthias Pierce, Michael J Green, Ruth C E Bowyer, Jane Maddock, Kate Tilling, S Vittal Katikireddi, George B Ploubidis, David J Porteous, Nic Timpson, Nish Chaturvedi, Claire J Steves, Praveetha Patalay

**Affiliations:** a Department of Twin Research and Genetic Epidemiology, School of Life Course and Population Sciences, King's College London, London, UK; b MRC Unit for Lifelong Health and Ageing, University College London, London, UK; c Centre for Longitudinal Studies, University College London, London, UK; d Department of Epidemiology and Public Health, University College London, London, UK; e Centre for Genomic and Experimental Medicine, University of Edinburgh, Edinburgh, UK; f Division of Psychiatry, University of Edinburgh, Edinburgh, UK; g MRC/CSO Social & Public Health Sciences Unit, School of Health and Wellbeing, University of Glasgow, Glasgow, UK; h School of Psychology, Ulster University, Ulster, UK; i Division of Psychology and Mental Health, The University of Manchester, Manchester, UK; j Population Health Sciences, Bristol Medical School, University of Bristol, Bristol, UK

## Abstract

**Background:**

Evidence on associations between COVID-19 illness and mental health is mixed. We aimed to examine whether COVID-19 is associated with deterioration in mental health while considering pre-pandemic mental health, time since infection, subgroup differences, and confirmation of infection via self-reported test and serology data.

**Methods:**

We obtained data from 11 UK longitudinal studies with repeated measures of mental health (psychological distress, depression, anxiety, and life satisfaction; mental health scales were standardised within each study across time) and COVID-19 status between April, 2020, and April, 2021. We included participants with information available on at least one mental health outcome measure and self-reported COVID-19 status (suspected or test-confirmed) during the pandemic, and a subset with serology-confirmed COVID-19. Furthermore, only participants who had available data on a minimum set of covariates, including age, sex, and pre-pandemic mental health were included. We investigated associations between having ever had COVID-19 and mental health outcomes using generalised estimating equations. We examined whether associations varied by age, sex, ethnicity, education, and pre-pandemic mental health, whether the strength of the association varied according to time since infection, and whether associations differed between self-reported versus confirmed (by test or serology) infection.

**Findings:**

Between 21 Dec, 2021, and July 11, 2022, we analysed data from 54 442 participants (ranging from a minimum age of 16 years in one study to a maximum category of 90 years and older in another; including 33 200 [61·0%] women and 21 242 [39·0%] men) from 11 longitudinal UK studies. Of 40 819 participants with available ethnicity data, 36 802 (90·2%) were White. Pooled estimates of standardised differences in outcomes suggested associations between COVID-19 and subsequent psychological distress (0·10 [95% CI 0·06 to 0·13], *I*^2^=42·8%), depression (0·08 [0·05 to 0·10], *I*^2^=20·8%), anxiety (0·08 [0·05 to 0·10], *I*^2^=0·0%), and lower life satisfaction (–0·06 [–0·08 to –0·04], *I*^2^=29·2%). We found no evidence of interactions between COVID-19 and sex, education, ethnicity, or pre-pandemic mental health. Associations did not vary substantially between time since infection of less than 4 weeks, 4–12 weeks, and more than 12 weeks, and were present in all age groups, with some evidence of stronger effects in those aged 50 years and older. Participants who self-reported COVID-19 but had negative serology had worse mental health outcomes for all measures than those without COVID-19 based on serology and self-report. Participants who had positive serology but did not self-report COVID-19 did not show association with mental health outcomes.

**Interpretation:**

Self-reporting COVID-19 was longitudinally associated with deterioration in mental health and life satisfaction. Our findings emphasise the need for greater post-infection mental health service provision, given the substantial prevalence of COVID-19 in the UK and worldwide.

**Funding:**

UK Medical Research Council and UK National Institute for Health and Care Research.

## Introduction

Infection with severe acute respiratory syndrome coronavirus 2 (SARS-CoV-2) can cause asymptomatic or symptomatic COVID-19. Mental ill-health is increasingly recognised as a potential consequence of COVID-19, following initial evidence from case reports and studies of other severe coronavirus infections.^1^ However, longitudinal evidence in this area is limited and few studies have sought to disentangle the effects of COVID-19 illness from the wider mental health impacts of the pandemic.^2^ As such, the mental health consequences of COVID-19 in the general population remain poorly understood.

Recent systematic reviews have yielded mixed results as to whether COVID-19 illness is associated with psychological distress,^3^, ^4^ which might reflect a lack of high-quality longitudinal evidence in this area. Previous studies have been limited by small or unrepresentative samples, cross-sectional designs, and absence of control groups.^5^, ^6^ Although several studies using routine data reported elevated rates of psychiatric disorders following COVID-19 illness,^7^, ^8^, ^9^, ^10^, ^11^, ^12^ others have not found clear evidence of associations^13^, ^14^ and have mainly focused on more severe COVID-19 and recorded mental health disorders.^15^, ^16^ A study using data from six cohorts in Europe found that severe acute COVID-19 illness was associated with adverse mental health outcomes.^17^ Additionally, longitudinal studies in the UK have found associations between COVID-19 and psychological distress,^18^, ^19^, ^20^ although findings have been mixed across different mental health outcomes^21^ and, in the COVID Symptom Study, a modest association was found only in older participants.^20^ Further longitudinal research is needed to clarify previous mixed findings, to investigate the magnitude of any association, and to examine whether associations are sustained in the longer-term after infection.

**Research in context**

**Evidence before this study**

Although previous research suggests that SARS CoV-2 infection might increase risk of psychological distress, findings have been mixed and longitudinal evidence is limited. We searched PubMed for studies published in English from Jan 1, 2020, until April 1, 2022, using terms pertaining to “COVID-19” (focusing specifically on “infection”) and “mental health” (including “psychological distress,” “depression”, and “anxiety”). Most studies were cross-sectional, lacked comparison groups, and focused on patients admitted to hospital because of COVID-19. Several longitudinal studies, including prospective cohorts and studies using electronic health records, found associations between COVID-19 and psychological distress, although findings were mixed, with some evidence of attenuating effects over time. Further longitudinal research is needed involving general population samples, including subclinical psychological distress and common mental disorders, and accounting for pre-pandemic confounders.

**Added value of this study**

Using data from 11 UK longitudinal studies including 54 442 participants with detailed information on pre-pandemic confounders, we found associations between COVID-19 and subsequent psychological distress, depression, anxiety, and lower life satisfaction. Associations were present in all age groups but were strongest for middle-aged and older people. Effects did not attenuate over time in the 12 weeks after infection and were found for self-reported suspected and test-confirmed COVID-19. Participants who self-reported suspected or test-confirmed COVID-19, including those with a negative serology result, showed deterioration in their mental health. However, this association was not found in participants with positive serology who did not self-report COVID-19. These findings raise the possibility that the effects observed are not specific to SARS-CoV-2 infection but could still reflect the experience of illness during this period, or be explained by other factors.

**Implications of all the available evidence**

The evidence considered together indicates that COVID-19, in both the general population and in patients admitted to hospital, is associated with deterioration in mental health at clinical and subclinical levels. These findings have important implications for post-COVID-19 mental health service provision, given the substantial global prevalence of COVID-19. Our findings also suggest that these associations might not be specific to SARS-CoV-2, with other infections with similar symptoms also potentially resulting in worse mental health, and it is possible that this effect was specific to or amplified by the wider pandemic setting. Our findings comparing self-report and serology status highlight the potential salience of psychosocial mechanisms, such as social isolation, loss of pay, worry about infecting others, and the unpredictable course of COVID-19, in underpinning some of the associations between self-reported COVID-19 and mental health.

Using data from 11 UK longitudinal studies, we aimed to investigate mental health consequences following COVID-19 illness up to April, 2021. First, we examined whether individuals with self-reported COVID-19 experience higher levels of subsequent psychological distress, depression, and anxiety, and lower life satisfaction than those without self-reported COVID-19. Second, we examined whether associations varied depending on how much time had passed since infection to determine whether effects persist beyond the acute phase of the illness. Third, we explored whether associations varied by age, sex, ethnicity, education, and pre-pandemic mental health. Fourth, we examined whether associations between COVID-19 and mental health differed between those with suspected versus test-confirmed COVID-19, and self-reported versus serology-detected COVID-19.

## Methods

### Study design and participants

The UK National Core Studies Longitudinal Health and Wellbeing programme combines data from multiple UK population-based longitudinal studies to support more robust inferences that are replicable across data sources. Coordinated analysis across different datasets minimises methodological heterogeneity and maximises comparability, while appropriately accounting for study designs and characteristics of individual datasets. The analyses were preregistered on the Open Science Framework (10.17605/OSF.IO/KF2GA).

Data were drawn from 11 longitudinal UK population studies that conducted surveys before and during the COVID-19 pandemic, with the latter surveys being done between April, 2020, and March, 2021**,** and serology testing until June, 2021. For surveys done during the COVID-19 pandemic, serology data indicating the presence or absence of a SARS-CoV-2 infection were available. Details of study designs, timing of the most recent pre-pandemic and COVID-19 surveys, response rates, region within the UK, and analytical sample sizes are shown in the table.

**Table:** 11 UK longitudinal studies

	**Design and sample frame**	**2020 age range, years**	**Pre-pandemic survey**	**Details of COVID-19 surveys (response rate)**	**Mental health measure**	**Serology data**	**Participants with data analysed, n**
**Age homogenous cohorts**							
Millennium Cohort Study	Cohort of UK children born between September, 2000, and January, 2002, with regular follow-up surveys from birth	18–20	2018	Three surveys: May 2020 (26·6%); September–October, 2020 (24·2%); February–March, 2021 (22%)	Kessler Psychological Distress Scale-6	987 valid samples obtained April–June, 2021	4652
Avon Longitudinal Study of Parents and Children-Generation 1	Cohort of children born in southwest England between April, 1991, and December, 1992, with regular follow-up surveys from birth (original young people)	27–29	2017–18	Three surveys: April, 2020 (19%); June, 2020 (17·4%); December, 2020 (26·4%)	Short Mood and Feelings Questionnaire	NA	2498
Next Steps (formerly known as Longitudinal Study of Young People in England)	Sample recruited via secondary schools in England at about age 13 years with regular follow-up surveys	29–31	2015	Three surveys: May, 2020 (20·3%); September–October, 2020 (31·8%); February–March, 2021 (29%)	General Health Questionnaire 12	1037 valid samples obtained April–June, 2021	4092
British Cohort Study 1970	Cohort of all children born in Great Britain (ie, England, Wales, and Scotland) in 1 week in 1970, with regular follow-up surveys from birth.	50	2016	Three surveys: May, 2020 (40·4%); September–October, 2020 (43·9%); February–March, 2021 (40%)	9-item Malaise Inventory	2074 valid samples obtained April −June, 2021	5545
National Child Development Study	Cohort of all children born in Great Britain (ie, England, Wales, and Scotland) in 1 week in 1958, with regular follow-up surveys from birth	62	2013	Three surveys: May, 2020 (57·9%); September–October, 2020 (53·9%); February–March, 2021 (52%)	9-item Malaise Inventory	2722 valid samples obtained April–June, 2021	6696
National Survey of Health and Development	Cohort of all children born in Great Britain (ie, England, Wales, and Scotland) in 1 week in 1946, with regular follow-up surveys from birth	74	2015	Three surveys: May, 2020 (68·2%); September–October, 2020 (61·5%); February–March, 2021 (89·9%)	General Health Questionnaire 12	697 valid samples obtained April–June, 2021	1721
**Age heterogeneous studies**							
Understanding Society: the UK Household Longitudinal Survey	A nationally representative longitudinal household panel study, based on a clustered-stratified probability sample of UK households, with all adults aged 16 years or older in selected households surveyed annually	16–96	2018–19	Eight surveys (full or partial interview): April, 2020 (42·0%); May, 2020 (35·1%); June, 2020 (33·5%); July, 2020 (32·6%); September, 2020 (30·6%); November, 2020 (28·6%); January, 2021 (28·5%); March, 2021 (30·2%)	General Health Questionnaire 12	6006 valid samples obtained April–June, 2021	14 154
English Longitudinal Study of Aging	A nationally representative population study of individuals aged 50 years and older living in England, with biennial surveys and periodic refreshing of the sample to maintain representativeness	52–90+*	2018–19	Two surveys: June–July, 2020 (75%); November–December, 2020 (73%)	Centre for Epidemiological Studies Depression Scale	NA	4752
Generation Scotland: the Scottish Family Health Study	A family-structured, population-based Scottish cohort, with participants aged 18–99 years recruited between 2006 and 2011	27–100	2006–11	Three surveys: April–June, 2020 (21·3%); July–August, 2020 (15·4%); February, 2021 (14·3%)	Patient Health Questionnaire 9 and Generalised Anxiety Disorder Assessment 7	NA	3937
Avon Longitudinal Study of Parents and Children-Generation 0	Parents of the Avon Longitudinal Study (G1) cohort, treated as a separate age-heterogenous study population (original parents)	45–81	2011–13	Three surveys: April, 2020 (12·4%); June, 2020 (12·2%); December, 2020 (14·3%)	Short Mood and Feelings Questionnaire	NA	3258
The UK Adult Twin Registry	A cohort of UK volunteer adult twins (55% monozygotic and 43% dizygotic) who were sampled between age 18 and 101 years	22–96	2017–18	Three surveys: July, 2020 (77·6%); November, 2020 (76·1%); March, 2021 (76%)	Hospital and Anxiety Depression Scale	3137 valid samples obtained April, 2021	3137

NA=not applicable.

* This study classified all people older than 90 years in the 90+ category to avoid disclosure of information, given the small number of participants older than 90 years.

Six of the 11 studies were of birth cohorts with all individuals of a similar age: the Millennium Cohort Study (MCS; born 2000–02), the Avon Longitudinal Study of Parents and Children (ALSPAC-G1, born 1990–91), Next Steps (NS, formerly known as the Longitudinal Study of Young People in England; born 1989–90), the 1970 British Cohort Study (BCS70), the National Child Development Study (NCDS; born 1958), and the National Survey of Health and Development (NSHD; born 1946). Five studies were age heterogeneous: Understanding Society/The UK Household Longitudinal Study (USoc/UKHLS), the English Longitudinal Study of Ageing (ELSA), Generation Scotland: The Scottish Family Health Study (GS), the UK Adult Twin Registry (TwinsUK), and the parents of the ALSPAC-G1 birth cohort (ALSPAC-G0).

We included participants in our analytical samples who had information available on at least one mental health outcome measure and at least one report of COVID-19 status during the pandemic. Additionally, we only included participants who had available data on a minimum set of covariates, namely age, sex, and pre-pandemic mental health. Data within studies were weighted to be representative of their target population, accounting for sampling design, attrition up to the most recent pre-pandemic survey, and differential non-response to COVID-19 surveys. Weights were not available for the GS or TwinsUK studies.

Ethics statements for each of the studies and data access details are provided in the appendix (p 5).

All studies collected informed consent from their participants. This study did not seek any additional institutional review board approval.

### Outcomes

Mental health symptoms (psychological distress, depression, anxiety, life satisfaction) were assessed using self-report measures across multiple timepoints of the pandemic. The studies used a variety of measures, which are summarised in the appendix (p 6). The MCS used the Kessler Psychological Distress Scale (K6), a six-item self-administered questionnaire measuring psychological distress over the previous 30 days. Each item in K6 is related to depressive and anxiety symptoms and rated on a 5-point Likert scale, giving a score of 0–24, with a score of >13 indicating probable psychological distress. ALSPAC used the Short Mood and Feelings Questionnaire (SMFQ) and the seven-item Generalised Anxiety Disorder Scale (GAD-7). SMFQ is a 13-item questionnaire that measures depression symptoms in the past 2 weeks, scoring each item as 0–2, with depression rated as none (0–4), mild (5–9), moderate (10–14), moderately severe (15–19), and severe (20–27). GAD-7 is a validated, self-reported measure of anxiety used widely by healthcare professionals, using a 4-point Likert scale for the previous 2 weeks, with a cut-off point of 5 for mild anxiety, 10 for moderate, and 15 for severe anxiety. NS and USoc used the General Health Questionnaire (GHQ), which is self-administered and designed to detect current symptoms of psychological distress (ie, general anxiety and depression). Before the pandemic, the 28-item GHQ was used, and during it the 12-item version was used, scoring each item as 0–3. BCS70 and NCDS used the 9-item version of the Malaise Inventory, which is self-administered and used to assess psychological distress, scoring items as yes (1) or no (0); scores of 4 or more are indicative of probable psychiatric distress. ELSA used the Centre for Epidemiologic Studies Depression Scale (CES-D), an interview in which respondents are asked whether they experienced any depressive symptoms (eg, feeling sad or having restless sleep) in the previous week. For the binary classification, we considered respondents who reported four or more depressive symptoms on the CES-D scale as having elevated depressive symptoms. In GS, depression and anxiety were assessed during the pandemic using the GAD-7 and the Patient Health Questionnaire (PHQ-9). The PHQ-9 is a 9-item, validated tool for the assessment of depressive symptoms (eg, little interest or pleasure in doing things) in the previous 2 weeks, rated on a 4-point Likert scale. A score of 10 or more indicates major depression. GS also used the 28-item GHQ to assess pre-pandemic psychological distress, and so a comparable composite measure was created from the GAD-7 and the PHQ-9 scales to enable evaluation of change over time. TwinsUK used Hospital Anxiety and Depression Scale (HADS), a 14-item questionnaire used to measure the presence of anxiety and depressive symptoms during the previous week in non-psychiatric populations. Responses are on a 4-point ordinal Likert scale ranging from 0 to 3, with a total possible score of 42 (a score of ≥11 indicates moderate to severe levels of depression and anxiety).

We standardised continuous scales within each study to permit comparability of mental health estimates across studies. This standardisation was done by creating Z scores for each continuous mental health measure within each study. We also did analyses with dichotomous indicators using established cut-off scores for each scale to define high levels of psychological distress, depression, and anxiety, and low levels of satisfaction with life.

### Exposures

Self-reported measures of COVID-19 infection, including the date symptoms began and whether infection was test-confirmed (via a PCR, antibody, or lateral flow test) or suspected, were available longitudinally at multiple timepoints during the pandemic. A subset of studies measured serology in subsamples of their participants. We used data from these variables to create exposure variables to answer the different research questions. Self-reported COVID-19 was measured in each study and at each wave, and we used these measures to create a binary, time-updated variable for having ever had COVID-19 (yes or no). We used information about the time since COVID-19 to derive continuous and categorical (no COVID-19, <4 weeks, 4–12 weeks, or ≥12 weeks) variables for the time since infection at each timepoint. We also derived two variables to examine differences between suspected and confirmed COVID-19. First, we created a categorical variable based on self-reported information only: no COVID-19, self-reported suspected COVID-19 (not test-confirmed), or self-reported test-confirmed COVID-19. Second, we used information from a single serology timepoint (home antibody finger-prick test results collected between April and June, 2021 [exact dates not available]) in subsamples of some studies. Using serology data combined with self-report information, we created a categorical variable: no COVID-19, self-reported COVID-19 with negative serology, self-reported COVID-19 with positive serology, and positive serology in the absence of self-reported COVID-19. Data from antibody tests with immunoassay qualitative detection of antibodies against SARS-CoV-2 nucleocapsid (N) protein, of which a positive result (N-assay) is likely to identify natural SARS-CoV-2 infection, were used for the serology. Longitudinal studies with available information on vaccination status used a positive anti-nucleocapsid result at any time or a positive anti-Spike result before vaccination.^22^ In the UK Virus Watch prospective community cohort, anti-N had about 80% sensitivity for detecting prior PCR-confirmed COVID-19 infection.^23^ This sensitivity remained relatively stable for at least 269 days, although concentrations began to decline at about 120 days post-infection.

### Statistical analysis

Where available across studies, models were adjusted for the following covariates: sex (male or female); age (continuous); ethnicity (self-reported and coded into White or non-White ethnic minorities); UK country of residence (England, Scotland, Wales, or Northern Ireland); highest educational qualification (degree or no degree; parental education was used for the MCS cohort, who had not all completed their full-time education); pre-pandemic mental health (continuous); pre-pandemic chronic illness (yes or no); pre-pandemic disability (yes or no); pre-pandemic self-rated health (poor, fair, or good); partnership status (partner or no partner); occupational classification (assessed through the National Statistics Socio-economic Classification and coded into four categories of professional or managerial, intermediate, lower or manual, none or long-term unemployed; appendix p 14). All analyses were adjusted for data collection timepoints during the pandemic to control for overall population level changes in mental health at different stages of the pandemic.

For the analysis, first, we used generalised estimating equations, specifying an unstructured correlation matrix, to examine associations between having ever had COVID-19 and mental health outcomes—psychological distress, depression, anxiety, and life satisfaction—accounting for correlations between repeated measures from the same individuals. For binary mental health outcomes, we used modified Poisson regression with robust standard errors to calculate relative risks.^24^ We ran unadjusted and fully adjusted models for potential confounders.

Second, we examined whether the strength of association varied according to time since infection. We used generalised estimating equation models to examine whether associations between COVID-19 and subsequent mental health varied according to time-since-infection categories: no COVID-19, less than 4 weeks, 4–12 weeks, or 12 weeks or more since infection. Among those with COVID-19, we explored the relationship between continuous time since infection in weeks and mental health. We also ran models incorporating a quadratic term for continuous time since infection, to test for non-linearity.

Third, we tested for interactions between COVID-19 and sex (male or female), ethnicity (White or non-White ethnic minorities), highest educational qualification (degree or non-degree), and pre-pandemic mental health and life satisfaction (using the predefined cutoff points; appendix p 6). We focused on dichotomous pre-pandemic mental health as a potential moderator because we were interested in possible subgroup differences in associations between COVID-19 and subsequent mental health for those above and below clinical case thresholds. We stratified analyses by age in age heterogeneous cohorts, using the following bands: 16–29 years, 30–49 years, 50–69 years, and 70 years and older.

Fourth, using generalised estimating equation models, we examined whether associations between COVID-19 and subsequent mental health differed between suspected versus confirmed infection. Initially, we examined whether associations between COVID-19 and mental health differed between those with self-reported suspected COVID-19 versus self-reported and test-confirmed COVID-19. Additionally, we explored differences in association for those with self-reported suspected COVID-19 versus those with serology-detected SARS-CoV-2. Given the timing of serology assessments, which in many cohorts was alongside or after the most recent mental health assessment and not time varying, we examined associations with mental health at the most recent timepoint only (using linear and modified Poisson regression) for those who: did not have COVID-19 (reference group); self-reported COVID-19 and had serology evidence of SARS-CoV-2; self-reported COVID-19 but had no serology-detected SARS-CoV-2; and did not self-report COVID-19 but had evidence of SARS-CoV-2 infection. In an exploratory analysis, we also tested the association between SARS-CoV-2 serology status (positive *vs* negative) with most recent mental health scores using linear and modified Poisson regression.

We did several sensitivity analyses. In NSHD, NCDS, BCS70, NS, and MCS, participants who reported “unsure” as to whether they had COVID-19 were grouped as COVID-19 cases if they reported an estimated infection date, or as non-cases if they did not report a date. In sensitivity analyses, we compared these results to findings in which those reporting ‘‘unsure’’ were: (1) categorised as not having had COVID-19, and (2) retained as a separate category.

We pooled estimates across studies using random effects meta-analysis with restricted maximum likelihood. Within age heterogeneous cohorts, we pooled estimates from age-stratified analyses using the following age bands: 16–29 years, 30–49 years, 50–69 years, and 70 years and older, and age homogenous cohorts were grouped within the appropriate age band. We reported heterogeneity indices using *I*^2^, and for appropriate cases, T^2^ and 95% prediction intervals (95% PI).^25^ All meta-analyses were conducted using Stata version 17.

### Role of the funding source

The funders of the study had no role in study design, data collection, data analysis, data interpretation, or writing of the report.

## Results

We conducted analyses between Dec 21, 2021, and July, 11, 2022, using 11 different longitudinal studies (*k*), with 54 442 participants in total (including 33 200 [61·0%] women and 21 242 [39·0%] men). Participants ranged from age 16 years (in the USoc study) to aged 90 years and older (in the ELSA study). Eight of 11 studies provided data on ethnicity (n=40 819), including 36 802 (90·2%) participants categorised as White, 3937 (9·6%) as non-White, and 80 (0·2%) as missing. Individual study sample sizes ranged from 1721 in NSHD to 14 154 in USoc. Descriptive statistics for exposures, outcomes, and covariates are presented in the appendix (pp 7–13, 15–17).

By the first survey timepoint (April–June, 2020), between 5·4% (87 of 1432) of participants in NSHD and 19·3% (324 of 1678) in NS self-reported COVID-19 across all studies (note that survey weights have been applied to the percentages presented here). By the final timepoint for each study (Nov 2020–April 2021), between 11·1% (173 of 1536) participants in NSHD and 45·1% (1523 of 3837) in MCS self-reported COVID-19; these percentages are also weighted, and inclusive of the data from the first survey timepoint. Serology data indicated that between 33 (4·7%) of 697 participants in NSHD and 224 (22·7%) of 987 in MCS had positive antibody results, indicating natural SARS-CoV-2 infection. Of those with information on COVID-19 and serology data, between 18 (2·6%) of 697 participants in NSHD and 172 (18·1%) of 952 in MCS had both self-reported and serology-confirmed COVID-19, whereas those with self-reported COVID-19 but negative serology data ranged from 59 (8·5%) in 697 NSHD to 302 (31·7%) of 952 in MCS (appendix p 12). The proportion of people with positive serology data who did not self-report COVID-19 ranged from 2·1% in NSHD to 17·3% in TwinsUK; the overlap between serology and test-confirmed self-reporting ranged from 0·86% in NSHD to 46·1% in NS (appendix p 13).

**Figure 1:**
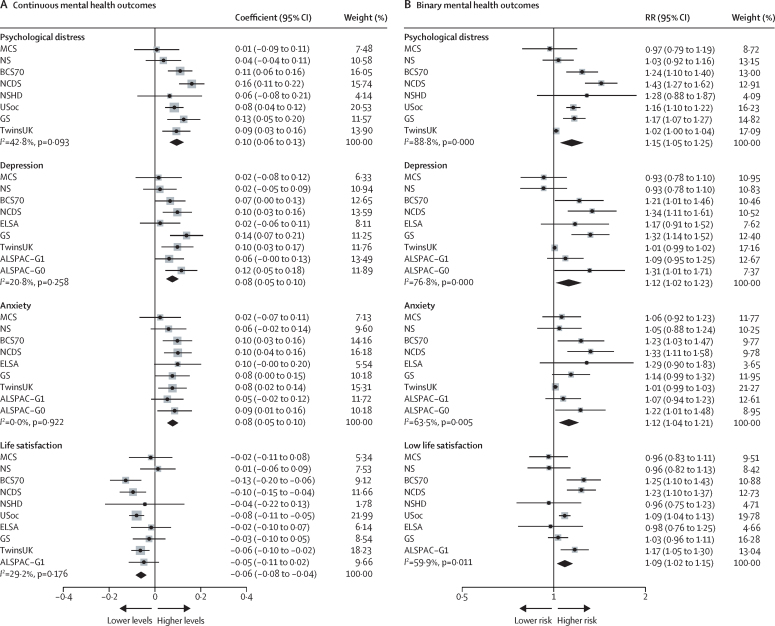
Associations between COVID-19 and continuous (A) and binary (B) mental health outcomes Estimates from longitudinal generalised estimating equation models with ever-COVID-19 exposure and mental health outcomes for each included study and the overall pooled estimate. ALSPAC-G0=the parents of the ALSPAC-G1 birth cohort. ALSPAC-G1=Avon Longitudinal Study of Parents and Children. BCS70= the 1970 British Cohort Study. ELSA=the English Longitudinal Study of Ageing. GS=Generation Scotland: The Scottish Family Health Study. MCS=Millennium Cohort Study. NCDS=the National Child Development Study. NS=Next Steps, formerly known as the Longitudinal Study of Young People in England. NSHD=the National Survey of Health and Development. RR=risk ratio. TwinsUK=the UK Adult Twin Registry. USoc/UKHLS=Understanding Society/The UK Household Longitudinal Study.

Unadjusted results of the associations between COVID-19 and subsequent mental health are presented in the appendix (p 21). Pooled adjusted estimates from meta-analyses indicated that COVID-19 was associated with an increase in subsequent psychological distress (standardised difference in outcome between those with and without self-reported COVID-19: 0·10 [95% CI 0·06 to 0·13], *I*^2^=42·8%; *k*=8), depression (0·08 [0·05 to 0·10], *I*^2^=20·8%; *k*=9), and anxiety (0·08 [0·05 to 0·10], *I*^2^=0·0; *k*=9), and negatively associated with life satisfaction (–0·06 [–0·08 to –0·04], *I*^2^=29·2%; *k*=10; figure 1A). Results were consistent for binary outcomes in terms of effect size and direction (psychological distress RR 1·15 [95% CI 1·05 to 1·25], *I*^2^=88·8%; *k*=8), with associations also found for depression, anxiety, and lower satisfaction with life (figure 1B; appendix p 22). Meta-analysed coefficients for all research questions are reported in the appendix (pp 18–20).

**Figure 2:**
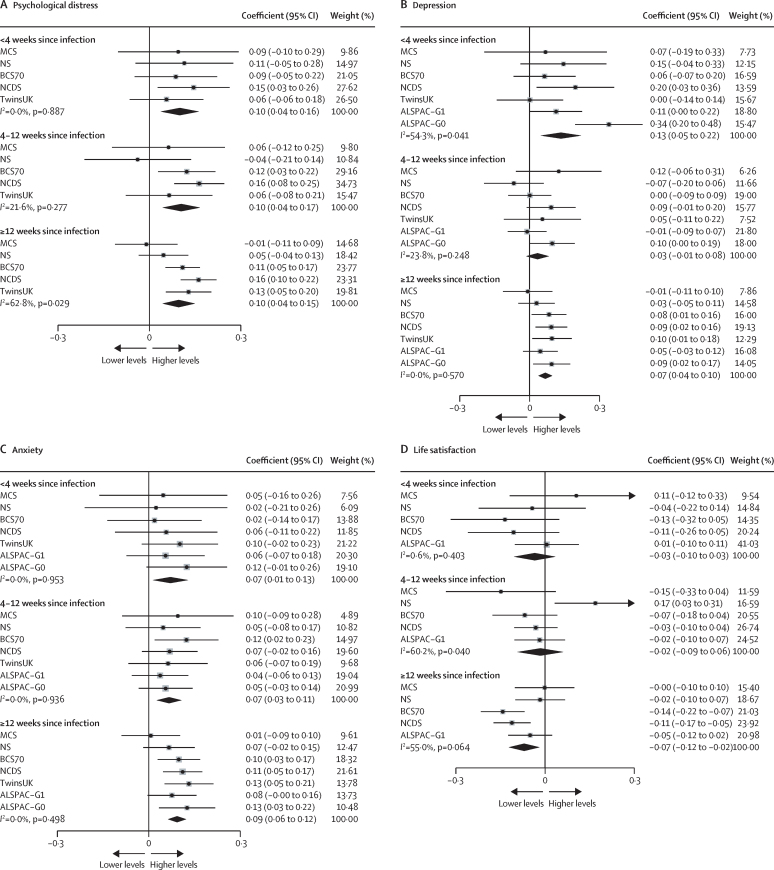
Association between time since COVID-19 infection and continuous mental health outcomes Data for each included study and the overall pooled estimate. ALSPAC-G0=the parents of the ALSPAC-G1 birth cohort. ALSPAC-G1=Avon Longitudinal Study of Parents and Children. BCS70= the 1970 British Cohort Study. NCDS=the National Child Development Study. NS=Next Steps, formerly known as the Longitudinal Study of Young People in England. TwinsUK=the UK Adult Twin Registry.

We examined time since infection using both categorical and continuous variables. Pooled results indicated that the association between COVID-19 and mental health did not differ according to time since infection for psychological distress, with similar associations across duration categories, although heterogeneity increased with time since infection (figure 2). The association between COVID-19 and mental health and time since infection showed similar patterns for depression, anxiety, and life satisfaction (appendix pp 23–26). For those with COVID-19, no association was found between continuous time since infection in weeks and all outcomes. We examined non-linearity with a quadratic term and found no evidence of a non-linear association. The heterogeneity (*I*^2^) between the time-since-infection categories was 18·1%.

When examining potential subgroup differences in associations, we did not find evidence of interactions between COVID-19 and sex, education, ethnicity, or pre-pandemic mental health (appendix pp 27–30). Stratified analyses by age provide some suggestion that effects might be stronger in middle-age and older-age groups. For instance, for depression, the standardised difference in outcome for participants with COVID-19 compared with those without COVID-19 among individuals aged 50–69 years was 0·10 (95% CI 0·06 to 0·15), and for those aged 70 years or older was 0·10 (–0·06 to 0·25), with weaker associations in those aged 16–29 years (0·05, –0·00 to 0·11) and those aged 30–49 years (0·04, –0·03 to 0·10). Similar patterns were found for anxiety. For psychological distress, there was some evidence of a stronger association in the group aged 50–69 years (0·13, 0·10 to 0·15), whereas associations were weaker across other age groups. Associations between COVID-19 and life satisfaction were similar across age groups. However, given the wide confidence intervals we cannot be confident in these age group differences (appendix pp 31–34).

**Figure 3:**
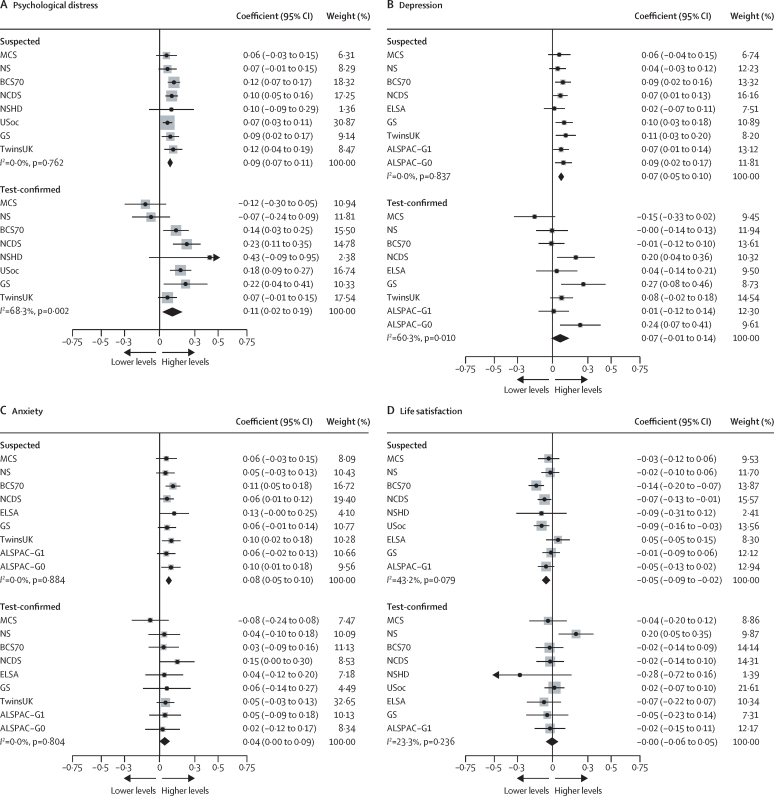
Suspected versus test-confirmed COVID-19 infection and mental health outcomes Data for each included study and the overall pooled estimate. ALSPAC-G0=the parents of the ALSPAC-G1 birth cohort. ALSPAC-G1=Avon Longitudinal Study of Parents and Children. BCS70= the 1970 British Cohort Study. ELSA=the English Longitudinal Study of Ageing. GS=Generation Scotland: The Scottish Family Health Study. MCS=Millennium Cohort Study. NCDS=the National Child Development Study. NS=Next Steps, formerly known as the Longitudinal Study of Young People in England. NSHD=the National Survey of Health and Development. TwinsUK=the UK Adult Twin Registry. USoc/UKHLS=Understanding Society/The UK Household Longitudinal Study.

We examined whether associations differed between participants with suspected COVID-19 versus test-confirmed COVID-19, both based on self-reporting. Both suspected COVID-19 (0·09 [95% CI 0·07 to 0·11], *I*^2^=0·0%; *k*=8) and test-confirmed COVID-19 (0·11 [0·02 to 0·19], *I*^2^=68·3%; *k*=8) were associated with increased psychological distress, with similar patterns for depression and anxiety, although only suspected and not test-confirmed COVID-19 was associated with lower life satisfaction (figure 3; appendix 35–38).

**Figure 4:**
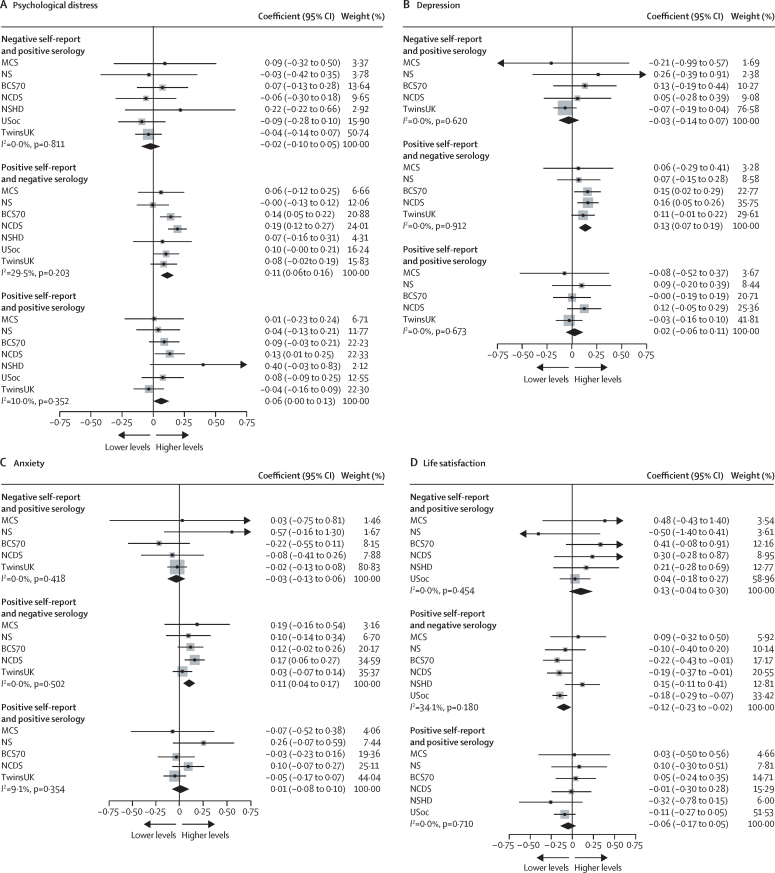
Suspected versus serology-confirmed COVID-19 infection and mental health outcomes Data for each included study and the overall pooled estimate. ALSPAC-G0=the parents of the BCS70= the 1970 British Cohort Study. MCS=Millennium Cohort Study. NCDS=the National Child Development Study. NS=Next Steps, formerly known as the Longitudinal Study of Young People in England. NSHD=the National Survey of Health and Development. TwinsUK=the UK Adult Twin Registry. USoc/UKHLS=Understanding Society/The UK Household Longitudinal Study.

Finally, we examined whether associations varied according to combined self-report and serology data. Participants who self-reported having COVID-19, but had negative serology, had higher levels of psychological distress (0·11 [95% CI 0·06 to 0·16], *I*^2^=29·5%; *k*=7), depression, and anxiety, and lower life satisfaction than those without COVID-19 based on self-report and serology. Associations were not found for those who self-reported having COVID-19 and had positive serology for psychological distress, depression, anxiety, or life satisfaction (figure 4). For participants who had positive serology but did not self-report COVID-19, we did not find associations with any of the mental health outcomes (eg, psychological distress, –0·02 [95% CI –0·10 to 0·05], *I*^2^=0·0%; *k*=7; figure 4; appendix pp 39–42). In a post-hoc exploratory analysis comparing participants with positive serology to those with negative serology, we did not find evidence of differences in psychological distress (0·02 [95% CI –0·03 to 0·07], *I*^2^=12·8%; *k*=7) or other mental health outcomes (appendix p 43).

Sensitivity analyses were done to examine the potential effect of the unsure categorisation in MCS, NS, BCS, NCDS, and NSHD. We did a sensitivity analysis for the first research question on overall association between ever having had COVID-19 and mental health outcomes, grouping those reporting unsure with those who did not report having had COVID-19. Associations remained consistent (psychological distress, 0·07 [95% CI –0·01 to 0·14], *I*^2^=70·0%; appendix p 44–45). In the second sensitivity analysis, both self-reported COVID-19 (psychological distress, 0·10 [95% CI 0·00 to 0·19], *I*^2^=71·0%) and unsure COVID-19 (psychological distress, 0·10 [0·04 to 0·15], *I*^2^=43·8%) showed associations with poorer mental health (appendix p 46–49). Findings are also presented for the second question (on interactions between COVID-19 and sex, ethnicity, educational qualification, and pre-pandemic mental health) and the fourth question (on self-reported suspected infection *vs* self-reported test-confirmed infection *vs* serology-confirmed infection) with different reference groups to provide an alternative comparison and confirm the interpretation (appendix pp 50–61).

## Discussion

Our findings indicate that self-reported COVID-19 illness was associated with a deterioration in mental health outcomes in the UK population. We did not find evidence of change in this association over time during the first few months after infection. Subgroup analysis indicated no differences by sex, ethnicity, education, or pre-pandemic mental health; and there was some indication of a slightly stronger association in some older age groups. Notably, we observed similar associations for both suspected COVID-19 and COVID-19 confirmed by test or serology data, suggesting that the associations could relate to the mental health impacts of illness during this period, rather than exposure to SARS-CoV-2 specifically.

Our findings demonstrate associations between COVID-19 and deterioration in mental health, while controlling for overall effects of timing throughout the first year of the pandemic, adding to existing evidence, which has been mixed to date.^3^, ^4^ The effects observed (6–10% change of a standard deviation for outcomes on a continuous scale and 9–15% increased risk of clinical caseness) have substantial implications when considered at the population level, especially given high infection rates.

We did not observe improvement in mental health in the immediate months after infection.^3^ Studies with longer-term follow-up examining recovery in symptoms are needed to assess the duration of symptoms experienced after infection.

We found that COVID-19 was associated with poorer mental health in all age groups, with some evidence of stronger associations for people aged 50 years and older. This result might reflect that older people are more likely to experience more severe COVID-19 and potentially also greater worry around infection due to their age and higher likelihood of pre-existing health conditions. These findings could also reflect increased risk of microvascular or neurological changes after COVID-19, which have been associated with depression and anxiety phenotypes in older adults.^26^, ^27^, ^28^ However, we found no differences by sex, ethnicity, education level, and previous mental health problems. Previous studies have shown that overall mental health impacts of the pandemic have been greatest in adults aged 25–44 years, women, and those with a degree (*vs* those without a degree),^19^ suggesting that the mechanisms through which COVID-19 illness affects mental health might differ from those underpinning the wider effects of the pandemic.

Our analyses benefited from the use of serology data in addition to information on self-reported COVID-19.^26^ When comparing associations for subgroups on the basis of self-report and serology status, self-reported COVID-19 illness combined with negative serology was associated with poorer mental health, whereas no association was found for positive serology without self-reported COVID-19. Similarly, in a post-hoc exploratory analysis, we did not find evidence of differences in mental health outcomes for those with positive compared with negative serology. These findings align with a population-based cross-sectional study done in France, which found that self-reported COVID-19 infection was associated with a range of persistent physical symptoms, including muscular pain, fatigue, headache, palpitations, dizziness, and cough, whereas laboratory-confirmed COVID-19 was only associated with anosmia.^29^

Various mechanisms have been posited to underlie associations between COVID-19 and psychological distress, including systemic inflammation and changes in the brain associated with COVID-19,^27^ and psychosocial mechanisms including social isolation and worries about possible outcomes and infecting others. One possible explanation for our findings, particularly the association with poorer mental health for those who had negative serology but had self-reported COVID-19, is that contextual and psychosocial aspects of COVID-19 (such as the experience of being unwell and worrying about potential health, social, and economic consequences) are stronger predictors of poor mental health outcomes than any specific neurological consequences of SARS-CoV-2 infection. The lack of association with mental health when serology data detected SARS-CoV-2 infections that had not been reported (ie, cases in which participants were unaware of the infection) support this conclusion.

Nonetheless, it is important to note that participants who self-reported COVID-19 infection but had negative serology status might have correctly identified having had the disease, especially given the known issue of waning of antibodies. In addition, those who self-reported COVID-19 probably experienced related symptoms, whereas those with positive serology who did not self-report symptoms were more likely to have had mild or asymptomatic COVID-19. Participants who suspected COVID-19 with negative serology might have had another respiratory infection with shared pathophysiological pathways, potentially contributing to adverse mental health outcomes. Those who were particularly concerned about consequences of infection might have been more likely to report perceived infection. Notably, only a subset of studies and samples had serology data, substantially reducing power in these analyses. Additionally, only one timepoint of serology assessment was completed after the most recent self-report data, limiting the conclusions that can be drawn. Antibody concentrations following SARS-CoV-2 infection have been found to wane over time,^30^ which could have led to underestimation of COVID-19 based on serology data.

Previous studies have mainly focused on severe COVID-19 in patients admitted to hospital or severe mental illness.^8^, ^10^ Our results add to existing evidence by capturing broader and subclinical mental health impacts of COVID-19 illness in the general population. The use of multiple prospective longitudinal studies allowed us to control for time period effects and important pre-pandemic factors including mental health, physical health, and socio-economic factors. Additionally, we were able to examine the persistence of associations over time and differences between perceived and test-confirmed COVID-19.

The response rates for the COVID-19 surveys in many included studies were not consistently high. However, rich antecedent data in longitudinal prospective studies allowed most studies to be weighted for non-response (aiming to reduce potential bias from selection into analysed samples).^8^, ^10^ Although we were able to control for important confounders, we cannot definitively attribute changes in mental health to COVID-19 illness. Included studies used different measures to assess COVID-19 and mental health outcomes. These were carefully reviewed and harmonised across studies, and in meta-analysis the heterogeneity of estimates between studies was small for most outcomes. Nonetheless, measurement error of the exposure is a potential limitation, given that our main exposure variables were based on self-reported COVID-19, and the other measures of test-confirmed and serology based infection markers are also prone to biases and measurement issues.^8^, ^10^ Further limitations are the lack of data available to examine possible variation in associations by COVID-19 severity, the fact that only infections in the first year of the pandemic have been assessed, and that longer-term follow-up is limited at present.

Our findings suggest that people who self-reported COVID-19 in the first year of the pandemic were subsequently more likely to experience poorer mental health outcomes than those who did not self-report COVID-19. Our findings involving serology-confirmed infection, and the lack of attenuation in association over time, suggest that these associations might not be specific to SARS-CoV-2 infection and potentially reflect consequences of feeling unwell, anxieties related to a novel infection and infecting others, or other factors such as social isolation and loss of pay. Further research is needed to investigate these possible underlying mechanisms and to examine whether associations persist over longer follow-up periods. Our findings emphasise the important population mental health consequences of infection and disease separately from the potential impacts of the pandemic more widely (eg, infection control measures). Given the high prevalence of COVID-19 in the UK and worldwide, these findings have important public health implications, highlighting the need for greater post-infection mental health support in both clinical and community settings.

## Data sharing

Data for NCDS (SN 6137), BCS70 (SN 8547), Next Steps (SN 5545), MCS (SN 8682) and all four COVID-19 surveys (SN 8658) are available through the UK Data Service. NSHD data are available on request to the NSHD Data Sharing Committee. Interested researchers can apply to access the NSHD data via a standard application procedure. Data requests should be submitted to mrclha.swiftinfo@ucl.ac.uk; further details can be found at http://www.nshd.mrc.ac.uk/data.aspx. doi:10·5522/NSHD/Q101; doi:10·5522/NSHD/Q10. Information regarding access to the ALSPAC data can be found on the ALSPAC website (http://www.bristol.ac.uk/media-library/sites/alspac/documents/researchers/data-access/ALSPAC_Access_Policy.pdf). All USoc data are available through the UK Data Service (SN 6614 and SN 8644). All ELSA data are available through the UK Data Service (SN 8688 and 5050). Access to GS data is approved by the Generation Scotland Access Committee. See https://www.ed.ac.uk/generation-scotland/for-researchers/access or email access@generationscotland.org for further details. The TwinsUK Resource Executive Committee oversees management, data sharing, and collaborations involving the TwinsUK registry (for further details see https://twinsuk.ac.uk/resources-for-researchers/access-our-data/).

For the **preregistered analyses on the Open Science Framework** see https://osf.io/ntmqw

## Declaration of interests

SVK is a member of the Scientific Advisory Group on Emergencies subgroup on ethnicity and COVID-19 and is co-chair of the Scottish Government's Ethnicity Reference Group on COVID-19. NC serves on a data safety monitoring board for trials sponsored by AstraZeneca. CJS is an academic lead on KCL Zoe Global COVID symptoms study. KT has been a paid consultant for the CHDI Foundation (member of Statistical Advisory Group) and is a member of the steering group for Living With COVID Recovery study at UCL (unpaid). All other authors declare no competing interests.
