# Supplementary material for: Psychological distress, depression, anxiety, and life satisfaction following COVID-19 infection: evidence from 11 UK longitudinal population studies
Source: Lancet Psychiatry. 2022 Nov;9(11):894–906. doi: 10.1016/S2215-0366(22)00307-8 (PMC9560745; doi:10.1016/S2215-0366(22)00307-8)
Supplement: Supplementary appendix [file mmc1.pdf]

THE LANCET
Psychiatry

Supplementary appendix

This appendix formed part of the original submission and has been peer reviewed. We post it as supplied by the authors.

Supplement to: Thompson EJ, Stafford J, Moltrecht B, et al. Psychological distress, depression, anxiety, and life satisfaction following COVID-19 infection: evidence from 11 UK longitudinal population studies. *Lancet Psychiatry* 2022; **9:** 894–906.

Psychological distress, depression, anxiety and life satisfaction following COVID-19 infection: Evidence from 11 UK longitudinal population studies

Appendix

Table of Contents

Table S1. Ethics and data access statements for each study......5

Table S2. Details of mental health measures in each longitudinal study......6

Table S3. Descriptives for reporting of COVID-19 infection in each wave of data collection and % for the ever-COVID-19 exposure variable per wave of each included study......7

Table S4. Counts and percentages for those self-reporting suspected and confirmed COVID-19 per wave of each included study......9

Table S5. Descriptive statistics for self-reported COVID-19 infection and time since infection......11

Table S6. Descriptives for COVID-19 cases based on self-report and serology data......12

Table S7. Descriptives for COVID-19 cases based on self-report (test-confirmed vs suspected) and serology data......13

Table S8. Covariate adjustment in GEE models......14

Table S9. Sample descriptives......15

Table S10a. Mental health outcomes descriptives......16

Table S10b. Outcome descriptive statistics – Mental health – USOC......17

Table S11. Meta-analysed associations between COVID-19 and continuous mental health outcomes......18

Table S12. Meta-analysed associations between COVID-19 and binary mental health outcomes......20

Figure S1. RQ1) Association between COVID-19 infection and mental health (unadjusted; continuous outcome)......21

Figure S2. RQ1) Association between COVID-19 infection and mental health (unadjusted; binary outcome) 22
Figure S3. RQ2) Time since COVID-19 and psychological distress (adjusted; binary outcome) 23
Figure S4. RQ2) Time since COVID-19 and depression (adjusted; binary outcome) 24
Figure S5. RQ2) Time since COVID-19 and anxiety (adjusted; binary outcome) 25
Figure S6. RQ2) Time since COVID-19 and low life satisfaction (adjusted; binary outcome) 26
Figure S7. RQ3) Interactions with COVID-19 - psychological distress (adjusted; continuous outcome) 27
Figure S8. RQ3) Interactions with COVID-19 - depression (adjusted; continuous outcomes) 28
Figure S9. RQ3) Interactions with COVID-19 - anxiety (adjusted; continuous outcomes) 29
Figure S10. RQ3) Interactions with COVID-19 - life satisfaction (adjusted; continuous outcomes) 30
Figure S11. RQ3) Age stratified association between COVID-19 and psychological distress (adjusted; continuous outcomes) 31
Figure S12. RQ3) Age stratified association between COVID-19 and depression (adjusted; continuous outcomes) 32
Figure S13. RQ3) Age stratified association between COVID-19 and anxiety (adjusted; continuous outcomes) 33
Figure S14. RQ3) Age stratified association between COVID-19 and life satisfaction (adjusted; continuous outcomes) 34
Figure S15. RQ4A) Test-confirmed vs suspected COVID-19 and psychological distress (adjusted; binary outcome) 35
Figure S16. RQ4A) Test-confirmed vs suspected COVID-19 and depression (adjusted; binary outcome) 36
Figure S17. RQ4A) Test-confirmed vs suspected COVID-19 and anxiety (adjusted; binary outcome) 37
Figure S18. RQ4A) Test-confirmed vs suspected COVID-19 and low life satisfaction (adjusted; binary outcome) 38
Figure S19. RQ4B) Serology and self-report - psychological distress (adjusted; binary outcome) 39
Figure S20. RQ4B) Serology and self-report - depression (adjusted; binary outcome) 40
Figure S21. RQ4B) Serology and self-report - anxiety (adjusted; binary outcome) 41

Figure S22. RQ4B) Serology and self-report - low life satisfaction (adjusted; binary outcome) 42
Figure S23. Exploratory analysis – binary serology exposure – positive serology vs negative serology (continuous outcomes) 43
Figure S24. RQ1 restricted to MCS, NS, BCS70, NCDS and NSHD datasets (adjusted; continuous outcome) 44
Figure S25. Sensitivity analysis 1 - Unsure COVID-19 grouped as 'no COVID-19' (adjusted; continuous outcome) 45
Figure S26. Sensitivity analysis 2 - Unsure COVID-19 separate - psychological distress (adjusted; continuous outcome) 46
Figure S27. Sensitivity analysis 2 - Unsure COVID-19 separate - depression (adjusted; continuous outcome) 47
Figure S28. Sensitivity analysis 2 - Unsure C-19 separate - anxiety (adjusted; continuous outcome) 48
Figure S29. Sensitivity analysis 2 - Unsure COVID-19 separate - life satisfaction (adjusted; continuous outcome) 49
Figure S30. Time since COVID-19 and psychological distress (<4 weeks since infection as reference group) 50
Figure S31. Time since COVID-19 and depression (<4 weeks since infection as reference group) 51
Figure S32. Time since COVID-19 and anxiety (<4 weeks since infection as reference group) 52
Figure S33. Time since COVID-19 and life satisfaction (<4 weeks since infection as reference group) 53
Figure S34. Test-confirmed vs suspected COVID-19 and psychological distress (test-confirmed as reference group) 54
Figure S35. Test-confirmed vs suspected COVID-19 and depression (test-confirmed as reference group) 55
Figure S36. Test-confirmed vs suspected COVID-19 and anxiety (test-confirmed as reference group) 56
Figure S37. Test-confirmed vs suspected COVID-19 and life satisfaction (test-confirmed as reference group) 57
Figure S38. Self-report vs serology COVID-19 and psychological distress (positive self-report and positive serology as reference group) 58
Figure S39. Self-report vs serology COVID-19 and depression (positive self-report and positive serology as reference group) 59
Figure S40. Self-report vs serology COVID-19 and anxiety (positive self-report and positive serology as reference group) 60

Figure S41. Self-report vs serology COVID-19 and life satisfaction (positive self-report and positive serology as reference group) . *61*

Supplementary financial information *62*

Search terms for PubMed *64*

STROBE Statement—Checklist of items that should be included in reports of *cohort studies* **65**

Supplementary Tables

Table S1. Ethics and data access statements for each study

**NSHD, NCDS, BCS70, NS** and **MCS**	The most recent sweeps of the **NSHD, NCDS, BCS70**, **Next Steps** and **MCS** have all been granted ethical approval by the National Health Service (NHS) Research Ethics Committee and all participants have given informed consent. Data for NCDS (SN 6137), BCS70 (SN 8547), Next Steps (SN 5545), MCS (SN 8682) and all four COVID-19 surveys (SN 8658) are available through the UK Data Service. NSHD data are available on request to the NSHD Data Sharing Committee. Interested researchers can apply to access the NSHD data via a standard application procedure. Data requests should be submitted to mrclha.swiftinfo@ucl.ac.uk; further details can be found at http://www.nshd.mrc.ac.uk/data.aspx. doi:10.5522/NSHD/Q101; doi:10.5522/NSHD/Q10.
**ALSPAC**	Ethical approval was obtained from the **ALSPAC** Ethics and Law Committee and the Local Research Ethics Committees. The study website contains details of all the data that is available through a fully searchable data dictionary and variable search tool: http://www.bristol.ac.uk/alspac/researchers/our-data. ALSPAC data is available to researchers through an online proposal system. Information regarding access can be found on the ALSPAC website (http://www.bristol.ac.uk/media-library/sites/alspac/documents/researchers/data-access/ALSPAC_Access_Policy.pdf). Study data were collected and managed using REDCap electronic data capture tools hosted at the University of Bristol. REDCap (Research Electronic Data Capture) is a secure, web-based software platform designed to support data capture for research studies.
**USOC**	The University of Essex Ethics Committee has approved all data collection for the **Understanding Society** main study and COVID-19 waves. No additional ethical approval was necessary for this secondary data analysis. All data are available through the UK Data Service (SN 6614 and SN 8644).
**ELSA**	Waves 1-9 of **ELSA** were approved through the National Research Ethics Service, while the COVID-19 Sub-study was approved by the UCL Research Ethics Committee. All participants provided informed consent. All data are available through the UK Data Service (SN 8688 and 5050).
**GS**	**Generation Scotland** obtained ethical approval from the East of Scotland Committee on Medical Research Ethics (on behalf of the National Health Service). Reference number 20/ES/0021. Access to data is approved by the Generation Scotland Access Committee. See https://www.ed.ac.uk/generation-scotland/for-researchers/access or email access@generationscotland.org for further details.
**TWINSUK**	All wave of **TwinsUK** have received ethical approval associated with TwinsUK Biobank (19/NW/0187), TwinsUK (EC04/015) or Healthy Ageing Twin Study (H.A.T.S) (07/H0802/84) studies from NHS Research Ethics Committees at the Department of Twin Research and Genetic Epidemiology, King's College London. The TwinsUK Resource Executive Committee (TREC) oversees management, data sharing and collaborations involving the TwinsUK registry (for further details see https://twinsuk.ac.uk/resources-for-researchers/access-our-data/).

Table S2. Details of mental health measures in each longitudinal study

Study	Details of mental health measures
**MCS**	The K-6 is a 6-item measure of psychological distress (i.e., general anxiety and depression). Responses are rated on a 5-point Likert-type scale, and capture distress over a period of four weeks prior to administration of the scale. Scores range from 0 to 24, with a conservative cut-off of 13+ applied to indicate probable psychological distress.
**ALSPAC**	Self-reported depressive symptoms were measured using the short mood and feelings questionnaire (SMFQ). The SMFQ is a 13-item questionnaire that measures the presence of depression symptoms in the previous two weeks and was administered via postal questionnaire or in research clinics. Each item is scored between 0-2, resulting in a summed score between 0-26. Depression severity can be rated in the following score bands: 0-4 none, 5-9 mild, 10-14 moderate, 15-19 moderately severe, 20-27 severe. ALSPAC also used the seven-item Generalized Anxiety Disorder Scale (GAD-7), which is a validated, self-report measure of anxiety used widely by healthcare professionals. Individuals were asked to consider how frequently they have been bothered by a number of problems in the previous two weeks and respond on a four-point Likert scale from 0 "Not at all" to 3 "Nearly every day". The cut-off points for mild, moderate and severe anxiety, are 5, 10, and 15 respectively, and the maximum score is 21.
**NS**	The 12-item General Health Questionnaire (GHQ) was used to detect symptoms of psychological distress in NS and USoc. The GHQ is a screening instrument designed to detect symptoms of psychological distress (i.e. general anxiety and depression). Each item is scored 0-3 resulting in scores ranging from 0-36. There is an alternative scoring where each item is scored as 0-0-1-1.
**BCS70, NCDS**	The 9-item version of the Malaise Inventory was used to assess general psychological distress. Items are scored using a simple 'Yes/No' response, meaning continuous scores range from 0-9. Scores of four or more are indicative of probable psychiatric distress.
**NSHD**	The General Health Questionnaire (GHQ) was also used to detect symptoms of psychological distress in NSHD. For the pre-pandemic sweep, the 28-item GHQ was used, though the 12-item GHQ was used for the subsequent 3 sweeps. Each item is scored as 0-0-1-1, resulting in scores ranging from 0-12 with higher scores indicating greater likelihood of mental ill health, and a threshold of 4 was used for binary analyses.
**USoc**	The 12-item General Health Questionnaire (GHQ) was used, as described for NS.
**ELSA**	Depressive symptoms were measured using an abbreviated 8-item version of the validated Center for Epidemiologic Studies Depression Scale (CES-D). Respondents were asked whether they had experienced any depressive symptoms, such as feeling sad or having restless sleep, in the week prior to interview. For the binary classification, we considered respondents who reported four or more depressive symptoms on the CES-D scale as having elevated depressive symptoms.
**GS**	Depression and anxiety were assessed during the pandemic sweeps using the GAD-7 and the Patient Health Questionnaire (PHQ-9). The PHQ-9 is a nine-item, validated tool for the assessment of depressive symptoms experienced in the previous two weeks. Participants are asked to indicate how often they have been bothered by problems such as "Little interest or pleasure in doing things?" on a four-point Likert scale from 0 "Not at all" to 3 "Nearly every day". The score is the sum of the nine items, to a total of 27. A score of 10 or more indicates major depression. The 28-item GHQ was used to assess pre-pandemic psychological distress, and so a comparable composite measure was created from the GAD-7 and the PHQ-9 scales to enable evaluation of change over time. Correlations between each GHQ-28 item and items of the GAD-7 and PHQ-9 scales were examined, and the text of the most highly correlated ($r > 0.25$) questions was then reviewed and sense-checked; matched items are presented in the table below. Cut-off scores were determined using ROC curves, which found a general cut-off of >4, a depression cut-off of >5 and an anxiety cut-off of >2. The latter matched the general "proportion" of PHQ-9/GAD-7 scores required for a cut-off.
**TwinsUK**	The Hospital Anxiety and Depression Scale (HADS) is a 14-item scale used to measure levels of psychiatric distress in non-psychiatric patient populations. Responses are indicated on a 4-point ordinal Likert scale.

Further information on specific items, response options and coding for all involved variables in this study can be found here: https://osf.io/x2mu5/

Table S3. Descriptives for reporting of COVID-19 infection in each wave of data collection and % for the ever-COVID-19 exposure variable per wave of each included study

Study	Wave Timing (month-year)	N per wave	N reporting C-19 (per wave)	% Time updated ever- COVID-19
MCS	May-20	2350	320	13.6
MCS	Sept-Oct 2020	2807	954	34.2
MCS	Feb-March 2021	3837	1523	45.1
ALSPAC-G1	April 2020	2011	302	15.0
ALSPAC-G1	May-20	2261	441	19.5
ALSPAC-G1	December 2020	2777	647	23.3
NS	May-20	1678	324	19.3
NS	Sept-Oct 2020	3093	946	30.9
NS	Feb-March 2021	3482	1171	39.8
BCS70	May-20	3,436	661	19.2
BCS70	Sept-Oct 2020	4253	1161	27.8
BCS70	Feb-March 2021	4629	1305	34.6
NCDS	May-20	4456	637	14.3
NCDS	Sept-Oct 2020	5345	1074	20.5
NCDS	Feb-March 2021	5784	1184	26.0
NSHD	May-June 2020	1432	87	5.4
NSHD	Sept-Oct 2020	1677	145	7.6
NSHD	Feb-March 2021	1536	173	11.1
USoC	Apr-20	12,871	1,539	11.5
USoC	May-20	11,642	1,502	12.3
USoC	Jun-20	11,189	1,499	12.7
USoC	Jul-20	10,944	1,551	13.4
USoC	Sep-20	10,332	1,631	15.4
USoC	Nov-20	9,742	1,756	17.7
USoC	Jan-21	9,646	2,109	22.1
USoC	Mar-21	10,150	2,395	23.8
ELSA	June-July 2020	4710	402	9.9
ELSA	Nov-Dec 2020	4889	129	12.4
GS	Apr-20	3937	314	8.0
GS	Jul-20	2924	421	10.7
GS	Feb-21	2728	540	13.7
ALSPAC-G0	April 2020	2774	342	12.3
ALSPAC-G0	May-20	2997	431	14.4
ALSPAC-G0	December 2020	3483	585	16.7

TwinsUK	Jul-20	2437	293	9.3
TwinsUK	Oct-20	2291	308	15.9
TwinsUK	Apr-21	1932	294	19

Note. MCS (Millennium Cohort Study); ALSPAC G1 (Children of the Avon Longitudinal Study of Parents and Children); NS (Next Steps); BCS 70 (1970 British Cohort Study), NCDS (National Child Development Study); USoc (Understanding Society); ELSA (English Longitudinal Study of Ageing); GS (Generation Scotland: the Scottish Family Health Study); ALSPAC G0 (parents of ALSPAC); TwinsUK (UK Adult Twin Registry)

Table S4. Counts and percentages for those self-reporting suspected and confirmed COVID-19 per wave of each included study

Cohort	Wave Timing	Total N	N (%) reporting no C-19		N (%) reporting C-19 suspected		N (%) reporting C-19 confirmed		N reporting unsure as to whether had C-19
MCS_CM	May-20	2350	1710	(72.77)	143	(6.09)	6	(0.26)	491
MCS_CM	Sept-Oct 2020	2807	1853	(66.01)	301	(10.72)	50	(1.78)	603
MCS_CM	Feb-March 2021	3837	2314	(60.31)	461	(12.01)	500	(13.03)	562
MCS_CM (unsure recoded)	May-20	2350	2030	(86.38)	314	(13.36)	6	(0.26)	
MCS_CM (unsure recoded)	Sept-Oct 2020	2807	1853	(66.01)	904	(32.21)	50	(1.78)	
MCS_CM (unsure recoded)	Feb-March 2021	3837	2314	(60.31)	1023	(26.66)	500	(13.03)	
ALSPAC-G1	April 2020	2011	1709	(85)	264	(13.1)	38	(1.9)	
ALSPAC-G1	May-20	2261	1820	(80.5)	395	(17.5)	46	(2)	
ALSPAC-G1	December 2020	2777	2130	(76.7)	499	(18)	148	(5.3)	
NS	May-20	1678	1125	(67.04)	164	(9.77)	12	(0.72)	377
NS	Sept-Oct 2020	3093	2147	(69.41)	318	(10.28)	67	(2.17)	561
NS	Feb-March 2021	3482	2311	(66.37)	371	(10.65)	331	(9.51)	469
NS (unsure recoded)	May-20	1678	1354	(80.69)	312	(18.59)	12	(0.72)	
NS (unsure recoded)	Sept-Oct 2020	3093	2147	(69.41)	879	(28.42)	67	(2.17)	
NS (unsure recoded)	Feb-March 2021	3482	2311	(66.37)	840	(24.12)	331	(9.51)	
BCS70	May-20	3436	2342	(68.16)	291	(8.47)	13	(0.38)	790
BCS70	Sept-Oct 2020	4253	3092	(72.7)	380	(8.93)	63	(1.48)	718
BCS70	Feb-March 2021	4629	3280	(70.86)	415	(8.97)	317	(6.85)	617
BCS70 (unsure recoded)	May-20	3436	2775	(80.76)	648	(18.86)	13	(0.38)	
BCS70 (unsure recoded)	Sept-Oct 2020	4253	3092	(72.7)	1098	(25.82)	63	(1.48)	
BCS70 (unsure recoded)	Feb-March 2021	4629	3324	(71.81)	988	(21.34)	317	(6.85)	
NCDS	May-20	4456	3353	(75.25)	241	(5.41)	17	(0.38)	845
NCDS	Sept-Oct 2020	5345	4271	(79.91)	330	(6.17)	47	(0.88)	697
NCDS	Feb-March 2021	5784	4600	(79.53)	342	(5.91)	258	(4.46)	584
NCDS (unsure recoded)	May-20	4456	3819	(85.7)	620	(13.91)	17	(0.38)	
NCDS (unsure recoded)	Sept-Oct 2020	5345	4271	(79.91)	1027	(19.21)	47	(0.88)	

NSHD	May-June 2020	1432	1278	(89.33)	27	(1.64)	3	(0.1)	124
NSHD	Sept-Oct 2020	1677	1532	(92.00)	43	(2.58)	10	(0.38)	92
NSHD	Feb-March 2021	1536	1396	(87.87)	37	(3.45)	26	(1.29)	77
NSHD (unsure recoded)	May-June 2020	1432	1345	(94.57)	84	(5.32)	3	(0.1)	
NSHD (unsure recoded)	Sept-Oct 2020	1677	1540	(92.77)	127	(6.85)	10	(0.38)	
NSHD (unsure recoded)	Feb-March 2021	1536	1402	(91.45)	108	(7.26)	26	(1.29)	
USoC	Apr-20	12,871	11,375	(88.80)	1,477	(11.00)	19	(0.15)	
USoC	May-20	11,642	11,347	(97.55)	281	(2.28)	14	(0.17)	
USoC	Jun-20	11,189	11,028	(98.62)	140	(1.25)	21	(0.14)	
USoC	Jul-20	10,944	10,817	(98.80)	118	(1.10)	9	(0.07)	
USoC	Sep-20	10,332	10,178	(98.33)	136	(1.50)	18	(0.16)	
USoC	Nov-20	9,742	9,475	(97.20)	130	(1.42)	137	(1.38)	
USoC	Jan-21	9,646	9,214	(95.08)	141	(0.02)	291	(3.42)	
ELSA*	June-July 2020	4710	4308	(90.14)	375	(9.15)	27	(0.68)	
ELSA	Nov-Dec 2020	4889	4760	(96.22)	11	(0.3)	118	(3.47)	
GS	Apr-20	3937	3622	(92.00)	305	(7.75)	9	(0.23)	
GS	Jul-20	2924	2652	(90.70)	260	(8.89)	12	(0.41)	
GS	Feb-21	2728	2420	(88.71)	232	(8.50)	74	(2.71)	
GS	Total	3937	3397	(86.28)	460	(11.68)	80	(2.03)	
ALSPAC-G0	April 2020	2774	2432	(87.7)	319	(11.5)	23	(0.8)	
ALSPAC-G0	May-20	2997	2566	(85.6)	407	(13.6)	24	(0.8)	
ALSPAC-G0	December 2020	3483	2899	(83.2)	498	(14.3)	87	(2.5)	
TwinsUK	Jul-20	2766	2473	(89.4)	270	(9.8)	23	(0.8)	
TwinsUK	Oct-20	2299	1991	(86.6)	163	(7.1)	145	(6.3)	
TwinsUK	Apr-21	1968	1709	(86.8)	166	(8.4)	93	(4.7)	
TwinsUK	Total	3322	2688	(81)	325	(9.8)	307	(9.2)	

*Weighted

Table S5. Descriptive statistics for self-reported COVID-19 infection and time since infection

Study	Wave Timing (month-year)	N per wave	*No COVID-19		*COVID-19 in last 4 weeks		*COVID-19 in last 4-12 weeks		*COVID-19 in last 12+ weeks	
			N (%)		N (%)		N (%)		N (%)	
MCS	May-20	2350	2030	(86.38)	33	(1.4)	188	(8.0)	99	(4.21)
MCS	Sept-Oct 2020	3544	2535	(71.53)	131	(3.7)	39	(1.1)	839	(23.67)
MCS	Feb-March 2021	4633	2708	(58.45)	54	(1.17)	192	(4.14)	1679	(36.24)
ALSPAC-G1	April 2020	2003	1709	(85.3)	121	(6.0)	124	(6.2)	39	(2.5)
ALSPAC-G1	May-20	1856	1538	(82.9)	9	(0.5)	139	(7.5)	170	(9.2)
ALSPAC-G1	December 2020	2646	2234	(84.4)	42	(1.6)	57	(2.2)	313	(11.8)
NS	May-20	1678	1354	(80.69)	37	(2.21)	189	(11.26)	98	(5.84)
NS	Sept-Oct 2020	3384	2400	(70.92)	75	(2.22)	19	(0.56)	890	(26.3)
NS	Feb-March 2021	4069	2522	(61.98)	58	(1.43)	179	(4.4)	1310	(32.19)
BCS70	May-20	3436	2775	(80.76)	77	(2.24)	362	(10.54)	222	(6.46)
BCS70	Sept-Oct 2020	4858	3619	(74.5)	42	(0.86)	21	(0.43)	1176	(24.21)
BCS70	Feb-March 2021	5538	3739	(67.52)	59	(1.07)	178	(3.21)	1562	(28.21)
NCDS	May-20	4456	3819	(85.7)	79	(1.77)	420	(9.43)	138	(3.1)
NCDS	Sept-Oct 2020	5795	4671	(80.6)	16	(0.28)	12	(0.21)	1096	(18.91)
NCDS	Feb-March 2021	6393	4791	(74.94)	55	(0.86)	142	(2.22)	1405	(21.98)
NSHD	May-June 2020	1428	1345	(94.79)	0	(0.00)	36	(2.12)	47	(3.09)
NSHD	Sept-Oct 2020	1674	1532	(92.54)	4	(0.3)	5	(0.26)	133	(6.89)
NSHD	Feb-March 2021	1532	1363	(89.1)	3	(0.19)	5	(0.21)	161	(10.49)
USoC	Apr-20	12,955	11,311	(87.3)	1,625	(13)	19	(0.00)	0	(0.00)
USoC	May-20	11,730	10,141	(86.5)	167	(1.4)	1,422	(12.1)	0	(0.00)
USoC	Jun-20	11,184	9,631	(86.1)	94	(0.8)	194	(1.7)	1,265	(11.3)
USoC	Jul-20	10,892	9,278	(85.1)	80	(0.7)	258	(2.4)	1,276	(11.7)
USoC	Sep-20	10,138	8,458	(83.4)	1	(0)	281	(2.8)	1,398	(13.8)
USoC	Nov-20	9,588	7,794	(81.3)	10	(0.1)	258	(2.7)	1,526	(15.9)
USoC	Jan-21	9,432	7,297	(77.4)	0	(0)	376	(4.00)	1,759	(18.7)
USoC	Mar-21	10,010	7,567	(75.6)	84	(0.8)	72	(0.7)	2,287	(22.9)
ALSPAC-G0	April 2020	2740	2432	(88.8)	75	(2.7)	156	(5.7)	77	(2.8)
ALSPAC-G0	May-20	2079	1737	(83.6)	<5**	(NA)	147	(7.1)	191	(9.2)
ALSPAC-G0	December 2020	3168	2850	(89)	36	(1.1)	36	(1.1)	246	(7.8)
TwinsUK	Jul-20	3001	2704	(90.1)	29	(0.96)	5	(0.2)	263	(8.0)
TwinsUK	Oct-20	2799	2406	(85.96)	25	(0.8)	21	(0.6)	153	(4.6)
TwinsUK	Apr-21	2370	2023	(85.36)	109	(3.3)	131	(4.0)	40	(1.2)

*After first wave of assessment values are based on “updated ever had COVID-19 infection” variable. **Collapsed due to small cell counts and potentially identifiable data in ALSPAC.

Table S6. Descriptives for COVID-19 cases based on self-report and serology data

Study	N with self-report and serology measures	Serology measure	Both self-report and serology positive	Both self-report and serology negative	Self-reported COVID-19, negative serology	No self-reported COVID-19, positive serology
			N (%)	N (%)	N (%)	N (%)
MCS	952	Antibody tests with immunoassay qualitative detection of antibodies against SARS-CoV-2 nucleocapsid (N) protein - positive result (N-assay)	172 (18.07)	436 (45.8)	302 (31.72)	42 (4.41)
NS	1007	As above	128 (12.71)	554 (55.01)	299 (29.69)	26 (2.58)
BCS70	1927	As above	162 (8.41)	1328 (68.92)	384 (19.93)	53 (2.75)
NCDS	2643	As above	176 (6.66)	1848 (69.92)	573 (21.68)	46 (1.74)
NSHD	697	As above	18 (2.58)	605 (86.8)	59 (8.46)	15 (2.15)
USoC	4,867	As above	337 (8.24)	3544 (69.97)	851 (18.89)	135 (2.9)
TwinsUK	3137	Antibody tests with immunoassay qualitative detection of antibodies against SARS-CoV-2 nucleocapsid (N) protein - positive result (N-assay) or a positive anti-Spike result prior to vaccination definition	263 (8.84)	1998 (63.7)	333 (10.6)	543 (17.3)

Note. MCS (Millennium Cohort Study); NS (Next Steps); BCS 70 (1970 British Cohort Study), NCDS (National Child Development Study); USoc (Understanding Society); GS (Generation Scotland: the Scottish Family Health Study); TwinsUK (UK Adult Twin Registry); CDC: Centers for Disease Control and Prevention

Table S7. Descriptives for COVID-19 cases based on self-report (test-confirmed vs suspected) and serology data

Study	N with suspected vs test-confirmed (self-report) and serology measures	No Covid-19 (self-report test-confirmed or suspected), positive serology		No Covid-19 (self-report test-confirmed or suspected), negative serology		Suspected COVID-19 (self-report), positive serology		Suspected COVID-19 (self-report), negative serology		Test-confirmed (self-reported) COVID-19, positive serology		Test-confirmed (self-reported) COVID-19, negative serology	
	**N**	**N**	**%**	**N**	**%**	**N**	**%**	**N**	**%**	**N**	**%**	**N**	**%**
MCS	952	42	19.63	436	59.08	78	36.45	289	39.16	94	43.93	13	1.76
NS	1007	26	16.88	554	64.95	57	37.01	291	34.11	71	46.1	8	0.94
BC70	2072	54	23.38	1284	69.74	81	35.06	546	29.66	96	41.56	11	0.6
NCDS	2643	46	20.72	1848	76.33	91	40.99	566	23.38	85	38.29	7	0.29
NSHD	697	15	2.15	605	86.8	12	1.72	58	8.32	<10	0.86	<10	0.14
USoc	4,867	143	3.32	3,794	75.49	119	2.53	579	12.65	210	5.29	22	0.73
TwinsUK	3137	543	17.3	1998	63.7	70	8.7	239	10.3	193	23.9	94	0.4

Table S8. Covariate adjustment in GEE models

Study	Fully adjusted model covariates	Exceptions and notes
**MCS**	Sex, parent education, country of residence, ethnicity, time period of pandemic, occupational social class, prior chronic health conditions and disabilities, pre-pandemic mental health and pre-pandemic self-rated health	"Country of residence" omitted due to non-convergence in interaction model by pre-pandemic mental health.
**ALSPAC-G1**	Sex, education, ethnicity, time period of pandemic, parental social class, living alone status, pre-pandemic mental health and pre-pandemic life-satisfaction (wellbeing), pre-pandemic self-rated health	Pre-pandemic life-satisfaction was assessed using mental wellbeing measures
**NS**	Sex, education, country of residence, ethnicity, time period of pandemic, occupational social class, partnership status, prior chronic health conditions, pre-pandemic mental health and pre-pandemic life-satisfaction, pre-pandemic self-rated health	"Country of residence" omitted due to non-convergence in models with binary outcomes for satisfaction with life and interaction terms + binary outcomes
**BCS70**	Sex, education, country of residence, time period of pandemic, occupational social class, partnership status, prior chronic health conditions and disabilities, pre-pandemic mental health, pre-pandemic life-satisfaction, pre-pandemic self-rated health	
**NCDS**	Sex, education, country of residence, time period of pandemic, occupational social class, partnership status, prior chronic health conditions, pre-pandemic mental health, pre-pandemic life-satisfaction, pre-pandemic self-rated health	"Country of residence" omitted due to non-convergence in interaction models with binary outcomes.
**NSHD**	Sex, education, country of residence, time period of pandemic, occupational social class, partnership status, prior chronic health conditions and disabilities, pre-pandemic mental health and pre-pandemic life-satisfaction pre-pandemic self-rated health	RQ2 - for binary life satisfaction outcome, country variable excluded due to non-convergence
**USoc**	Age, sex, country of residence, time period of pandemic, education, ethnicity, partnership status, pre-pandemic limiting longstanding illness, pandemic disability, pandemic self-related health, pre-pandemic psychological distress, pre-pandemic life-satisfaction.	Pre-pandemic measures were continuous/ binary depending on operationalisation of the outcome.
**ALSPAC-G0**	Age, sex, education, ethnicity, time period of pandemic, social class, living alone status, pre-pandemic mental health, pre-pandemic self-rated health	
**ELSA**	Age (categorical), sex, time period of pandemic, education, ethnicity, partnership status, occupational social class, limiting longstanding illness, self-related health, pre-pandemic psychological distress, pre-pandemic life-satisfaction, pre-pandemic anxiety	Pre-pandemic measures were continuous/ binary depending on operationalisation of the outcome.
**GS**	Age (categorical), sex, time period of pandemic, education, ethnicity, partnership status, occupational social class, pre-pandemic psychological distress	Pre-pandemic measures were continuous/ binary depending on operationalisation of the outcome
**TwinsUK**	Age, sex, time period of the pandemic, country, education, ethnicity, partnership status, pandemic disability, pre-pandemic self-rated health, pre-pandemic psychological distress	Pre-pandemic measures were continuous/ binary depending on operationalisation of the outcome

Table S9. Sample descriptives

Characteristics (n (%))	MCS	ALSPAC-G1	NS	BCS 70	NCDS	NSHD	USoc	ALSPAC-G0	ELSA	GS	TwinsUK
**Analytic sample**	4652	2498	4092	5545	6696	1721	14154	3258	4752	3937	3137
**Sex**											
Men	1845 (54.97)	779 (31.2)	1530 (37.39)	2425 (43.73)	3157 (43.73)	810 (47.1)	5966 (47.7)	809 (24.8)	2127 (44.8)	1480 (37.6)	314 (10)
Women	2807 (45.02)	1719 (68.8)	2562 (62.61)	3120 (56.27)	3539 (56.27)	911 (52.9)	8188 (52.3)	2449 (75.2)	2625 (55.2)	2457 (62.4)	2823 (90)
**Age group**											
16-29	5014	2498 (100)	-	-	-	-	1573 (18.6)	-	909 (19.1)	38 (1.0)	42 (1.3)
30-49	-	-	4092 (100)	-	-	-	4256 (28.8)	57 (1.8)	1515 (31.9)	781 (19.8)	409 (13)
50-69	-	-	-	5545 (100)	6696 (100)	-	5951 (33.9)	3140 (96.4	1356 (28.5)	2295 (58.3)	1420 (45.3)
70+	-	-	-	-	-	1721 (100)	2374 (18.7)	61 (1.9)	972 (20.5)	823 (20.9)	1266 (40.4)
**Education**											
Degree	2745 (54.75)	1621 (64.9)	2088 (51.03)	2557 (46.11)	2801 (41.83)	444 (25.8)	5931 (33.3)	946 (29)	3698 (77.8)	1857 (47.2)	1552 (49.5)
Non-Degree	1,927 (38.43)	877 (35.1)	2004 (48.97)	2988 (53.89)	3596 (53.70)	1277 (74.2)	6543 (56.1)	2312 (71)	1054 (22.2)	2080 (52.8)	1462 (46.6)
missing	342 (6.82)	-	-	-	299 (4.47)	-	1680 (10.6)	-	-	-	123 (3.9)
**Ethnicity**											
White	4179 (83.35)	2424 (97)	2,997 (74.28)	-	-	-	12559 (92.4)	3206 (98.4)	4458 (93.8)	3912 (99.4)	3077 (98.1)
Non-White	812 (16.19)	74 (3)	1038 (25.72)	-	-	-	1595 (7.6)	52 (1.6)	294 (6.2)	25 (0.6)	47 (1.5)
missing	23 (0.46)	57	-	-	-	-	-	-	-	-	13 (0.4)
**Pre-pandemic mental health**											
Clin. symptomatic	903 (18.01)	576 (23.1)	1057 (25.83)	982 (17.71)	855 (12.77)	204 (11.9)	2574 (20.2)	592 (18.2)	552 (11.6)	676 (17.2)	200 (6.4)
Non symptomatic	4111 (81.99)	1922 (76.9)	3035 (74.17)	4,563 (82.29)	5841 (87.23)	1517 (88.1)	11580 (79.8)	2666 (81.8)	4200 (88.4)	3261 (82.8)	2937 (93.6)
**Pre-pandemic life satisfaction**											
Satisfied	-	-	3710 (90.71)	4821 (87.18)	5058 (79.07)	-	10745 (72.4)	-	-	-	-
Not Satisfied	-	-	380 (9.29)	709 (12.92)	1339 (20.93)	-	3409 (27.7)	-	-	-	-

Table S10a. Mental health outcomes descriptives

			Time 1			Time 2			Time 3		
**Longitudinal study**	**Mental health outcome**	**Measure**	***N***	**Mean score (SD)**	**High symptoms (%)**	***N***	**Mean score (SD)**	**High symptoms (%)**	***N***	**Mean score (SD)**	**High symptoms (%)**
**MCS**	Psychological distress	K-6	2,260	7.98 (5.09)	18.98	2,813	8.46 (5.52)	24.75	4,051	8.32 (5.73)	24.66
	Depression	PHQ-2	2,259	1.84 (1.65)	28.78	2,805	1.75 (1.70)	27.31	4,050	1.90 (1.75)	30.01
	Anxiety	GAD-2	2,257	1.73 (1.79)	26.44	2,806	2.02 (1.87)	31.70	4,051	1.97 (1.79)	30.02
	Life satisfaction	SWL	2,291	2,291 (2.21)	35.06	2,834	6.34 (2.09)	30.72	4,075	5.89 (2.27)	41.24
**ALSPAC-G1**	Depression	SMFQ	1789	5.94 (5.36)	16.34	1623	6.23 (5.57)	19.70	2337	6.36 (5.97)	20.4
	Anxiety	GAD-7	1788	6.0 (5.2)	22.82	1623	5.76 (5.08)	21.38	2343	6.48 (5.37)	26.12
	Life satisfaction	WEMWBS	1780	44.55 (8.28)	29.27	1628	44.76 (8.53)	29.91	2327	45.18 (9.31)	29.52
**NS**	Psychological distress	GHQ-12	1,544	13.46 (6.03)	35.52	3,105	13.23 (5.74)	30.52	3052	13.40 (5.85)	34.40
	Depression	PHQ-2	1,567	1.41 (1.56)	16.65	3,117	1.29 (1.63)	18.66	3,569	1.42 (1.59)	20.13
	Anxiety	GAD-2	1,570	1.43 (1.68)	18.98	3,119	1.59 (1.73)	22.48	3,568	1.51 (1.76)	21.55
	Life satisfaction	SWL	1,585	6.95 (2.06)	20.52	3,131	6.81(2.17)	21.96	3,581	6.53 (2.21)	28.93
**BCS70**	Psychological distress	MAL	3,276	1.79 (2.00)	18.10	4169	2.08 (2.17)	22.73	4528	1.92 (2.11)	21.19
	Depression	PHQ-2	3284	0.99 (1.46)	12.65	4170	0.96 (1.50)	12.40	4532	1.05 (1.50)	13.26
	Anxiety	GAD-2	3280	0.99 (1.42)	11.28	4170	1.06 (1.52)	14.03	4535	0.97 (1.49)	12.02
	Life satisfaction	SWL	3303	7.09 (2.01)	19.73	4,184	6.93 (2.10)	23.38	4,547	6.84 (2.11)	24.08
**NCDS**	Psychological distress	MAL	4,325	1.35 (1.82)	12.21	5,507	1.61(1.97)	15.87	5,949	1.50 (1.91)	14.77
	Depression	PHQ-2	4,367	0.71 (1.32)	8.12	5,502	0.70(1.28)	7.90	5,968	0.87 (1.42)	11.71
	Anxiety	GAD-2	4,355	0.77 (1.36)	9.26	5,508	0.80(1.33)	8.29	5,975	0.82 (1.43)	9.50
	Life satisfaction	SWL	4,400	7.36 (2.12)	14.79	5,519	7.34(1.96)	16.28	5,985	7.07 (2.12)	20.80
**NSHD**	Psychological distress	GHQ-12	1324	2.06 (2.51)	20.11	1625	1.91 (2.53)	20.88	1507	1.76 (2.84)	18.04
	Life satisfaction	SWL	1024	7.61 (1.96)	23.19	1664	7.01 (2.19)	34.84	1524	6.83 (2.26)	38.85
**USOC**	Psychological distress	GHQ-12					See Table S10b below				
	Life satisfaction	SWL					See Table S10b below				
**ELSA**	Depression	CESD	4889	1.9 (2.1)	19.95	4889	2.3 (2.4)	27.08	NA	NA	NA
	Anxiety	GAD-7	4889	3.2 (4.2)	8.52	4889	3.6 (4.5)	10.77	NA	NA	NA
	Life satisfaction	SWL	4889	7.0 (2.2)	33.45	4889	6.9 (2.2)	36.14	NA	NA	NA
**GS**	Psychological distress	PHQ-9 and GAD-7	3776	3.56 (3.87)	28.5	2784	3.30 (3.76)	25.2	2571	4.17 (4.05)	33.2
	Depression	PHQ-9 (subset)	3836	2.33 (2.74)	11.8	2831	2.33 (2.75)	11.6	2612	2.85 (2.89)	15.7
	Anxiety	GAD-7 (subset)	3854	1.27 (1.48)	15.9	2847	1.02 (1.36)	11.5	2642	1.35 (1.51)	17.6
	Life satisfaction	SWL	3927	6.44 (2.27)	44.4	2898	7.14 (2.00)	29.6	2698	6.17 (2.22)	47.9
**ALSPAC-G0**	Depression	SMFQ	2461	2.90 (3.59)	4.06	2490	3.17 (3.89)	5.54	2881	3.31 (3.94)	6.0
	Anxiety	GAD-7	2502	3.41 (4.09)	9.1	2529	3.25 (4.15)	8.62	2905	4.22 (4.55)	13.46
**TWINS UK**	Psychological distress	HADS	2765	10.2 (5.52)	7.02	2623	10.5 (5.77)	10.23	2085	9.04 (6.26)	179
	Depression	HADS	2764	5.28 (2.32)	1.92	2620	5.55 (2.50)	2.67	2090	4.46 (3.11)	3.25
	Anxiety	HADS	2758	4.91 (3.79)	6.24	2617	4.95 (3.86)	5.54	2161	4.66 (3.86)	67.10
	Life satisfaction	SWL	NA	NA	NA	2763	7.28 (2.01)	25.0	2631	7.38 (1.92)	22.3

NA = Not available, K-6= Kessler psychological distress scale, PHQ=Patient Health Questionnaire, GAD= Generalised anxiety disorder questionnaire, SWL= Satisfaction with Life (1 item), SMFQ= Short Mood and Feelings Questionnaire, WEMWBS = Warwick-Edinburgh Mental Wellbeing Scales, GHQ= General Health Questionnaire, MAL= Malaise Inventory, CES= Centre for Epidemiologic Studies Depression Scale, HADS= Hospital Anxiety and Depression Scale

Table S10b. Outcome descriptive statistics – Mental health – USOC

	Psychological distress – GHQ12			Life satisfaction - SWL 0-7 Scale		
Time	*N*	Mean score (SD)	High symptoms (%)	*N*	Mean score (SD)	High symptoms (%)
April 2020	12,548	12.62 (6.20)	29.94			
May 2020	11,473	12.45 (6.12)	26.70	11,511	4.80 (1.55)	36.28
June 2020	10,975	12.56 (6.23)	25.62			
July 2020	10,751	11.79 (5.75)	20.65	10,783	4.94 (1.58)	31.14
Sept 2020	10,042	11.89 (5.71)	20.92	10,070	4.87 (1.51)	33.37
Nov 2020	9,497	12.84 (6.18)	26.07	9,524	4.88 (1.52)	32.78
Jan 2021	9,360	12.82 (6.14)	26.72	9,393	4.65 (1.53)	40.14
March 2021	10,038	12.49 (6.06)	24.00	10,069	4.86 (1.52)	33.05

Table S11. Meta-analysed associations between COVID-19 and continuous mental health outcomes

		Mental health outcome			
		**Psychological distress**	**Depression**	**Anxiety**	**Life satisfaction**
		Coefficient (95% CI)	Coefficient (95% CI)	Coefficient (95% CI)	Coefficient (95% CI)
**RQ1) COVID-19 (ref. no COVID-19)**	**COVID-19 (unadjusted)**	0.13 (0.08 - 0.18)	0.11 (0.06 – 0.17)	0.11 (0.08 – 0.13)	-0.09 (-0.12 – -0.06)
	% I2 & T^2	70.6 ; 0.00	79.1 ; 0.00	26.9 ; 0.00	52.9 ; 0.00
	95% PI	-0.03 – 0.29	-0.08 – 0.30	0.04 – 0.16	-0.17 – - 0.00
	**COVID-19 (adjusted)**	0.10 (0.06 – 0.13)	0.08 (0.05 – 0.10)	0.08 (0.05 – 0.10)	-0.06 (-0.08 – -0.04)
	% I2 & T^2	42.8 ; 0.00	20.8 ; 0.00	0 ; 0.00	29.2 ; 0.00
	95% PI	0.01 - 0.17	0.02 - 0.12	0.04 - 0.10	-0.11 – -0.00
**RQ2) Duration (ref. no COVID-19)**	**<4 weeks**	0.10 (0.04 – 0.16)	0.13 (0.05 – 0.22)	0.07 (0.01 – 0.13)	-0.03 (-0.10 – 0.03)
	% I2	0	54.3	0	0.6
	**4-12 weeks**	0.10 (0.04 – 0.17)	0.03 (-0.01 – 0.08)	0.07 (0.03 – 0.11)	-0.02 (-0.09 – 0.06)
	% I2	21.6	23.8	0	60.2
	**12+ weeks**	0.10 (0.04 – 0.15)	0.07 (0.04 – 0.10)	0.09 (0.06 – 0.12)	-0.07 (-0.12 – -0.02)
	% I2	62.8	0	0	55
	**Duration in weeks (continuous)**	-0.0001 (-0.004 – 0.002)	0.001 (-0.002 – 0.003)	0.001 (-0.001 – 0.002)	-0.001 (-0.003 – 0.002)
	% I2 & T^2	53.7 ; 0.00	43.5; 0.00	0.01 ; 0.00	55.36; 0.00
	95% PI	-0.009 – 0.007	-0.002 – 0.003	-0.001 – 0.003	-0.008 – 0.007
**RQ3) Age stratified (ref. no COVID-19)**	**16-29**	0.08 (-0.05 - 0.20)	0.05 (-0.00 – 0.11)	0.04 (-0.02 – 0.10)	-0.06 (-0.11 - -0.01)
	% I2	38.5	0	9.5	0
	**30-49**	0.06 (0.01 – 0.10)	0.04 (-0.03 – 0.10)	0.04 (-0.04 – 0.12)	-0.03 (-0.11 – 0.05)
	% I2	0	0	17.9	65
	**50-69**	0.13 (0.10 – 0.15)	0.10 (0.06 – 0.15)	0.10 (0.06 – 0.13)	-0.07 (-0.11 – 0.04)
	% I2	0	44.2	0	30.1
	**70+**	0.08 (0.02 – 0.13)	0.10 (-0.06 – 0.15)	0.10 (-0.03 – 0.23)	-0.07 (-0.15 – 0.01)
	% I2	0	71.6	49.8	0
**RQ3) Interactions (ref. no COVID-19)**	**Education**	0.03 (-0.04 – 0.10)	0.02 (-0.05 – 0.08)	0.02 (-0.06 – 0.11)	0.00 (-0.06 – 0.07)
	% I2	57	47	71.4	46.7
	**Ethnicity**	-0.00 (-0.14 – 0.13)	-0.03 (-0.19 – 0.14)	-0.01 (-0.19 – 0.17)	0.08 (-0.02 – 0.18)
	% I2	0	24.9	58.7	0
	**Prior mental health**	0.06 (-0.04 – 0.17)	-0.00 (-0.09 – 0.09)	0.06 (-0.04 – 0.15)	-0.04 (-0.13 – 0.05)
	% I2	45.2	49	19.5	23.8
	**Prior life satisfaction**	-	-	-	0.04 (-0.02, 0.10)
	% I2	-	-	-	0
	**Sex**	0.04 (-0.01 – 0.08)	0.03 (-0.01 – 0.07)	0.03 (-0.02 – 0.09)	0.00 (-0.04 – 0.05)
	% I2	32.3	0	35.3	0
**RQ4A) Test-confirmed vs suspected (ref. no COVID-19)**	**Suspected COVID-19**	0.09 (0.07 – 0.11)	0.07 (0.05 – 0.10)	0.08 (0.05 – 0.10)	-0.05 (-0.09 - -0.02)
	% I2	0	0	0	43.2
	**Test-confirmed COVID-19**	0.11 (0.02 – 0.19)	0.07 (-0.01 – 0.14)	0.04 (0.00 – 0.09)	-0.00 (-0.06 – 0.05)
	% I2	68.3	60.3	0	23.3

**RQ4B) Serology vs self-report (ref. no COVID-19)**	**No self-report case, positive serology-**	-0.02 (-0.10 – 0.05)	-0.03 (-0.14 – 0.07)	-0.03 (-0.13 – 0.06)	0.13 (-0.04 – 0.30)
	% I2	0	0	0	0
	**Self-report case, negative serology**	0.11 (0.06 – 0.16)	0.13 (0.07 – 0.19)	0.11 (0.04 – 0.17)	-0.12 (-0.23 – -0.02)
	% I2	29.5	0	0	34.1
	**Self-report case, positive serology**	0.06 (0.00 – 0.13)	0.02 (-0.06 – 0.11)	0.01 (-0.08 – 0.10)	-0.06 (-0.17 – 0.05)
	% I2	10	0	9.1	0

Table S12. Meta-analysed associations between COVID-19 and binary mental health outcomes

		Mental health outcome			
		Psychological distress	Depression	Anxiety	Life satisfaction
		RR (95% CI)	RR (95% CI)	RR (95% CI)	RR (95% CI)
**RQ1) COVID-19 (ref. no COVID-19)**	**COVID-19 (unadjusted)**	1.21 (1.09 – 1.36)	1.21 (1.07 – 1.37)	1.20 (1.09 – 1.32)	1.11 (1.06 – 1.17)
	% I2	93	90	84.2	45.7
	**COVID-19 (adjusted)**	1.15 (1.05 – 1.25)	1.12 (1.02 – 1.23)	1.12 (1.04 – 1.21)	1.09 (1.02 – 1.15)
	% I2	88.8	76.8	63.5	59.9
**RQ2) Duration (ref. no COVID-19)**	**<4 weeks**	1.12 (0.95 – 1.32)	1.28 (1.02 – 1.60)	1.02 (0.98 – 1.06)	1.08 (0.88 – 1.33)
	% I2	47.3	70.3	0	46.6
	**4-12 weeks**	1.12 (0.98 – 1.27)	1.03 (0.92 – 1.16)	1.05 (0.98 – 1.12)	1.05 (0.90 – 1.22)
	% I2	59.3	42.7	7	56.1
	**12+ weeks**	1.13 (0.98 – 1.31)	1.05 (0.96 – 1.16)	1.14 (1.03 – 1.26)	1.12 (1.02 – 1.24)
	% I2	88.4	58.4	72.4	59.7
**RQ4A) Test-confirmed vs suspected (ref. no COVID-19)**	**Suspected COVID-19**	1.14 (1.05 – 1.24)	1.14 (1.04 – 1.24)	1.13 (1.04 – 1.22)	1.10 (1.05 – 1.15)
	% I2	83.9	67.5	62.8	21.2
	**Test-confirmed COVID-19**	1.20 (1.03 – 1.39)	1.09 (0.93 – 1.26)	1.04 (0.98 – 1.11)	1.05 (0.97 – 1.13)
	% I2	83.9	63.2	8.2	11
**RQ4B) Serology vs self-report (ref. no COVID-19)**	**No self-report case, positive serology**	1.01 (0.74 – 1.38)	1.10 (0.66 – 1.83)	1.20 (0.74 – 1.94)	1.02 (0.79 – 1.30)
	% I2	0	0	0	0
	**Self-report case, negative serology**	1.22 (1.08 – 1.39)	1.22 (1.01 – 1.48)	1.28 (1.07 – 1.54)	1.18 (1.05 – 1.33)
	% I2	25	4.7	0	29
	**Self-report case, positive serology**	1.20 (1.02 – 1.40)	1.10 (0.84 – 1.44)	1.12 (0.82 – 1.54)	1.02 (0.87 – 1.18)
	% I2	0	0	26	0

Supplementary Figures - Forest plots

Figure S1. RQ1) Association between COVID-19 infection and mental health (unadjusted; continuous outcome)

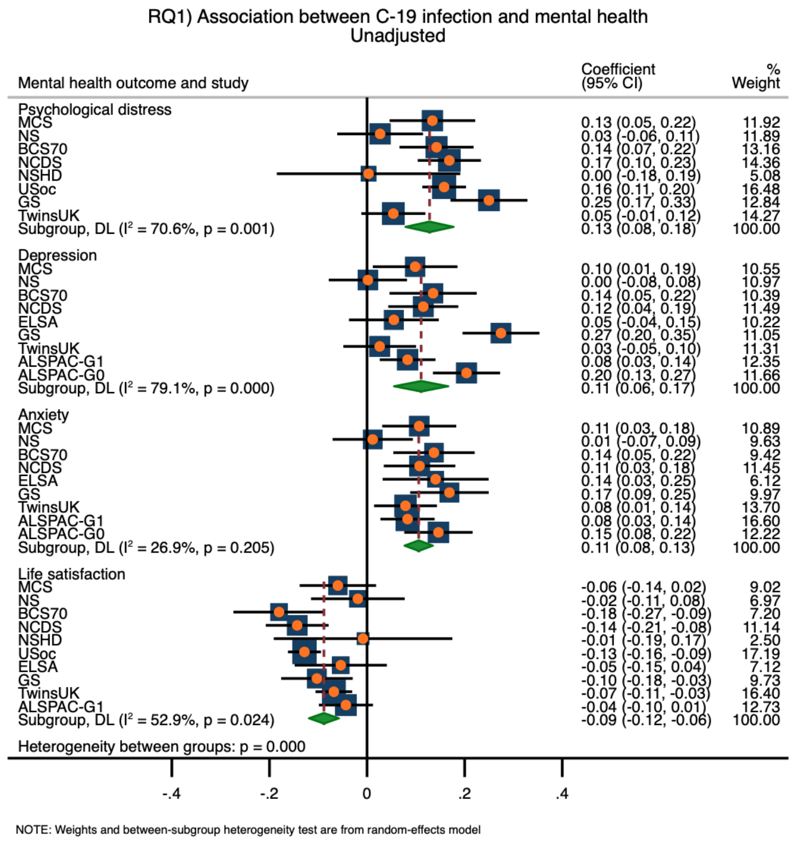

Figure S2. RQ1) Association between COVID-19 infection and mental health (unadjusted; binary outcome)

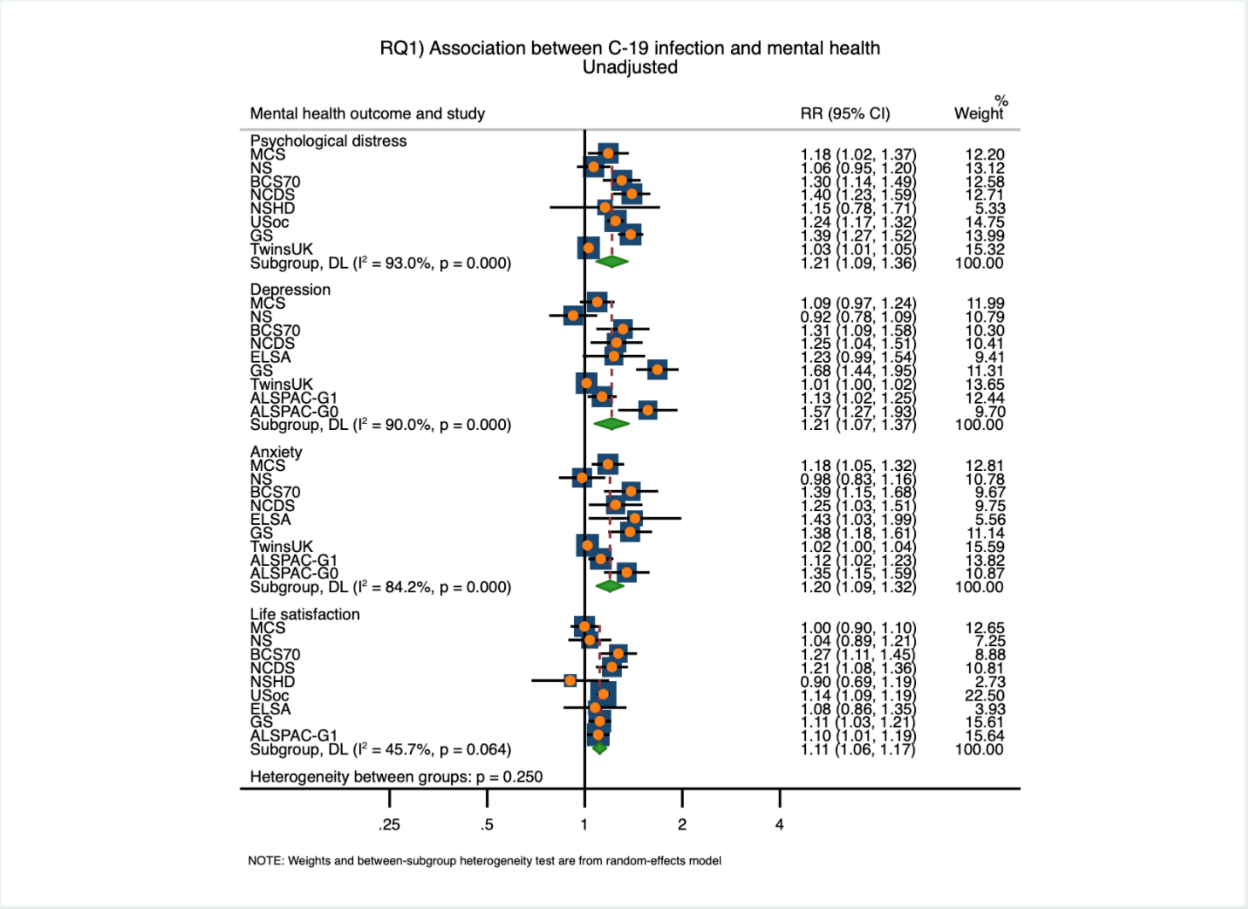

Figure S3. RQ2) Time since COVID-19 and psychological distress (adjusted; binary outcome)

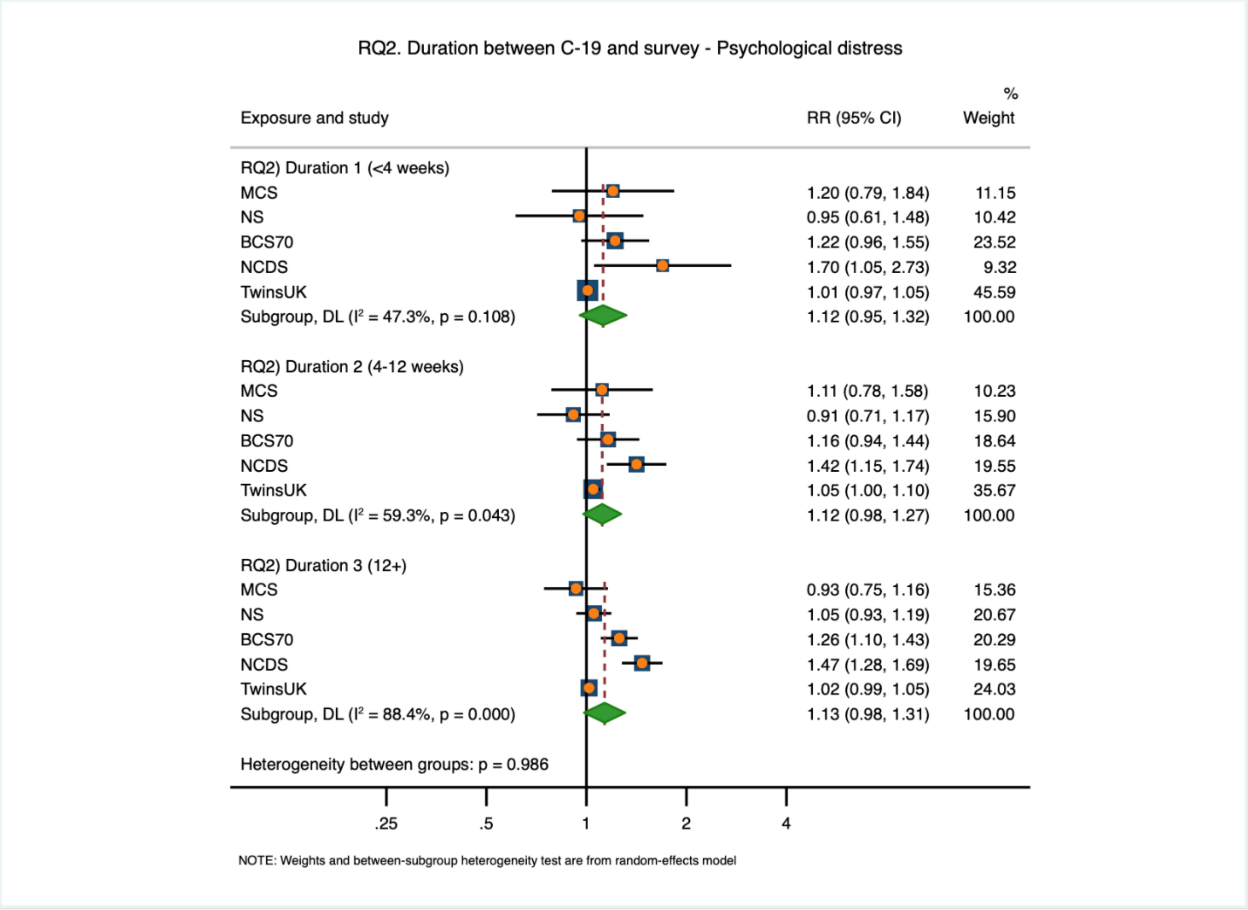

Figure S4. RQ2) Time since COVID-19 and depression (adjusted; binary outcome)

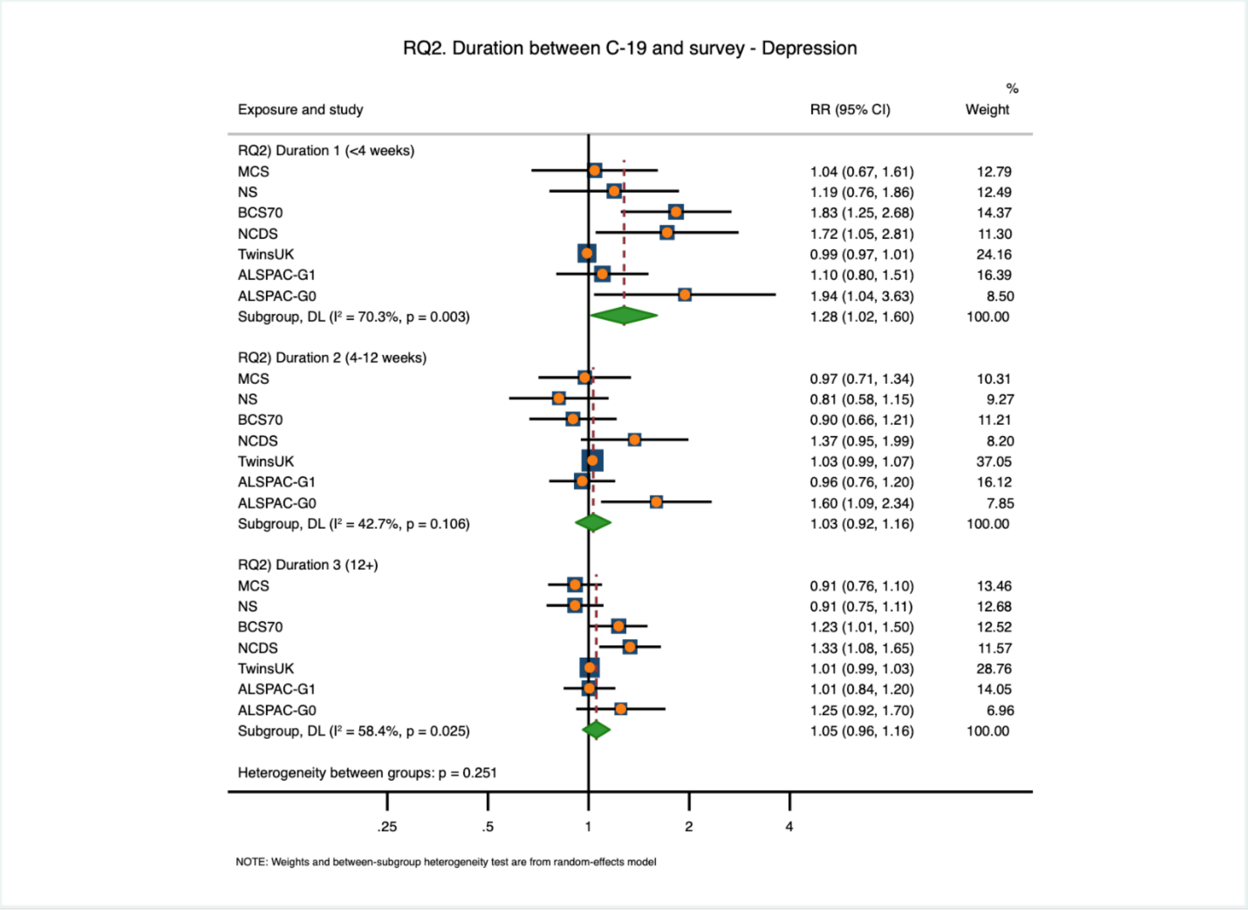

Figure S5. RQ2) Time since COVID-19 and anxiety (adjusted; binary outcome)

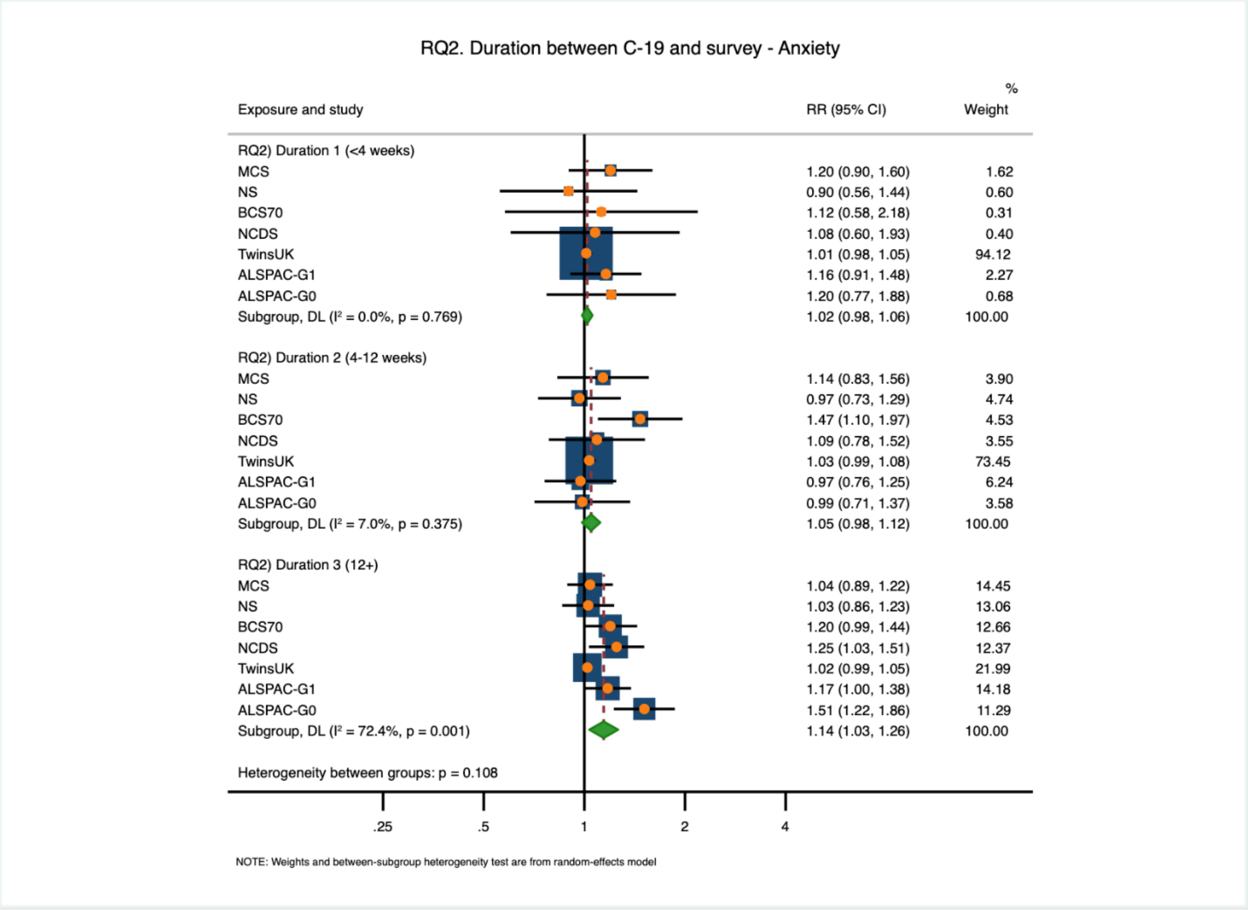

Figure S6. RQ2) Time since COVID-19 and low life satisfaction (adjusted; binary outcome)

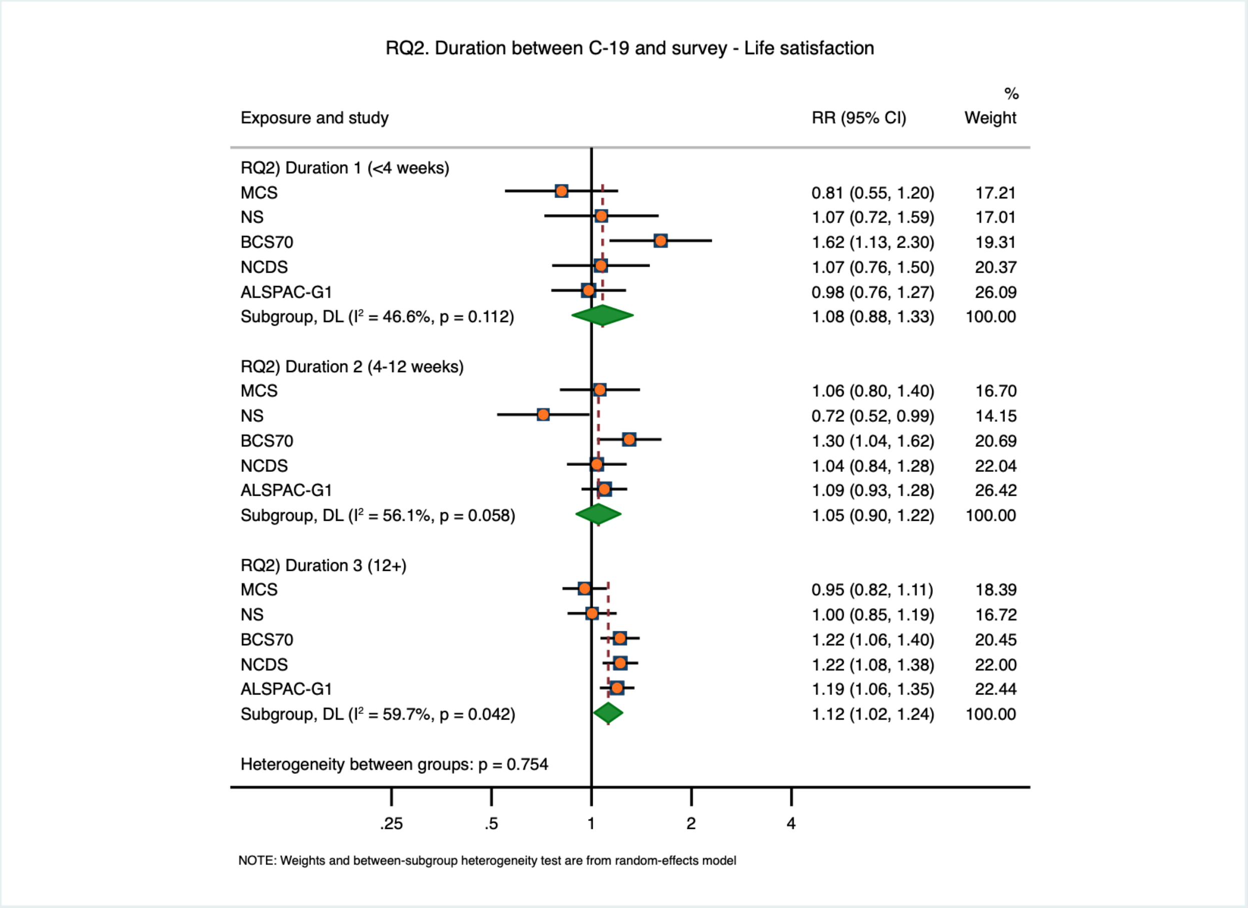

Figure S7. RQ3) Interactions with COVID-19 - psychological distress (adjusted; continuous outcome)

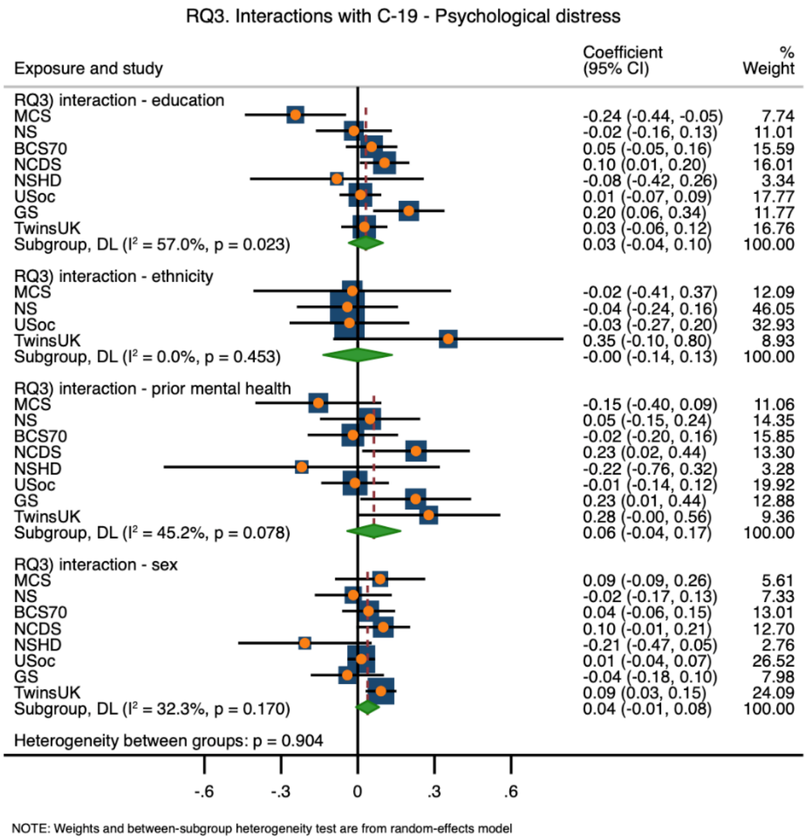

Figure S8. RQ3) Interactions with COVID-19 - depression (adjusted; continuous outcomes)

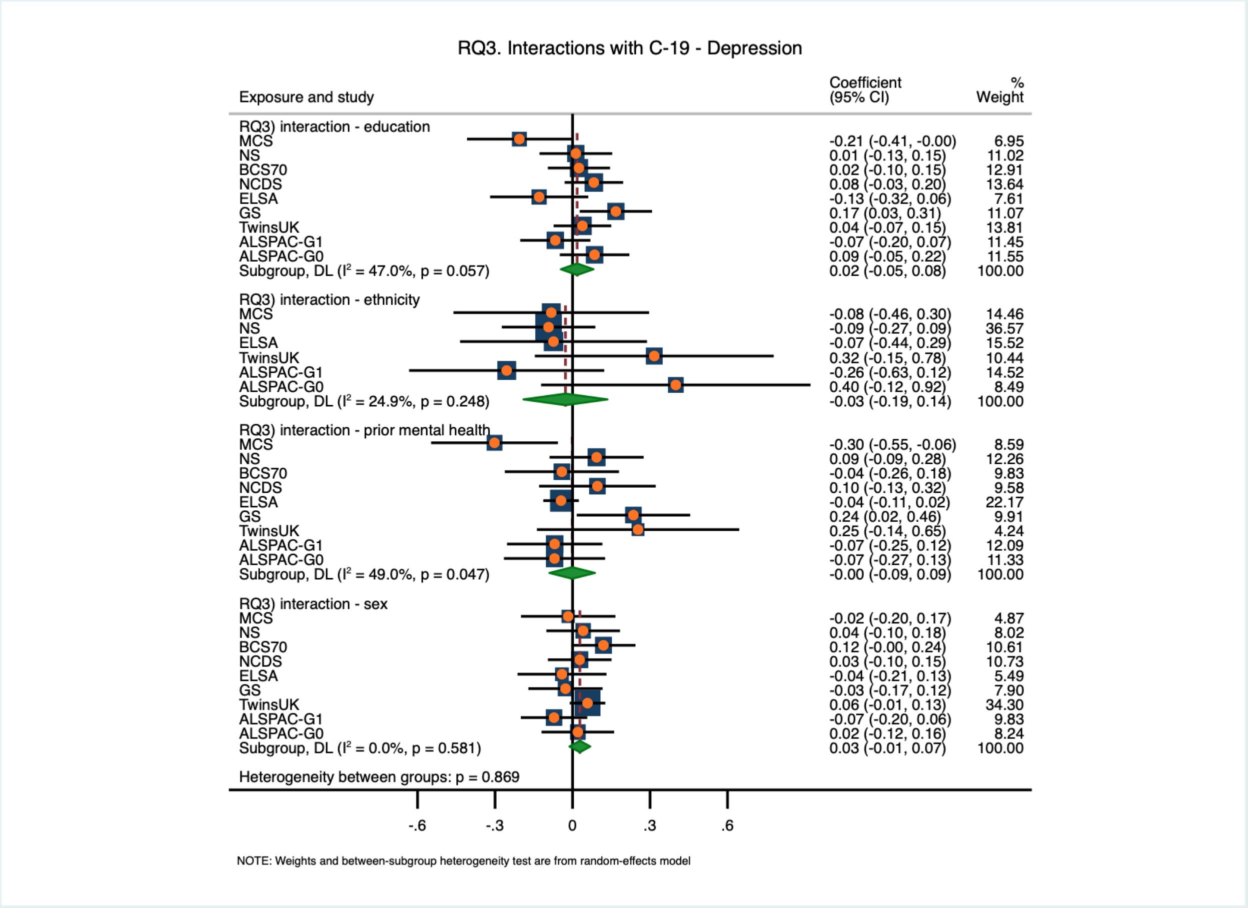

Figure S9. RQ3) Interactions with COVID-19 - anxiety (adjusted; continuous outcomes)

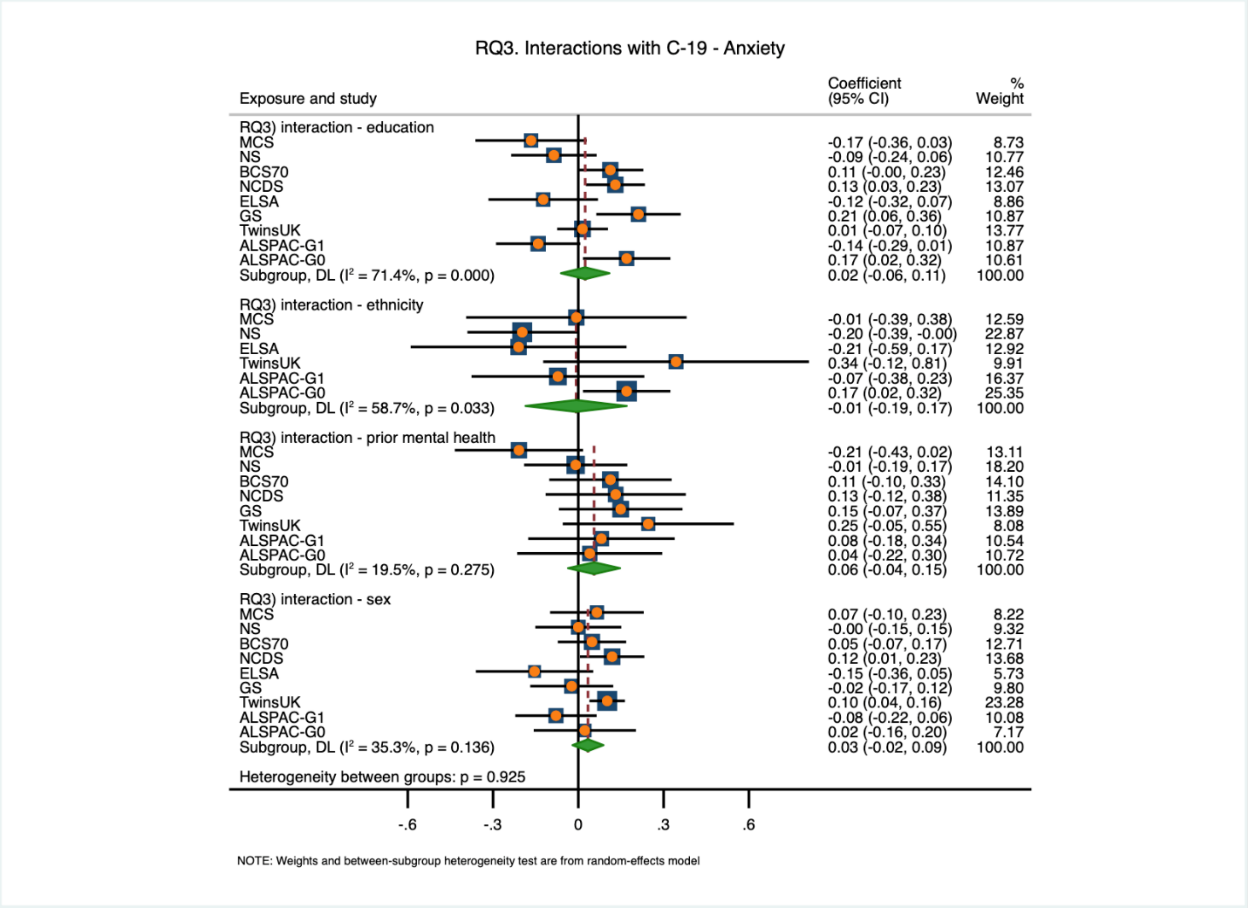

Figure S10. RQ3) Interactions with COVID-19 - life satisfaction (adjusted; continuous outcomes)

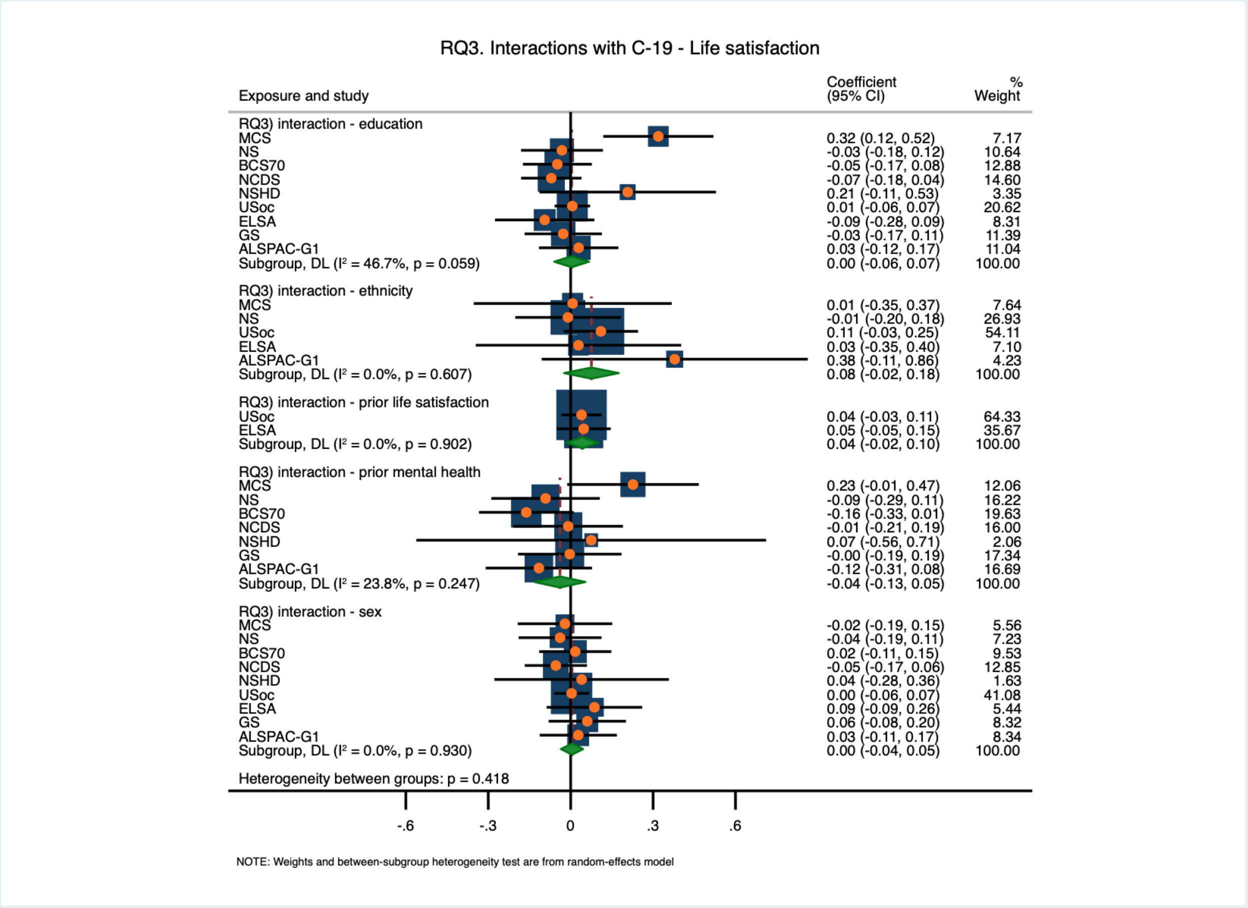

Figure S11. RQ3) Age stratified association between COVID-19 and psychological distress (adjusted; continuous outcomes)

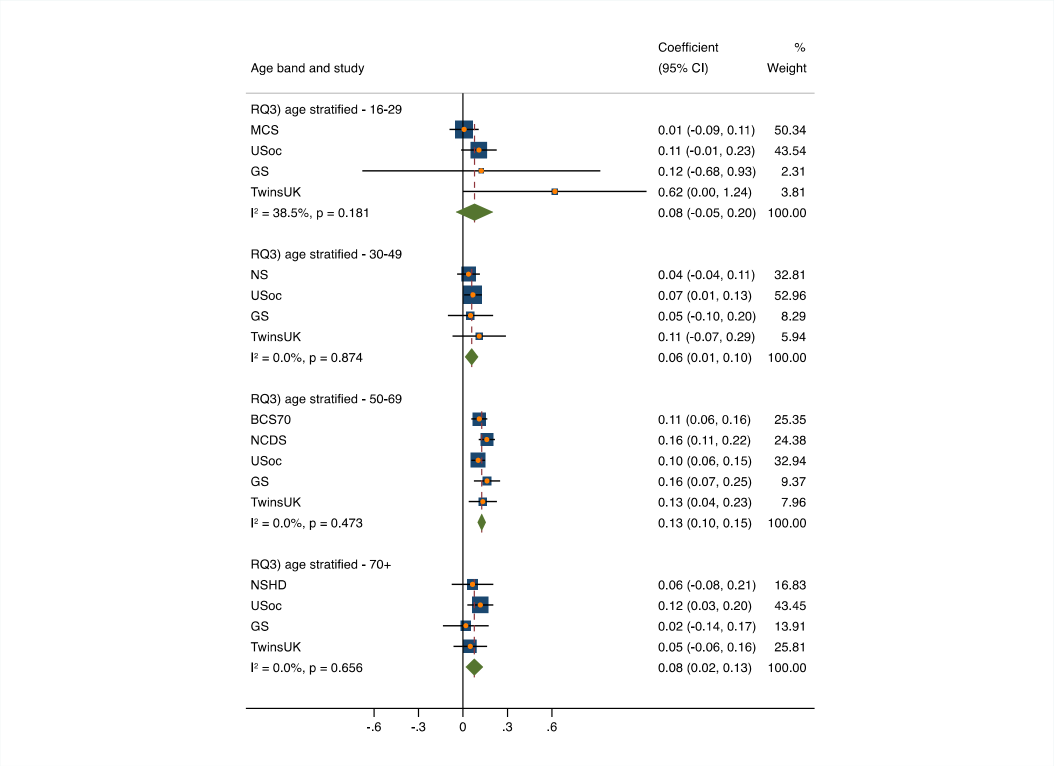

Figure S12. RQ3) Age stratified association between COVID-19 and depression (adjusted; continuous outcomes)

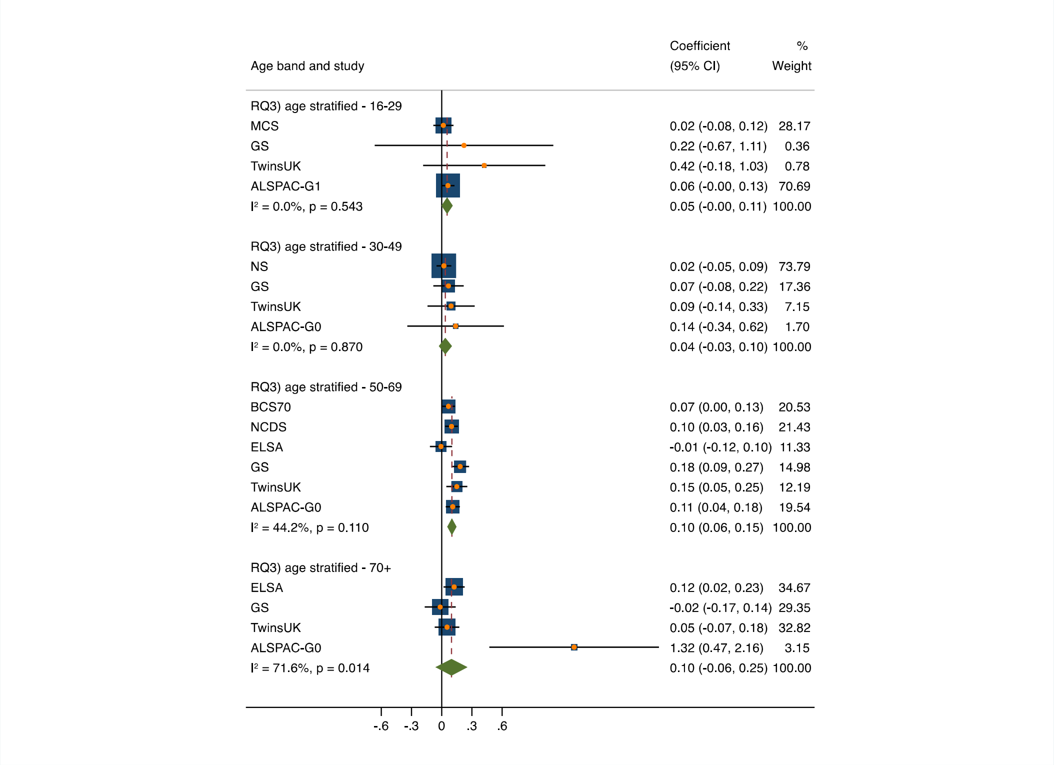

Figure S13. RQ3) Age stratified association between COVID-19 and anxiety (adjusted; continuous outcomes)

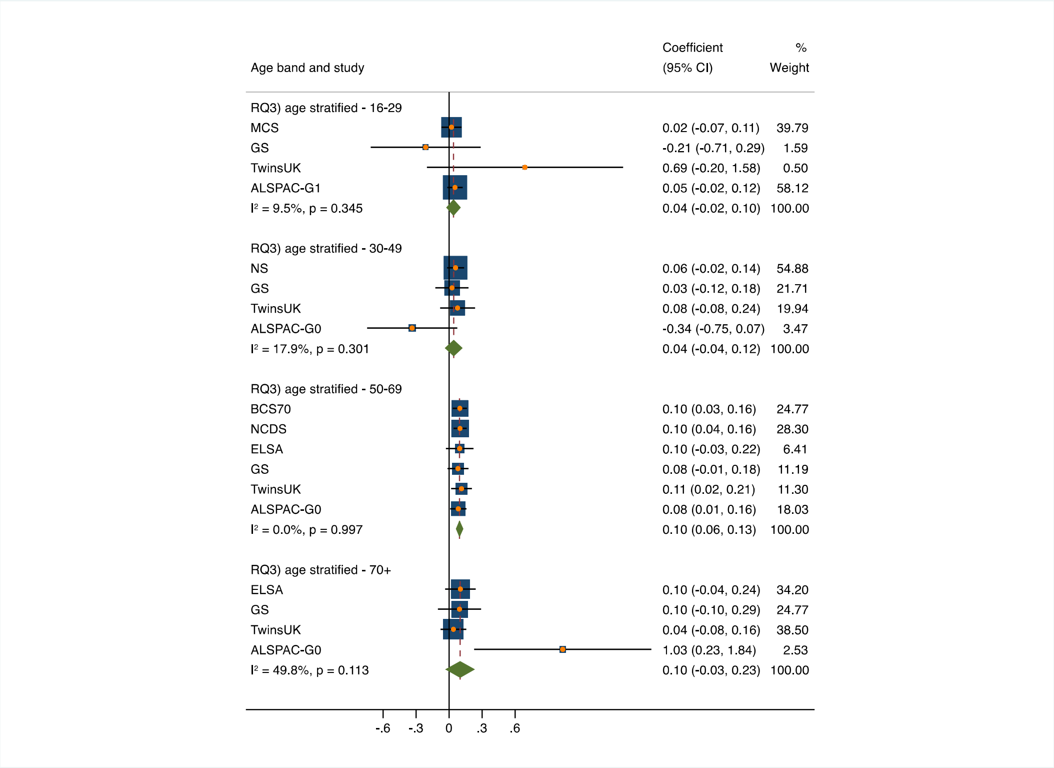

Figure S14. RQ3) Age stratified association between COVID-19 and life satisfaction (adjusted; continuous outcomes)

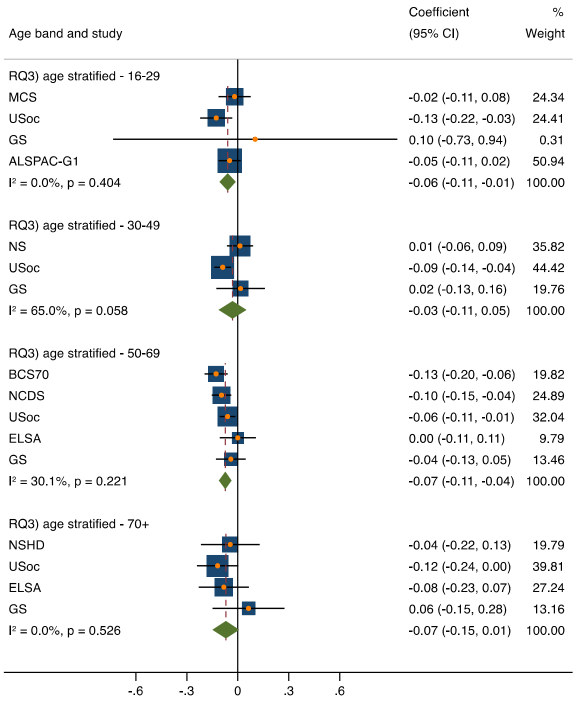

Figure S15. RQ4A) Test-confirmed vs suspected COVID-19 and psychological distress (adjusted; binary outcome)

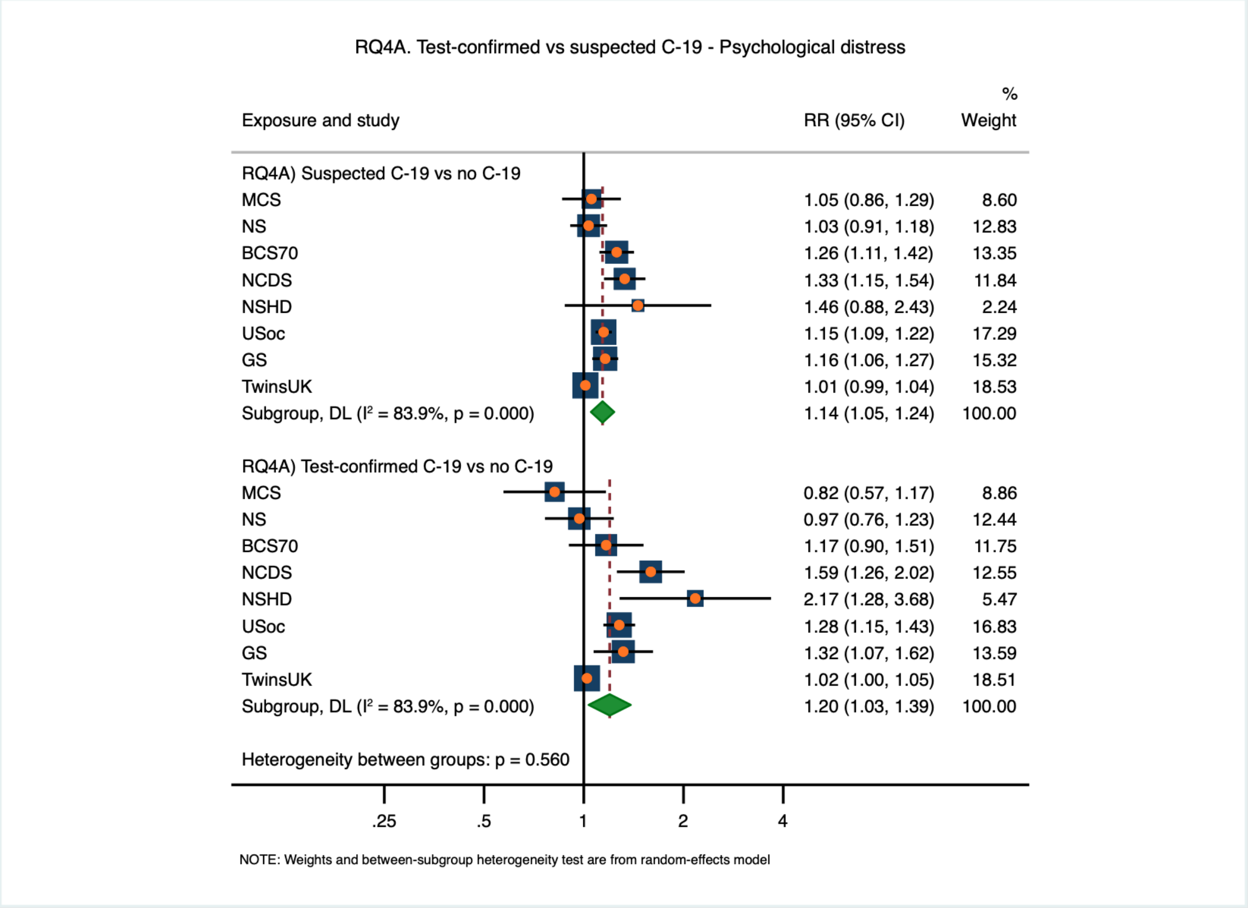

Figure S16. RQ4A) Test-confirmed vs suspected COVID-19 and depression (adjusted; binary outcome)

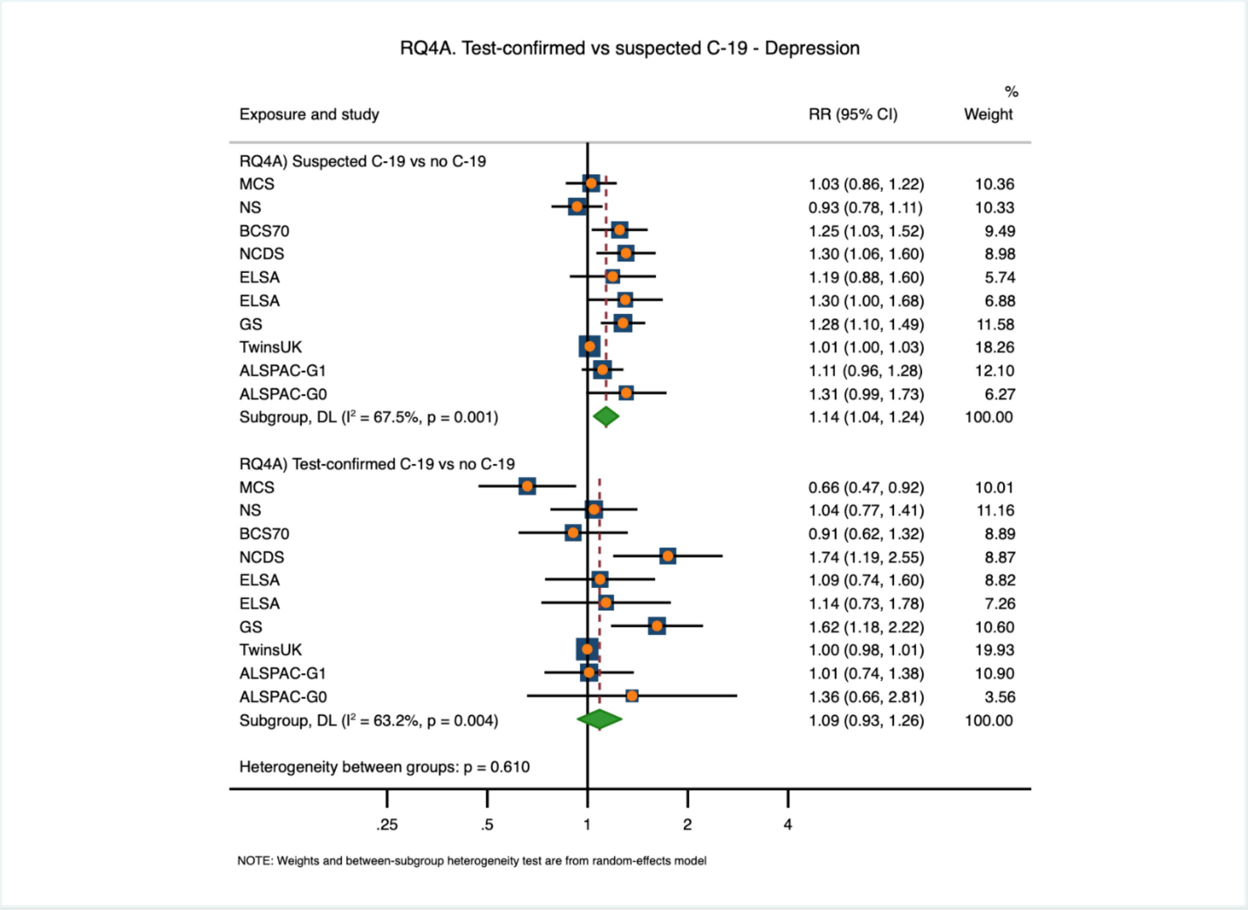

Figure S17. RQ4A) Test-confirmed vs suspected COVID-19 and anxiety (adjusted; binary outcome)

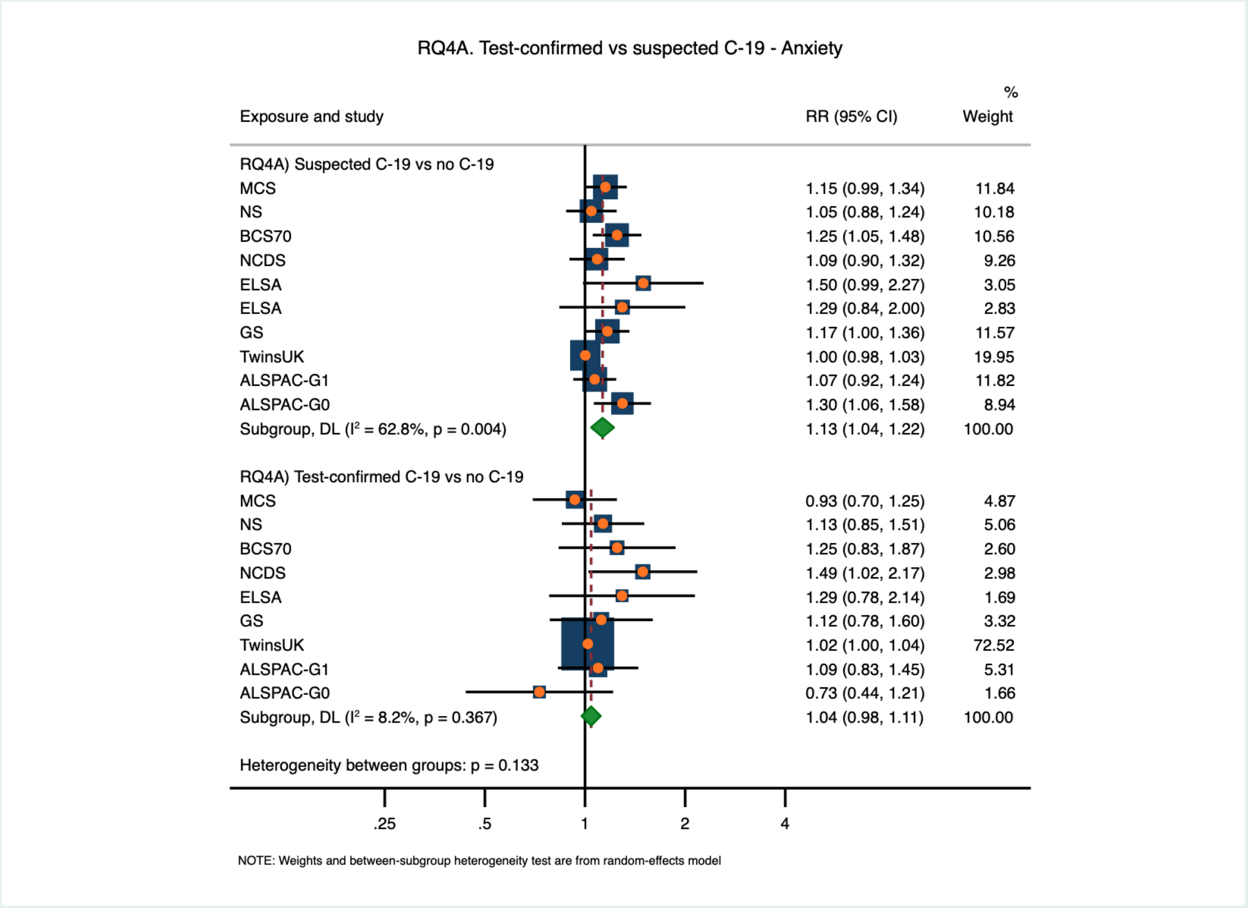

Figure S18. RQ4A) Test-confirmed vs suspected COVID-19 and low life satisfaction (adjusted; binary outcome)

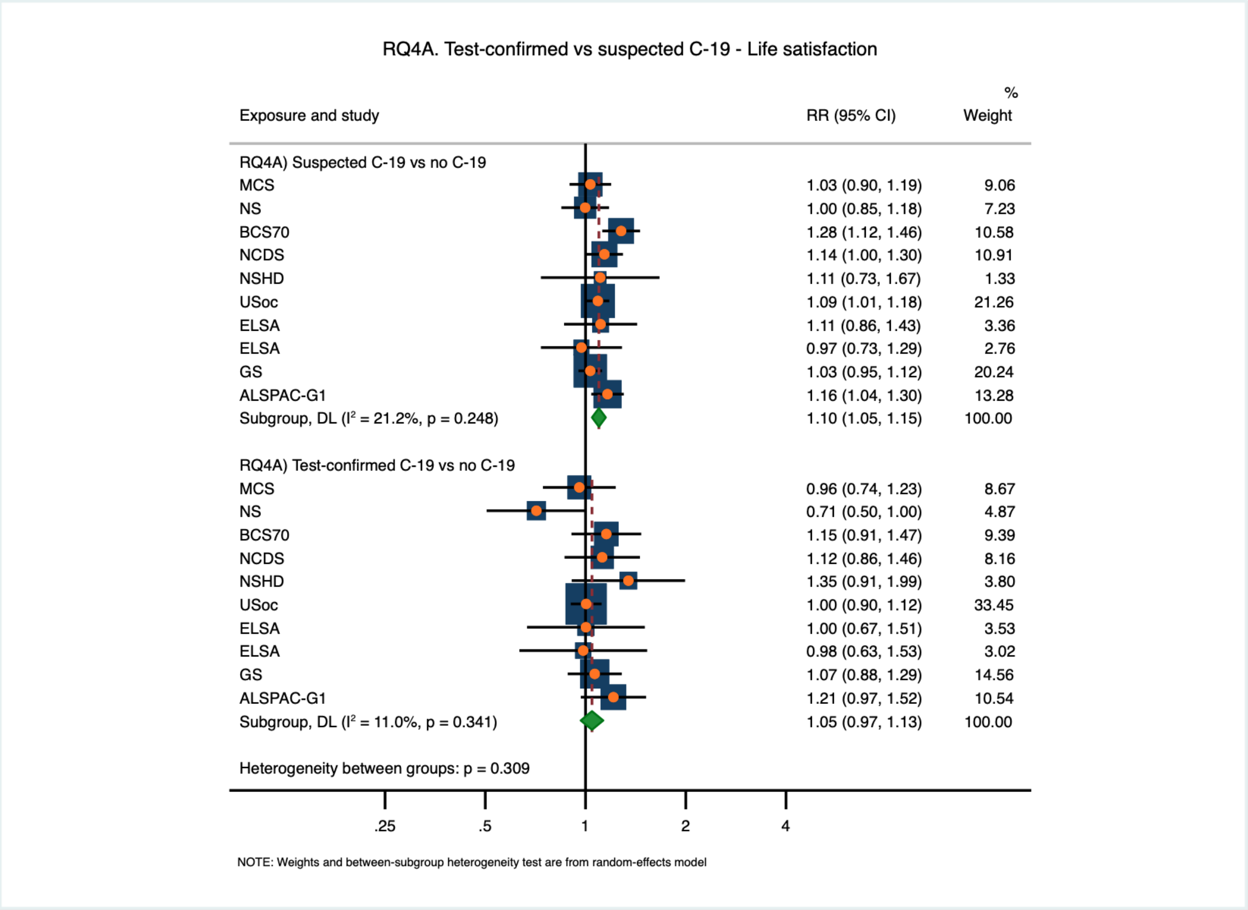

Figure S19. RQ4B) Serology and self-report - psychological distress (adjusted; binary outcome)

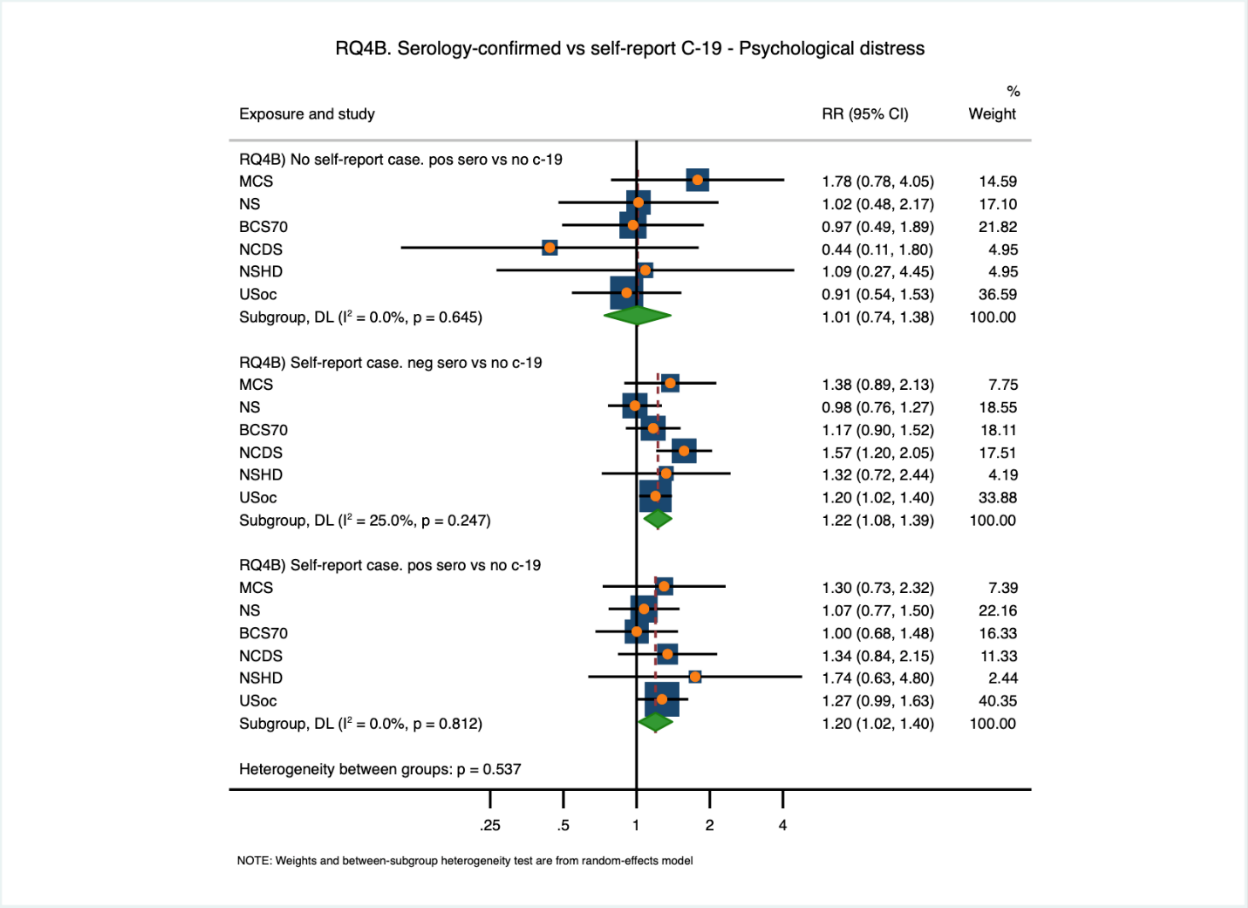

Figure S20. RQ4B) Serology and self-report - depression (adjusted; binary outcome)

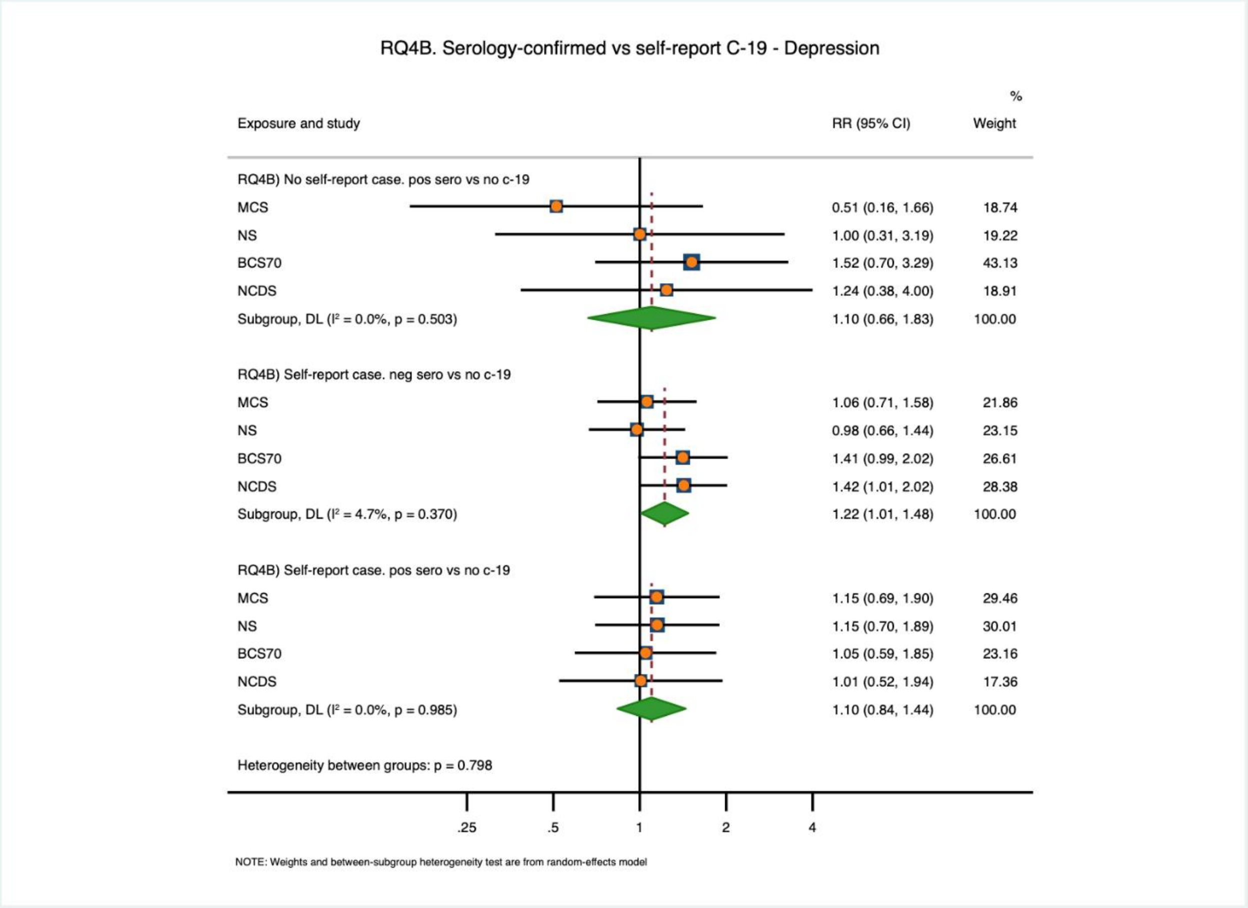

Figure S21. RQ4B) Serology and self-report - anxiety (adjusted; binary outcome)

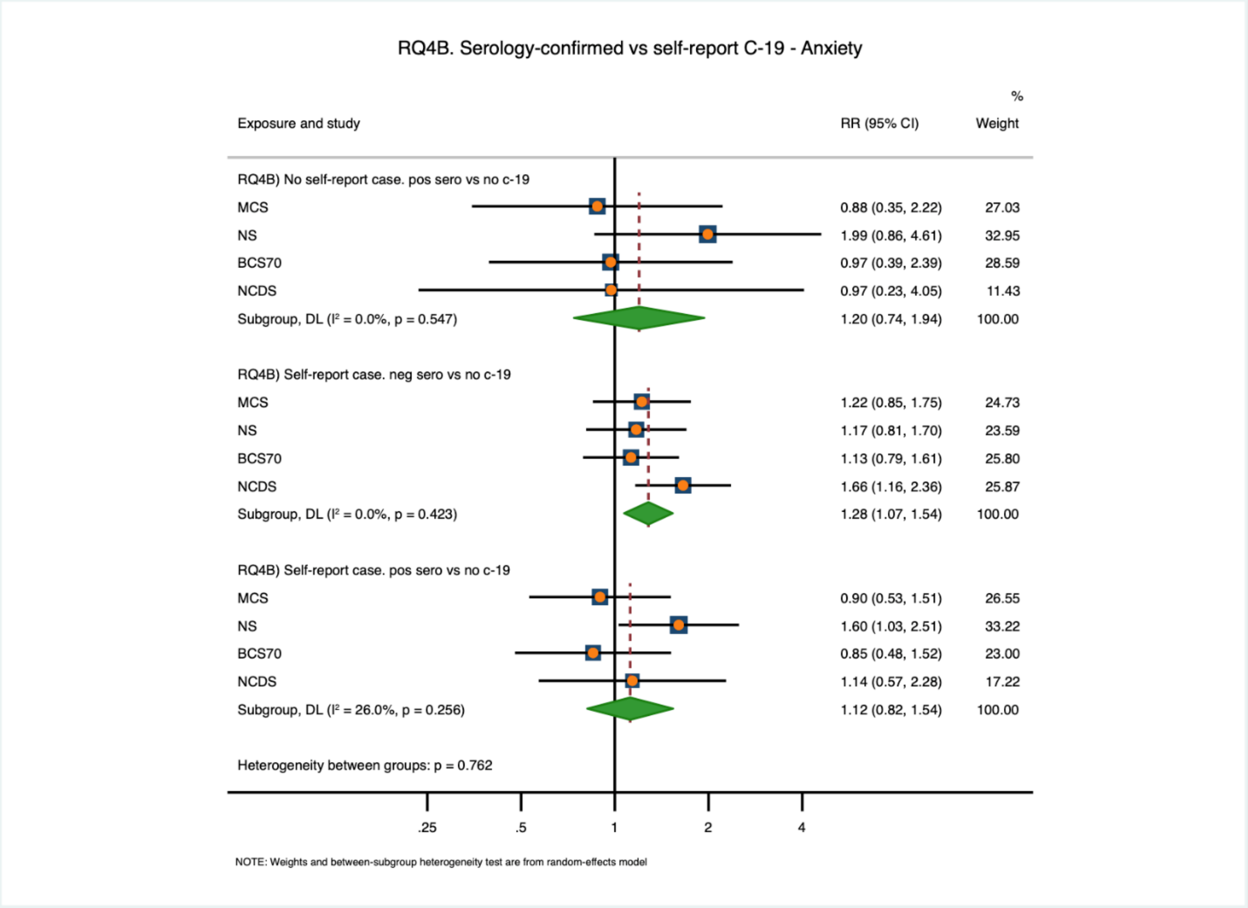

Figure S22. RQ4B) Serology and self-report - low life satisfaction (adjusted; binary outcome)

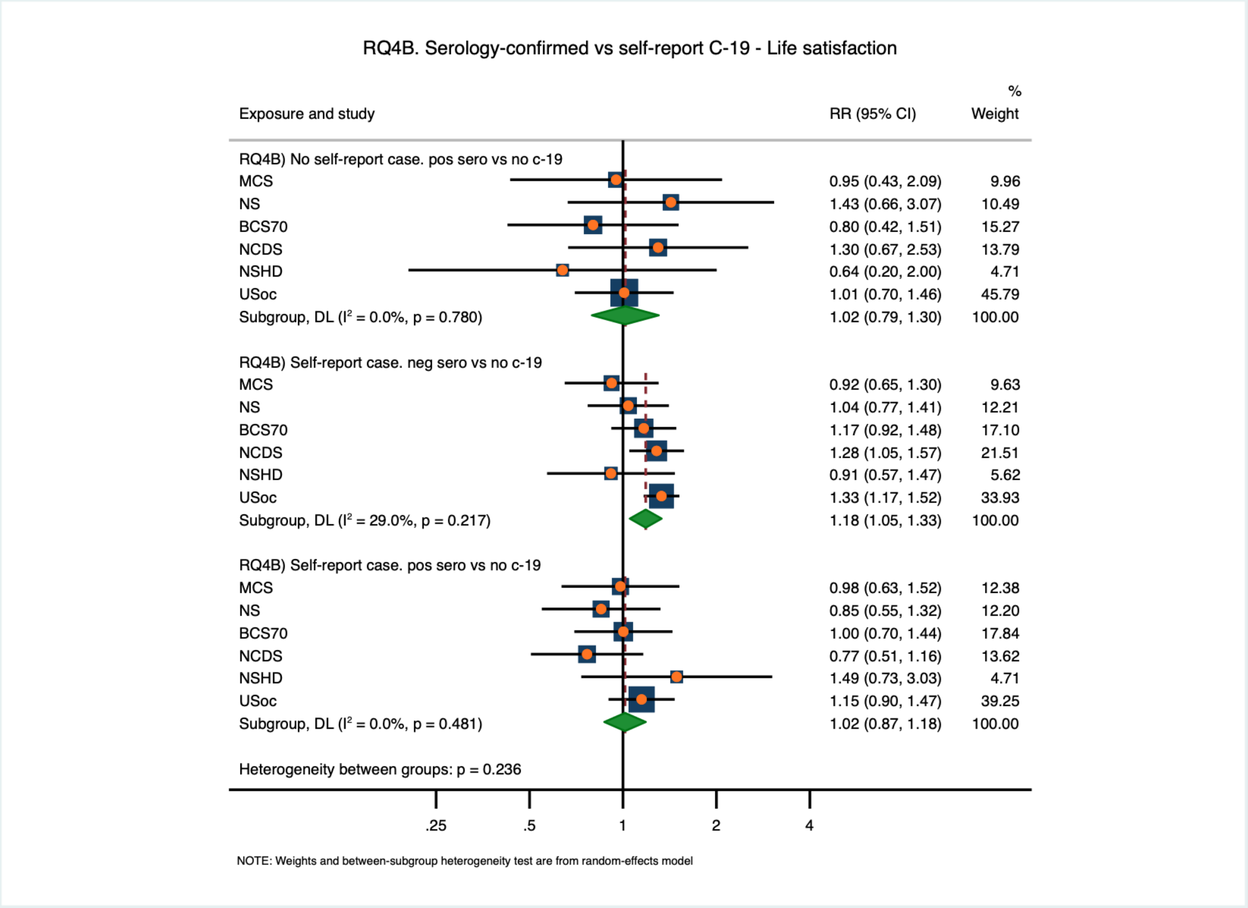

Figure S23. Exploratory analysis – binary serology exposure – positive serology vs negative serology (continuous outcomes)

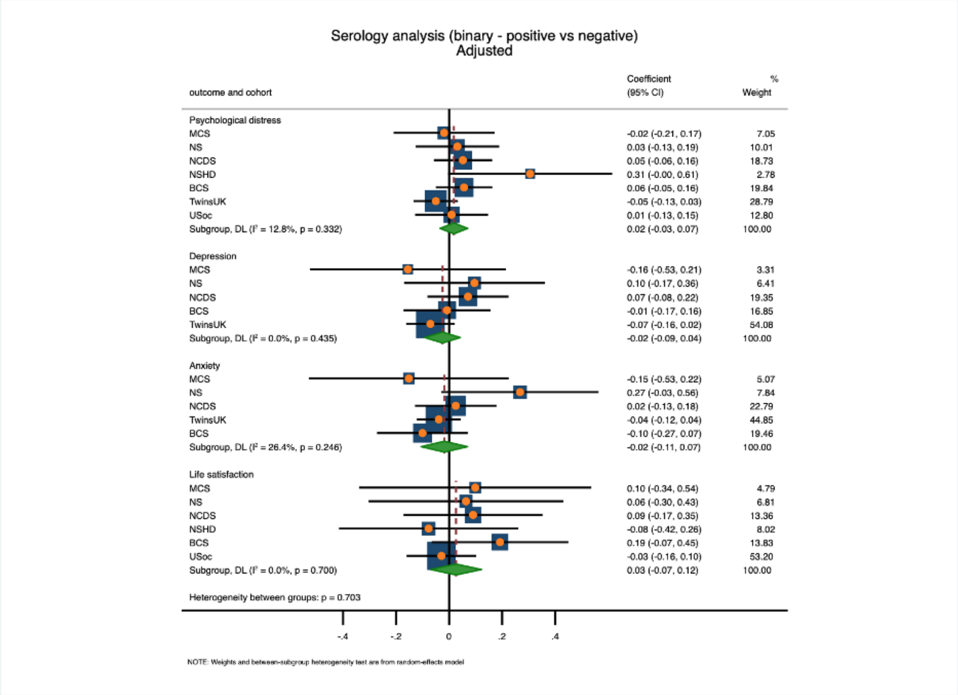

Figure S24. RQ1 restricted to MCS, NS, BCS70, NCDS and NSHD datasets (adjusted; continuous outcome)

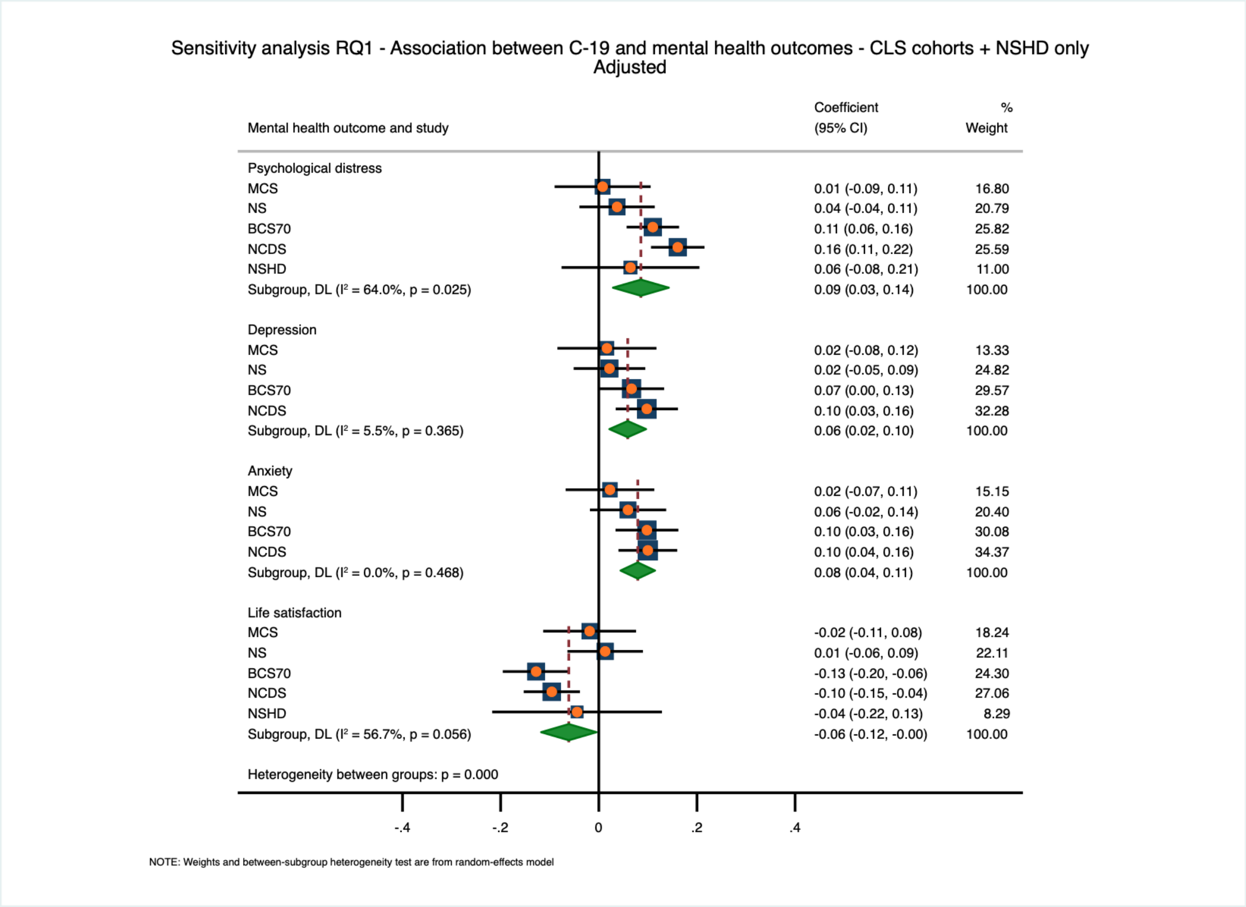

Figure S25. Sensitivity analysis 1 - Unsure COVID-19 grouped as 'no COVID-19' (adjusted; continuous outcome)

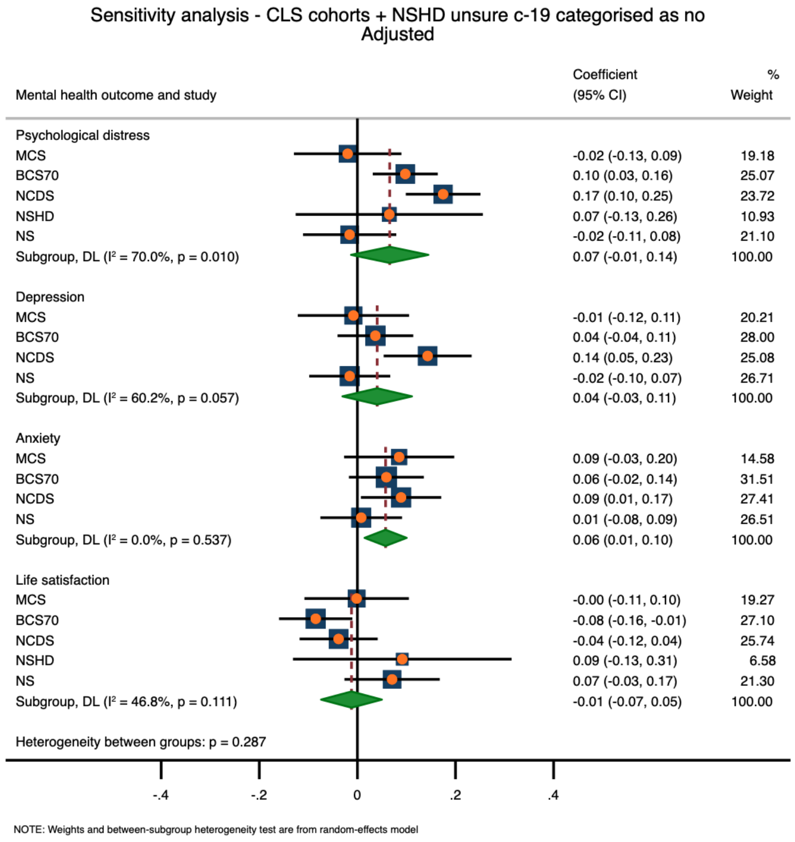

Figure S26. Sensitivity analysis 2 - Unsure COVID-19 separate - psychological distress (adjusted; continuous outcome)

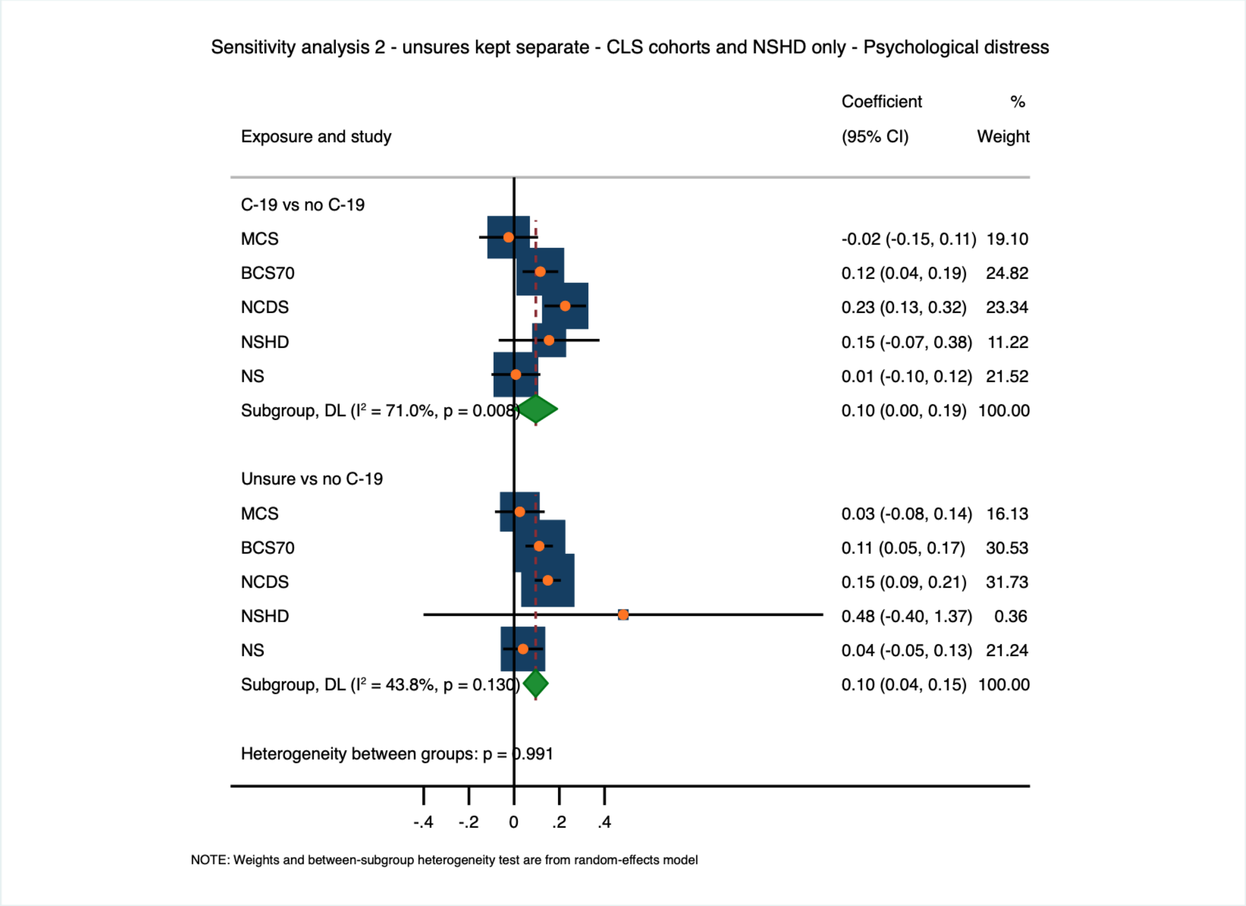

Figure S27. Sensitivity analysis 2 - Unsure COVID-19 separate - depression (adjusted; continuous outcome)

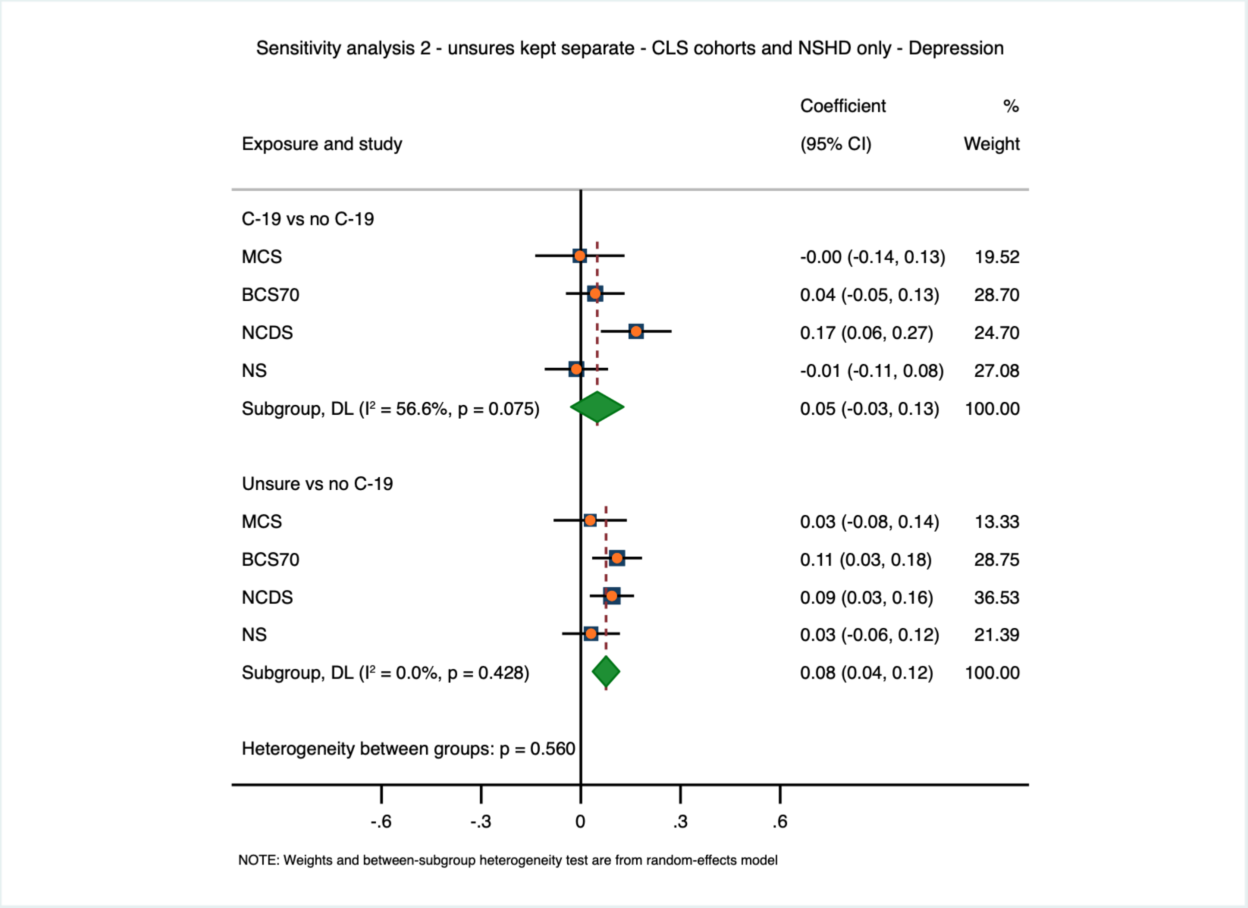

Figure S28. Sensitivity analysis 2 - Unsure C-19 separate - anxiety (adjusted; continuous outcome)

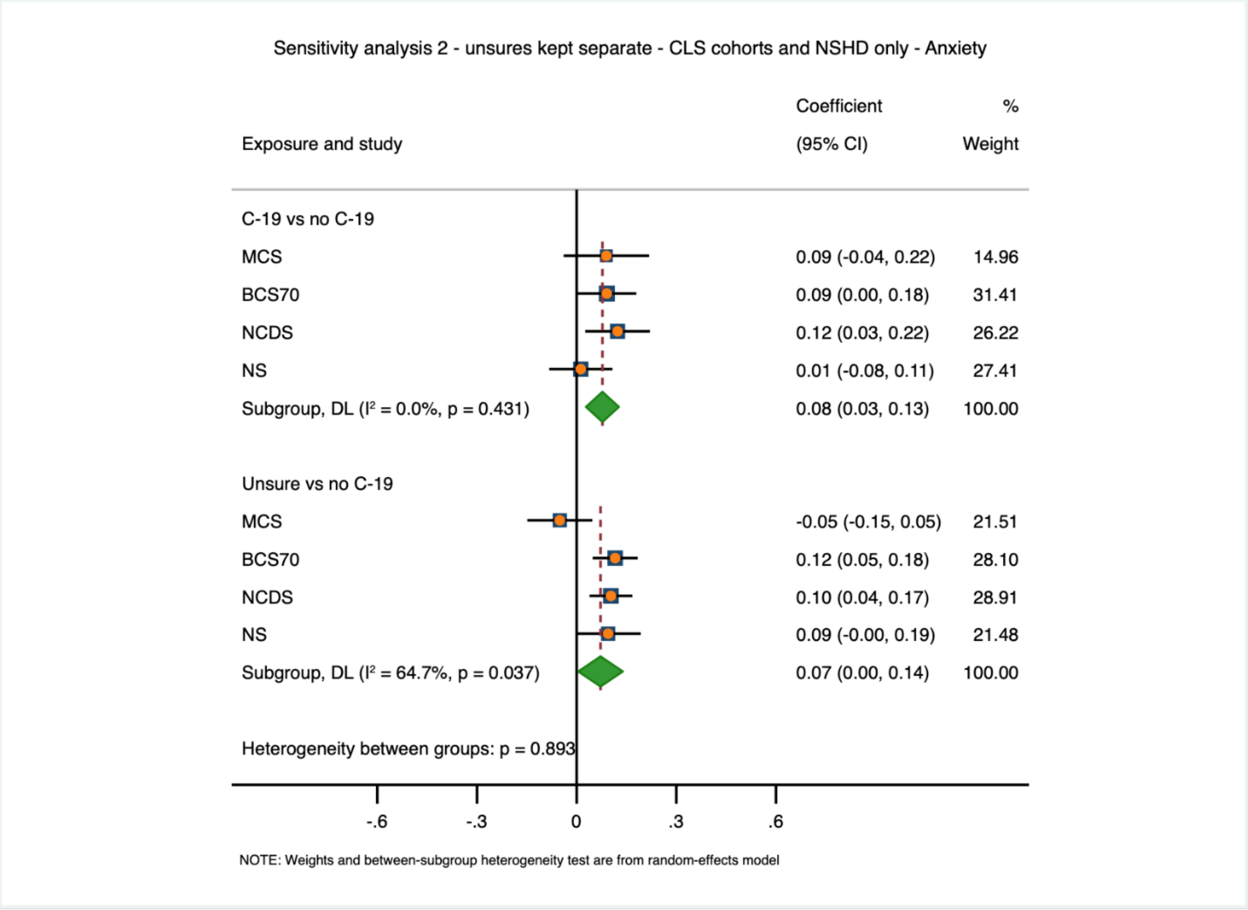

Figure S29. Sensitivity analysis 2 - Unsure COVID-19 separate - life satisfaction (adjusted; continuous outcome)

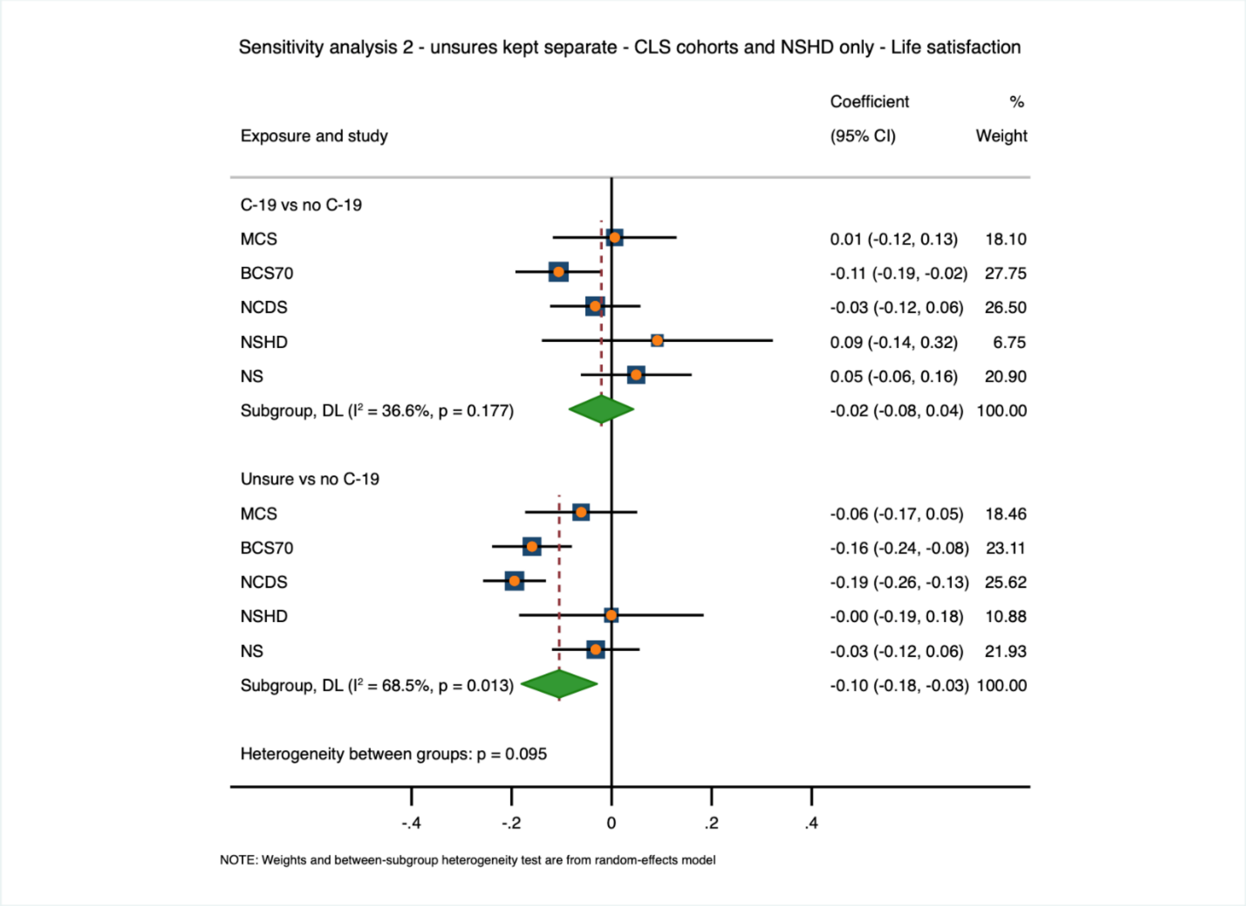

Figure S30. Time since COVID-19 and psychological distress (<4 weeks since infection as reference group)

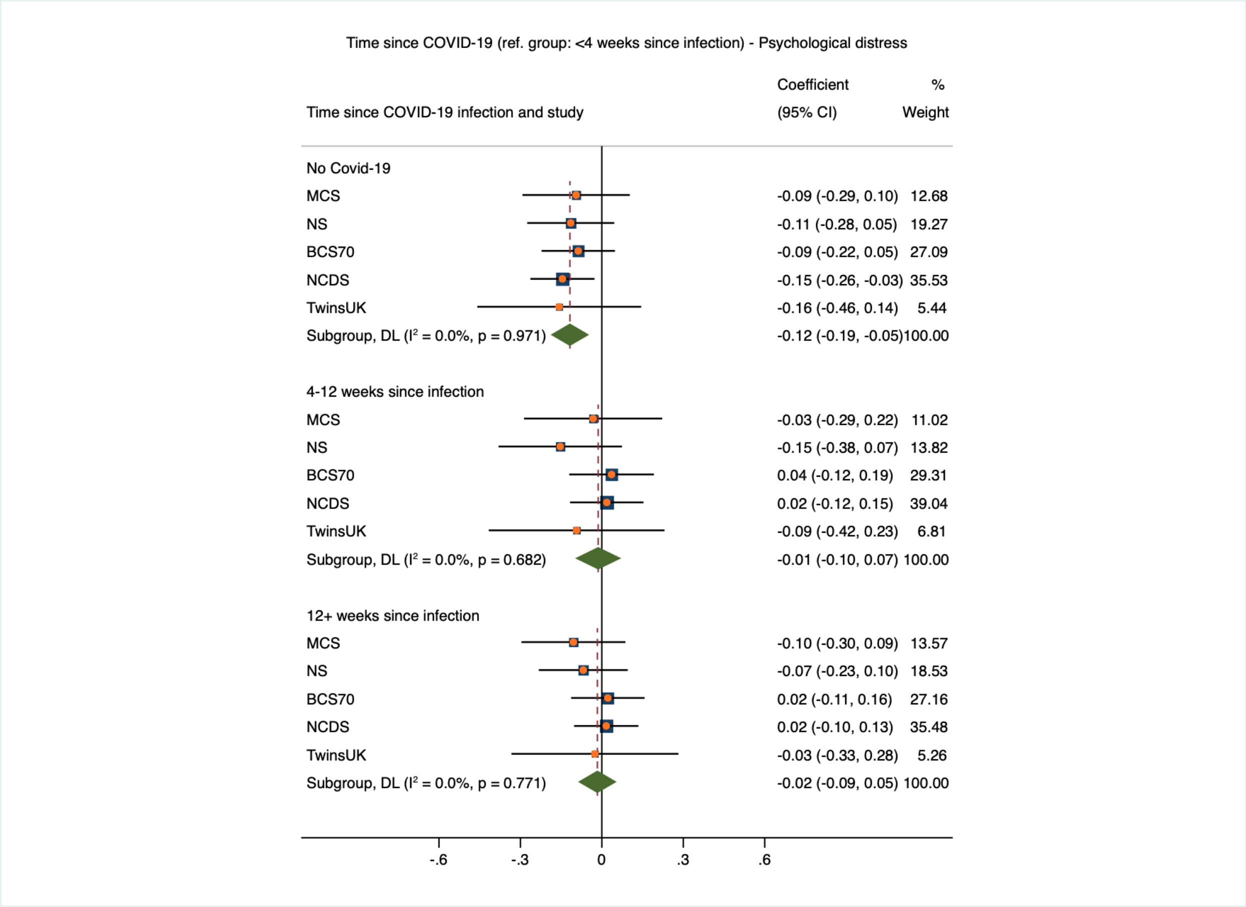

Figure S31. Time since COVID-19 and depression (<4 weeks since infection as reference group)

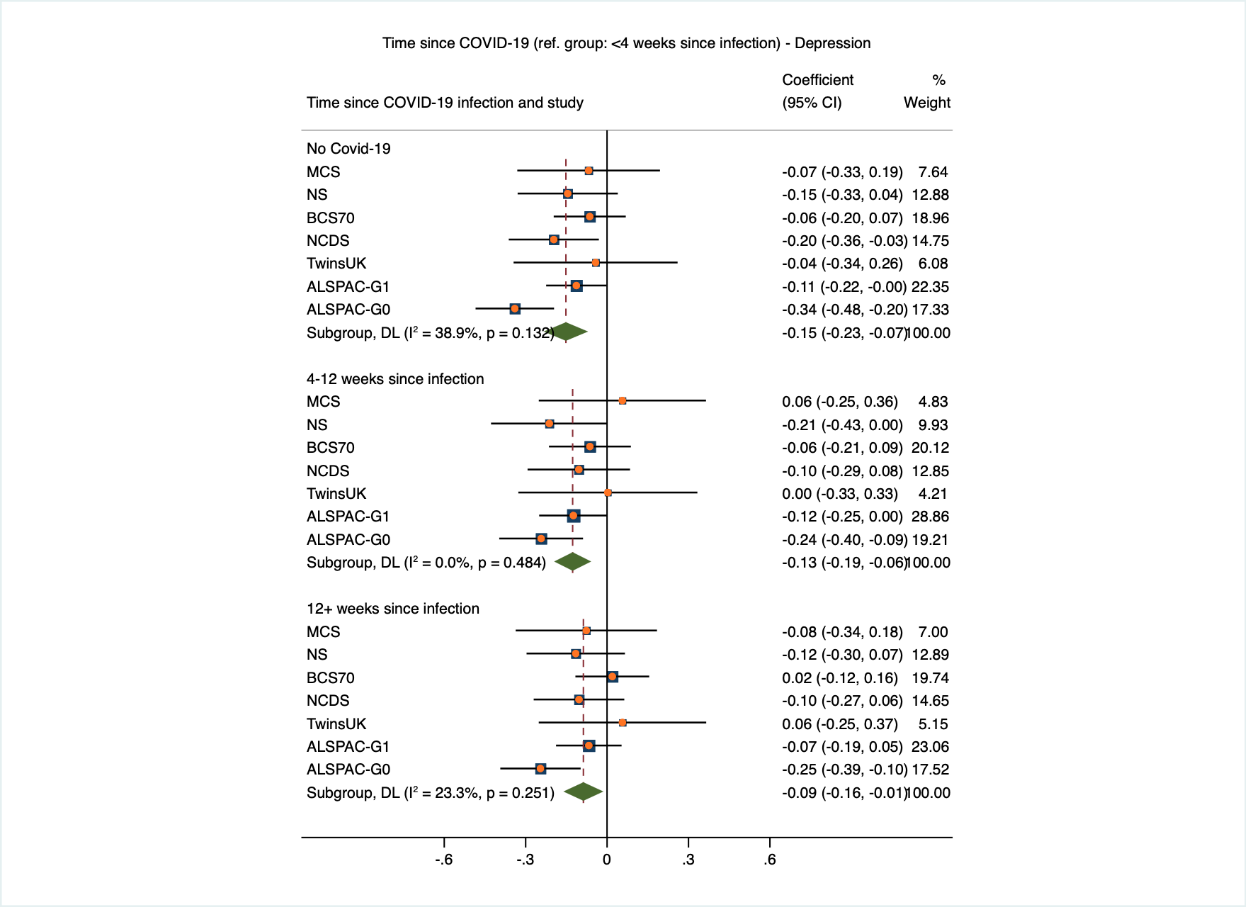

Figure S32. Time since COVID-19 and anxiety (<4 weeks since infection as reference group)

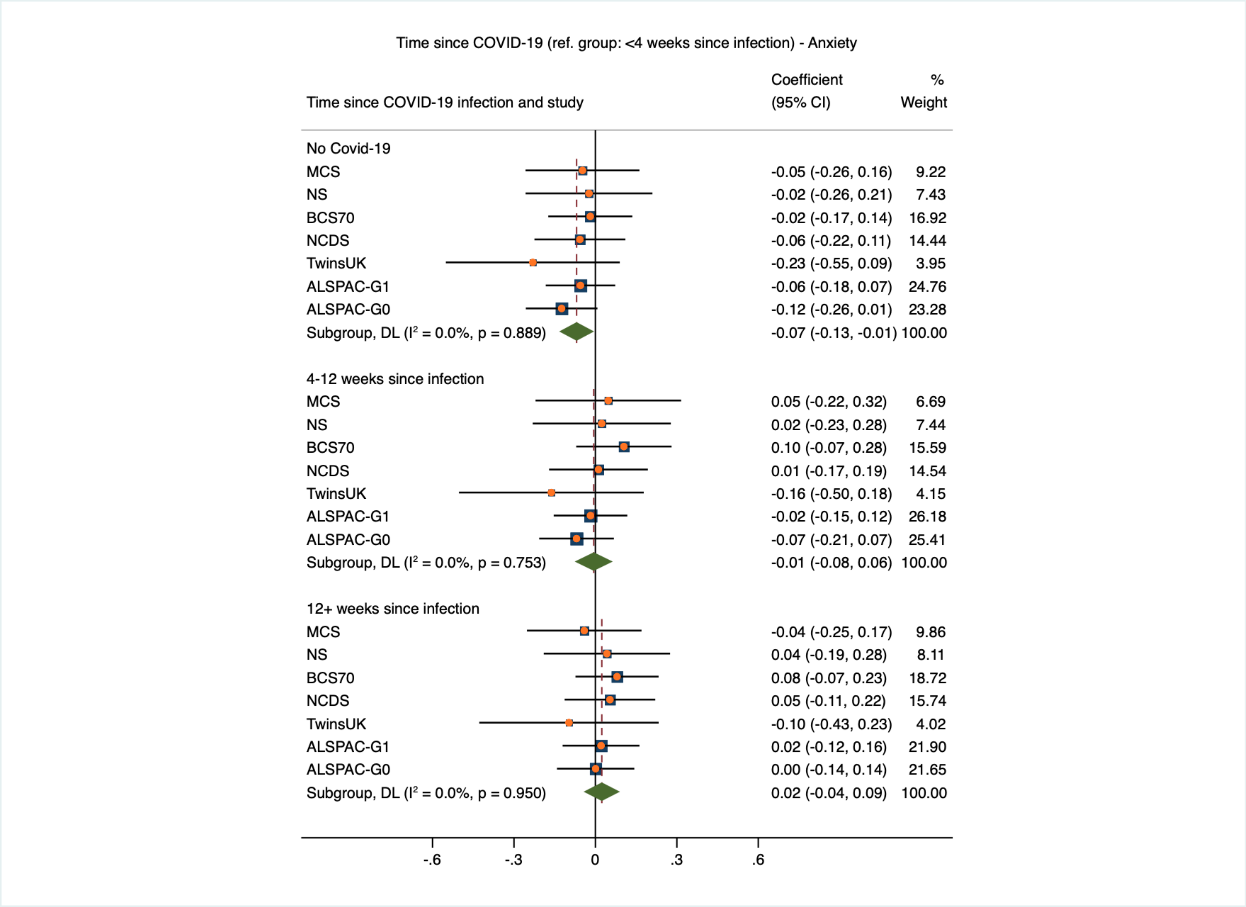

Figure S33. Time since COVID-19 and life satisfaction (<4 weeks since infection as reference group)

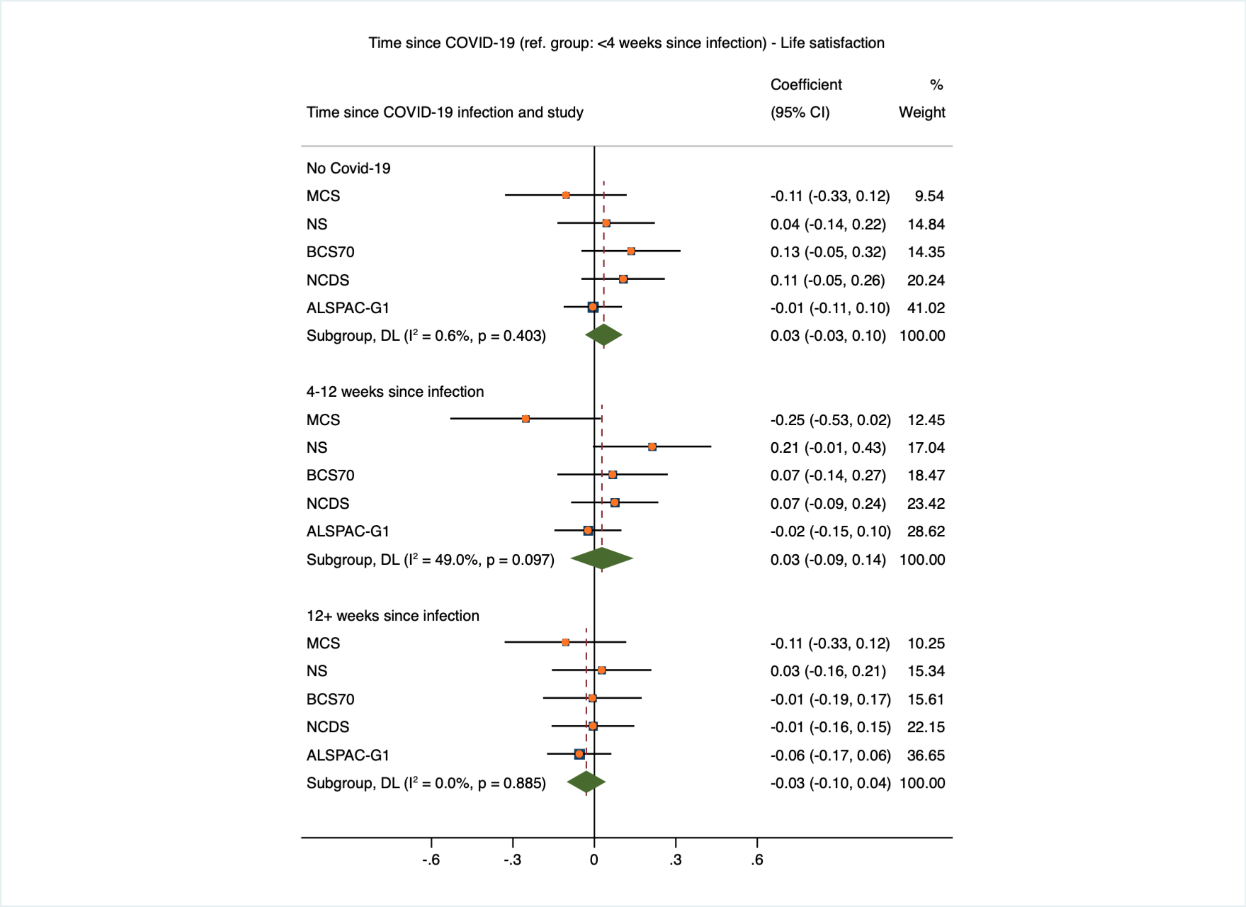

Figure S34. Test-confirmed vs suspected COVID-19 and psychological distress (test-confirmed as reference group)

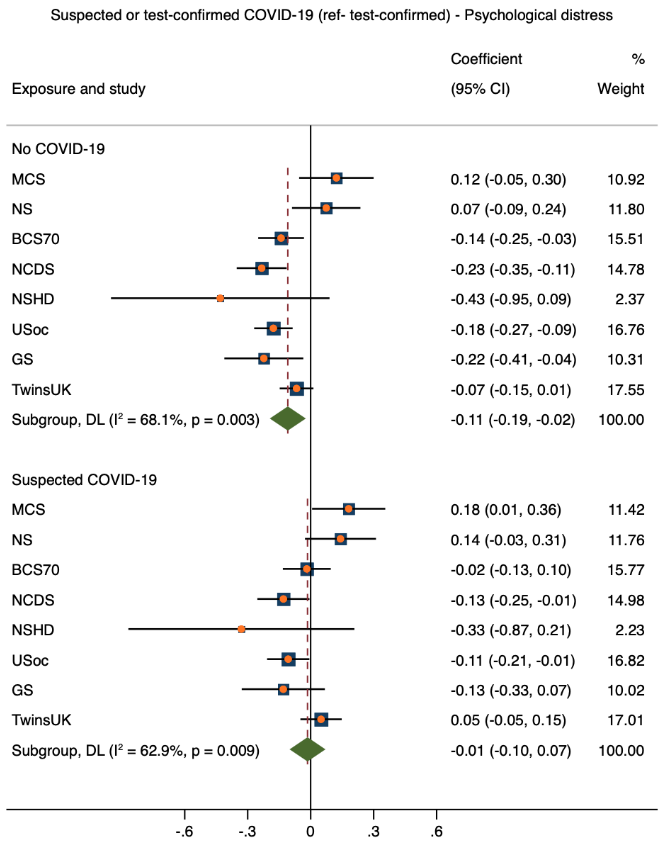

Figure S35. Test-confirmed vs suspected COVID-19 and depression (test-confirmed as reference group)

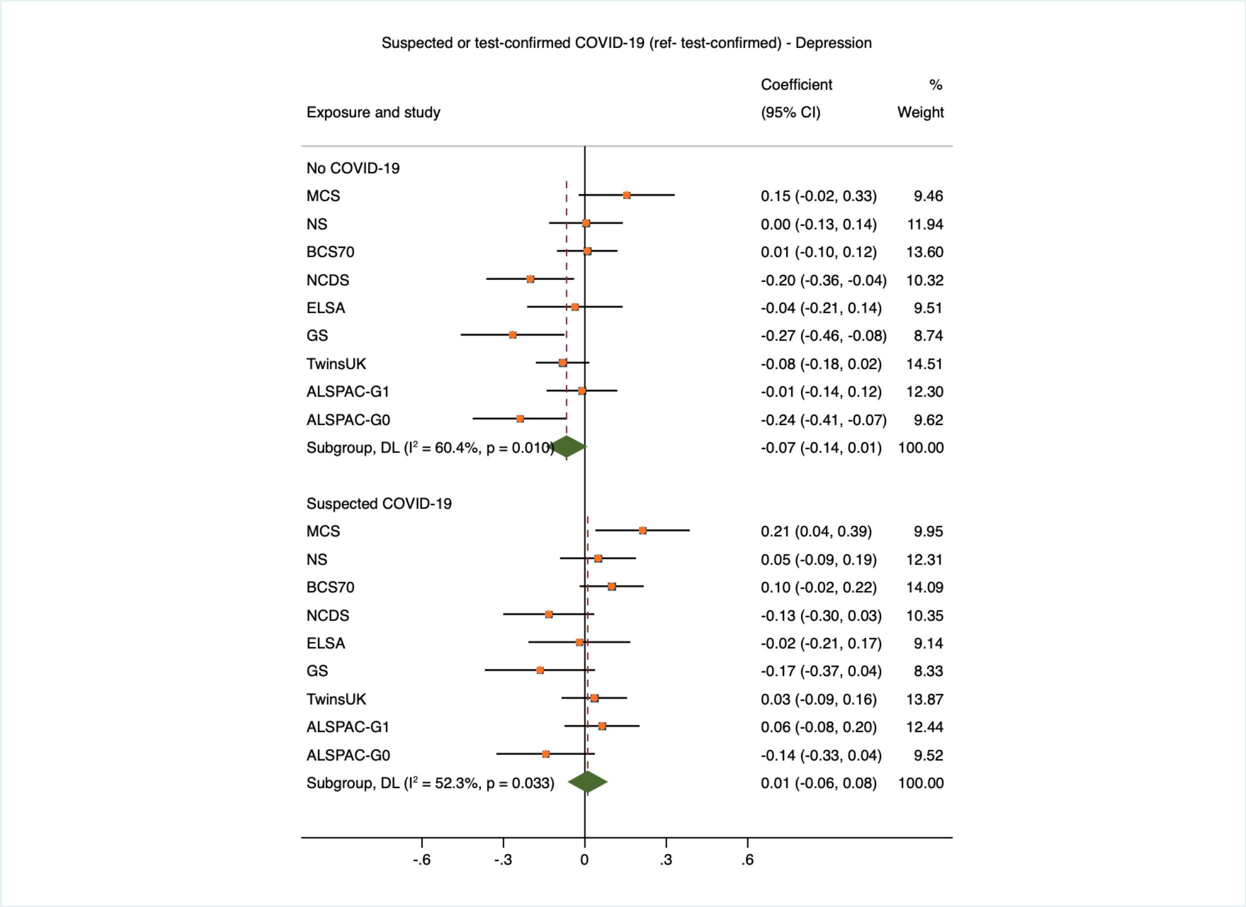

Figure S36. Test-confirmed vs suspected COVID-19 and anxiety (test-confirmed as reference group)

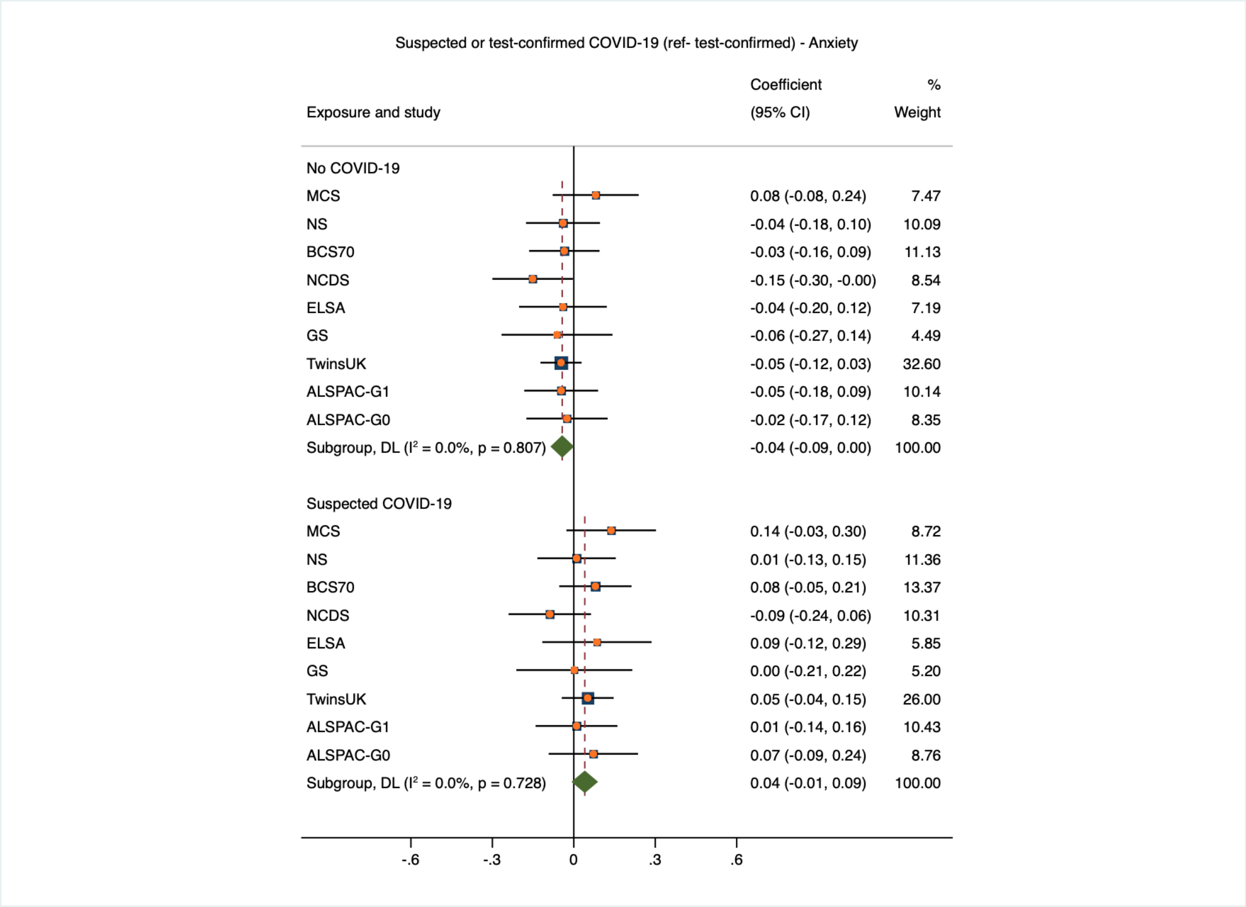

Figure S37. Test-confirmed vs suspected COVID-19 and life satisfaction (test-confirmed as reference group)

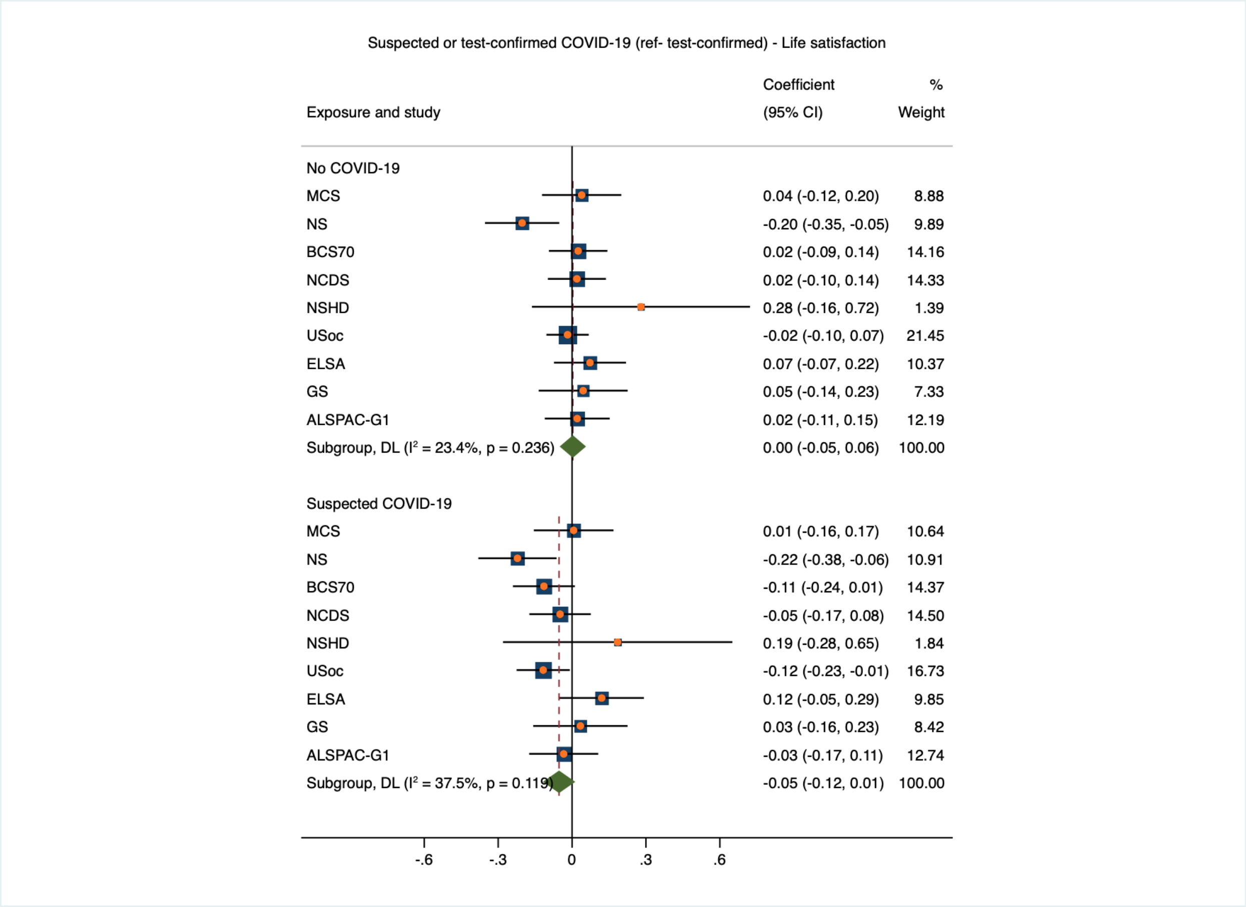

Figure S38. Self-report vs serology COVID-19 and psychological distress (positive self-report and positive serology as reference group)

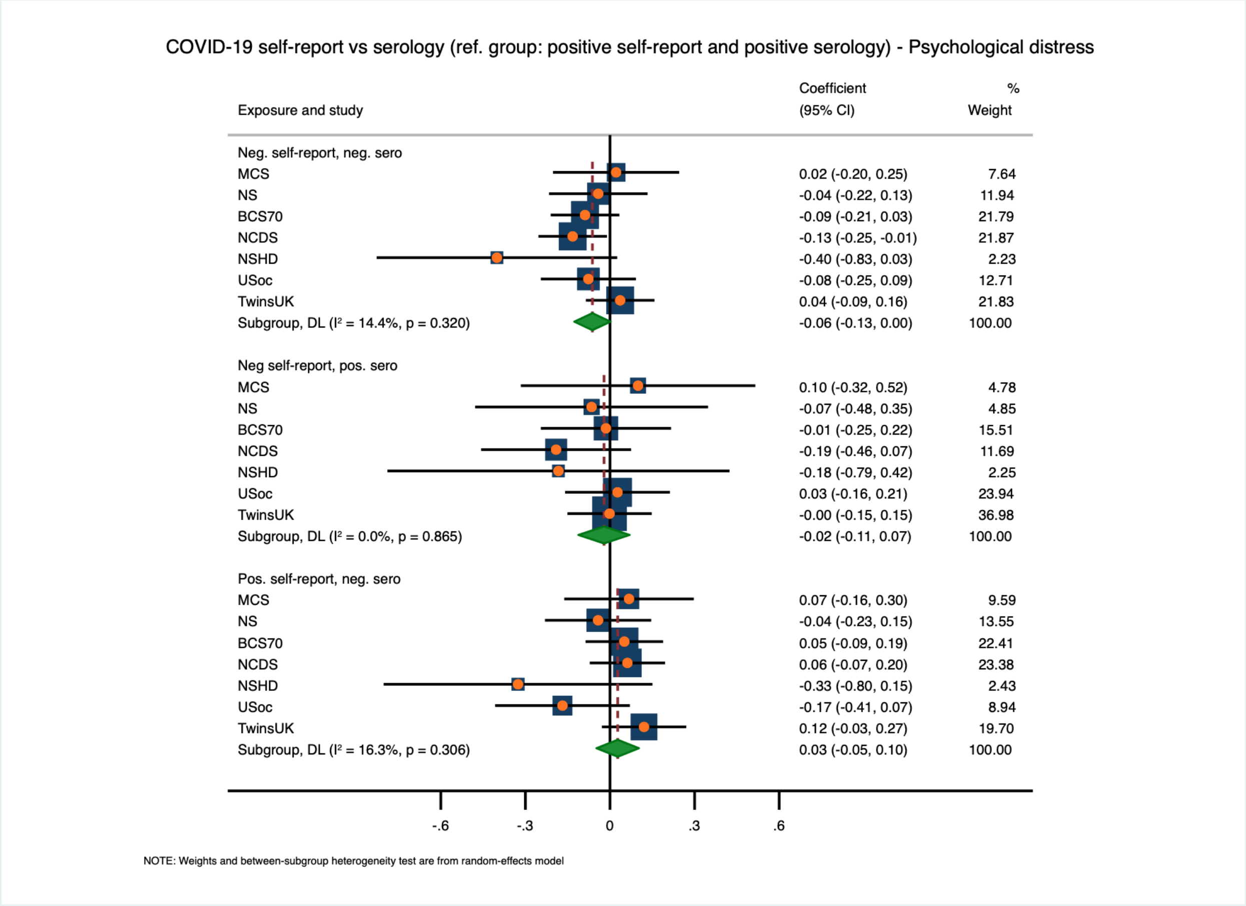

Figure S39. Self-report vs serology COVID-19 and depression (positive self-report and positive serology as reference group)

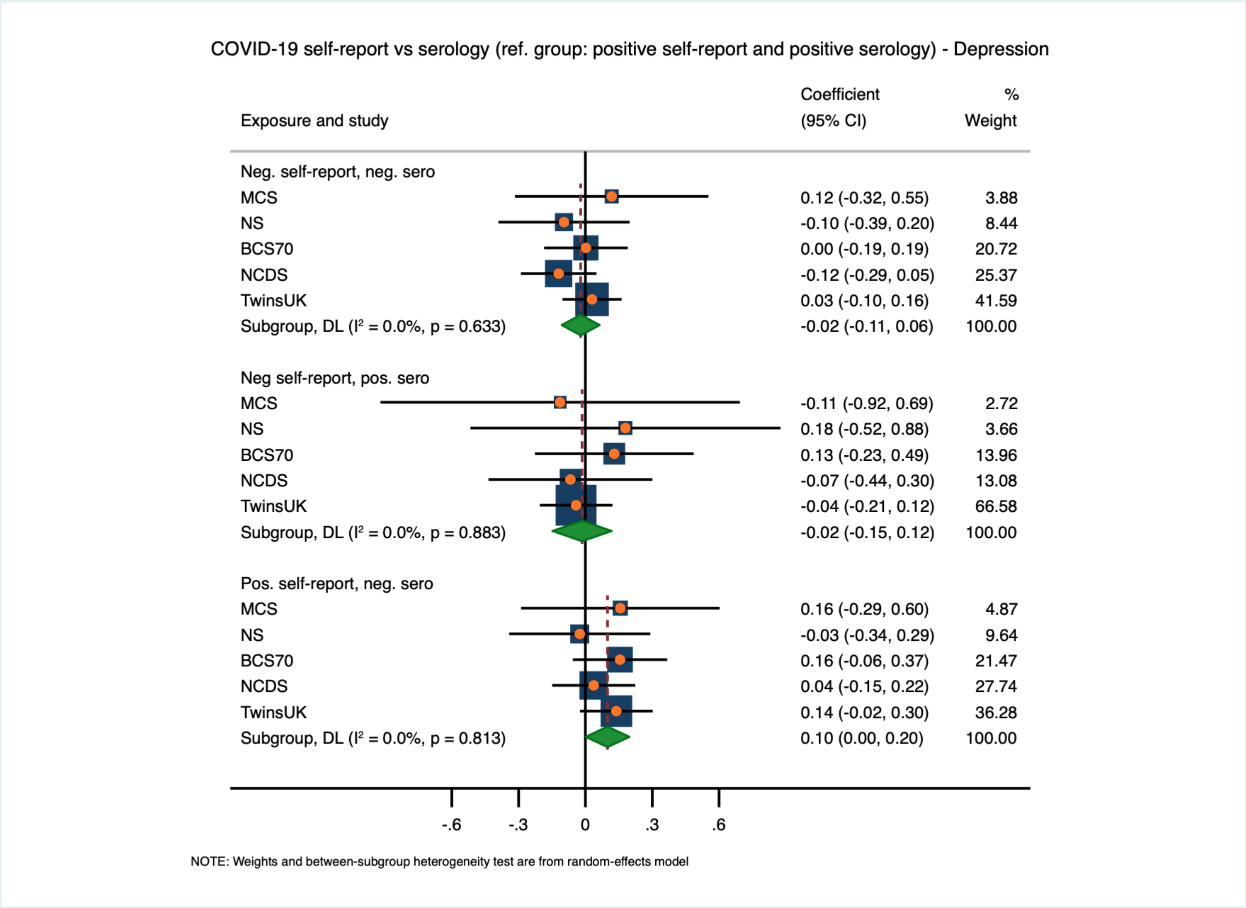

Figure S40. Self-report vs serology COVID-19 and anxiety (positive self-report and positive serology as reference group)

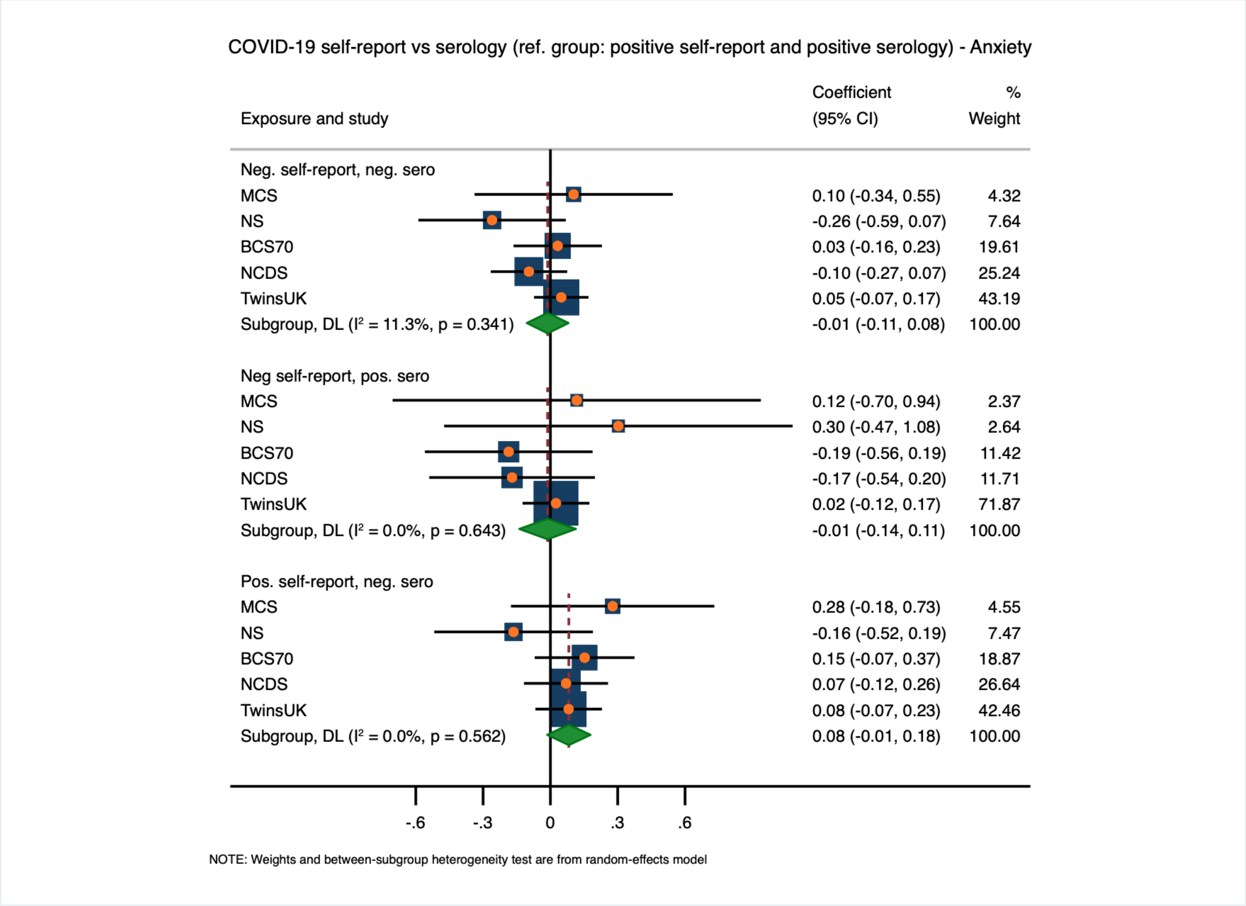

Figure S41. Self-report vs serology COVID-19 and life satisfaction (positive self-report and positive serology as reference group)

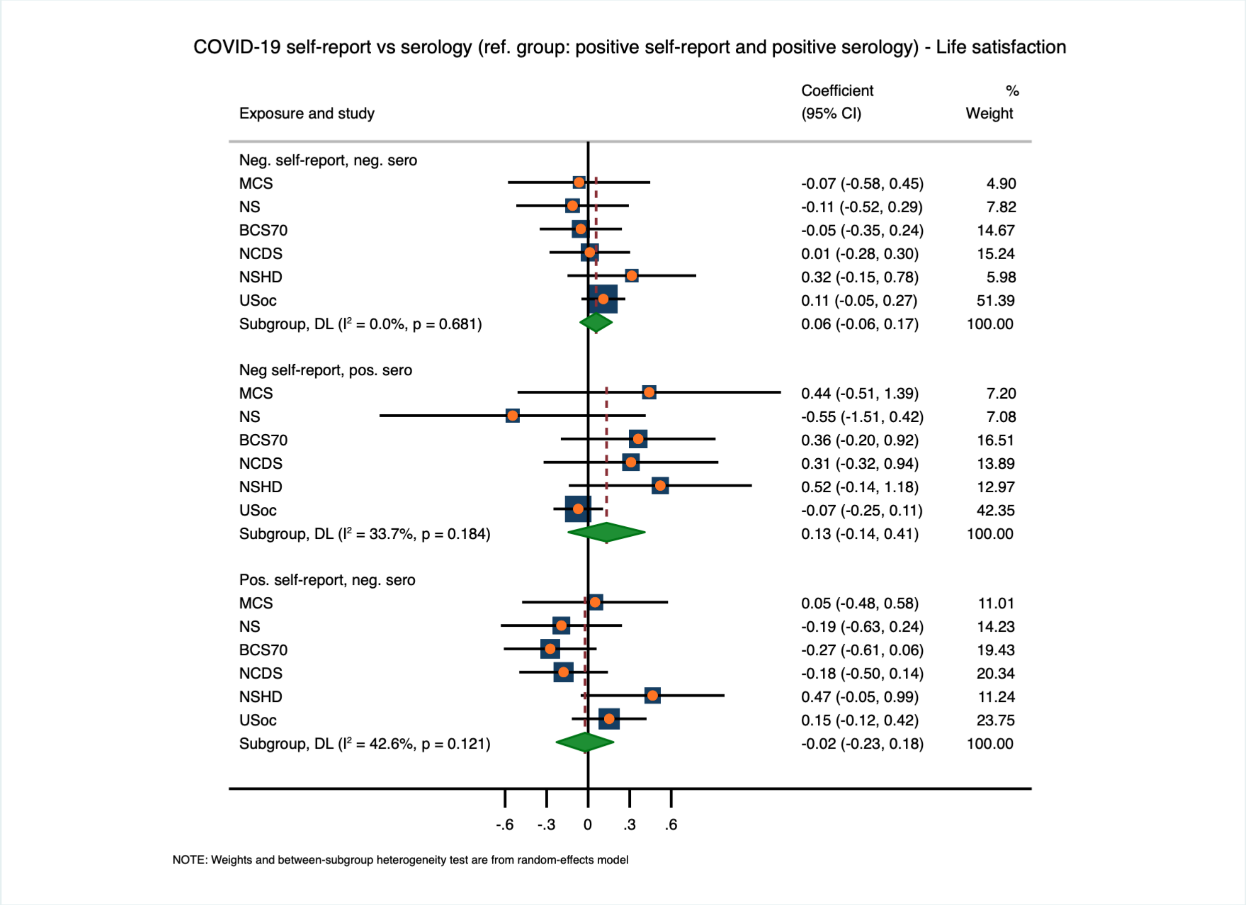

Supplementary financial information

ALSPAC: The UK Medical Research Council and Wellcome (Grant Ref: 217065/Z/19/Z) and the University of Bristol provide core support for ALSPAC. A comprehensive list of grants funding is available on the ALSPAC website (http://www.bristol.ac.uk/alspac/external/documents/grant-acknowledgements.pdf). We are extremely grateful to all the families who took part in this study, the midwives for their help in recruiting them, and the whole ALSPAC team, which includes interviewers, computer and laboratory technicians, clerical workers, research scientists, volunteers, managers, receptionists and nurses. This work was supported by Wellcome through the Wellcome Longitudinal Population Studies COVID-19 Secretariat and Steering Group (UK LPS COVID co-ordination, Grant Ref: 221574/Z/20/Z) and supported by the Elizabeth Blackwell Institute, University of Bristol, Wellcome Trust Institutional Strategic Support Fund and Rosetrees Trust (Grant Ref: 204813/Z/16/Z; R105121). ASFK is funded by an Economics and Social Research Council (ESRC) Postdoctoral Fellowship (ES/V011650/1).

USOC: Understanding Society is an initiative funded by the Economic and Social Research Council and various Government Departments, with scientific leadership by the Institute for Social and Economic Research, University of Essex, and survey delivery by NatCen Social Research and Kantar Public. The Understanding Society COVID-19 study is funded by the Economic and Social Research Council (ES/K005146/1) and the Health Foundation (2076161). The research data are distributed by the UK Data Service.

MCS, NS, BCS, NCDS, NSHD: The Millennium Cohort Study, Next Steps, 1970 British Cohort Study and 1958 National Child Development Study are supported by the Centre for Longitudinal Studies, Resource Centre 2015-20 grant (ES/M001660/1) and a host of other co-funders. The 1946 NSHD cohort is hosted by the the MRC Unit for Lifelong Health and Ageing funded by the Medical Research Council (MC_UU_00019/1Theme 1: Cohorts and Data Collection). The COVID-19 data collections in these five cohorts were funded by the UKRI grant Understanding the economic, social and health impacts of COVID-19 using lifetime data: evidence from 5 nationally representative UK cohorts (ES/V012789/1)

ELSA: The English Longitudinal Study of Ageing was developed by a team of researchers based at University College London, NatCen Social Research, the Institute for Fiscal Studies, the University of Manchester and the University of East Anglia. The data were collected by NatCen Social Research. The funding is currently provided by the National Institute on Aging in the US, and a consortium of UK government departments coordinated by the National Institute for Health Research. Funding has also been received by the Economic and Social Research Council. The English Longitudinal Study of Ageing Covid-19 Substudy was supported by the UK Economic and Social Research Grant (ESRC) ES/V003941/1.

GS: Generation Scotland received core support from the Chief Scientist Office of the Scottish Government Health Directorates [CZD/16/6] and the Scottish Funding Council [HR03006]. Genotyping of the GS:SFHS samples was carried out by the Genetics Core Laboratory at the Wellcome Trust Clinical Research Facility, Edinburgh, Scotland and was funded by the Medical Research Council UK and the Wellcome Trust (Wellcome Trust Strategic Award "STratifying Resilience and Depression Longitudinally" (STRADL) Reference 104036/Z/14/Z). Generation Scotland is funded by the Wellcome Trust (216767/Z/19/Z).

TwinsUK: TwinsUK receives funding from the Wellcome Trust (WT212904/Z/18/Z), the National Institute for Health Research (NIHR) Biomedical Research Centre based at Guy's and St Thomas' NHS Foundation Trust and King's College London. The TwinsUK COVID-19 personal experience study was funded by the King's Together Rapid COVID-19 Call award, under the projects original title 'Keeping together through coronavirus: The physical and mental health implications of self-isolation due to the Covid-19 TwinsUK is also supported by the Chronic Disease Research Foundation and Zoe Global Ltd. The funders had no role in study design, data collection and analysis, decision to publish, or preparation of the manuscript.

Additional Funding and acknowledgements

NJT is a Wellcome Trust Investigator (202802/Z/16/Z), is the PI of the Avon Longitudinal Study of Parents and Children (MRC & WT 217065/Z/19/Z), is supported by the University of Bristol NIHR Biomedical Research Centre, the MRC Integrative Epidemiology Unit (MC_UU_00011/1) and works within the CRUK Integrative Cancer Epidemiology Programme (C18281/A29019). SVK acknowledges funding from a NRS Senior Clinical Fellowship (SCAF/15/02), the Medical Research Council (MC_UU_00022/2) and the Scottish Government Chief Scientist Office (SPHSU17). ASFK acknowledges funding from the ESRC (ES/V011650/1). EJT acknowledges funding from the Wellcome Trust (WT212904/Z/18/Z). GBP acknowledges funding from the Economic and Social Research Council (ES/V012789/1). KT works in a Unit that is supported by the University of Bristol and UK Medical Research Council (MC_UU_00011/3). NC is supported by funding from the UK Medical Research Council (MC_UU_00019/2). We would also like to achnowledge the following individuals: Generation Scotland: Drew Altschul, Chloe Fawns-Ritchie, Archie Campbell, Robin Flaig; ALSPAC: Daniel J Smith; Understanding Society: Michaela Benzeval; TwinsUK: Deborah Hart, María Paz García, Nathan Cheetham, Katie J. Doores; Centre for Longitudinal Studies: Matt Brown, Lisa Calderwood, Emla Fitzsimons, Alissa Goodman, Aida Sanchez; Born in Bradford: John Wright, Dan Mason

Search terms for PubMed

Mental health
Psychological distress or mental health or mental illness or mental disorder or psychiatric symptom* or psychiatric disorder* or psychiatric illness* or psychiatric diagnos* or psychiatric condition* or mental disorder* or depression or depressive or depressed or affective or mood disorder* or anxiety or anxious
**COVID-19**
Covid-19 or coronavirus disease 2019 or SARS-CoV-2 or severe acute respiratory syndrome coronavirus 2

STROBE Statement—Checklist of items that should be included in reports of *cohort studies*

	Item No	Recommendation	Page No
**Title and abstract**	1	(*a*) Indicate the study's design with a commonly used term in the title or the abstract	1
		(*b*) Provide in the abstract an informative and balanced summary of what was done and what was found	2
**Introduction**			
Background/rationale	2	Explain the scientific background and rationale for the investigation being reported	4
Objectives	3	State specific objectives, including any prespecified hypotheses	4
**Methods**			
Study design	4	Present key elements of study design early in the paper	4-5
Setting	5	Describe the setting, locations, and relevant dates, including periods of recruitment, exposure, follow-up, and data collection	5
Participants	6	(*a*) Give the eligibility criteria, and the sources and methods of selection of participants. Describe methods of follow-up	5
		(*b*) For matched studies, give matching criteria and number of exposed and unexposed	
Variables	7	Clearly define all outcomes, exposures, predictors, potential confounders, and effect modifiers. Give diagnostic criteria, if applicable	5-7
Data sources/ measurement	8*	For each variable of interest, give sources of data and details of methods of assessment (measurement). Describe comparability of assessment methods if there is more than one group	5-7; supplement
Bias	9	Describe any efforts to address potential sources of bias	6-7

Study size	10	Explain how the study size was arrived at	5
Quantitative variables	11	Explain how quantitative variables were handled in the analyses. If applicable, describe which groupings were chosen and why	5-6
Statistical methods	12	(*a*) Describe all statistical methods, including those used to control for confounding	6-7
		(*b*) Describe any methods used to examine subgroups and interactions	6-7
		(*c*) Explain how missing data were addressed	5
		(*d*) If applicable, explain how loss to follow-up was addressed	
		(*e*) Describe any sensitivity analyses	7
**Results**			
Participants	13*	(a) Report numbers of individuals at each stage of study—eg numbers potentially eligible, examined for eligibility, confirmed eligible, included in the study, completing follow-up, and analysed	9; Table 1; supplement
		(b) Give reasons for non-participation at each stage	Supplement
		(c) Consider use of a flow diagram	
Descriptive data	14*	(a) Give characteristics of study participants (eg demographic, clinical, social) and information on exposures and potential confounders	8; supplement
		(b) Indicate number of participants with missing data for each variable of interest	Table 1; supplement
		(c) Summarise follow-up time (eg, average and total amount)	8
Outcome data	15*	Report numbers of outcome events or summary measures over time	8; supplement

Main results	16	(*a*) Give unadjusted estimates and, if applicable, confounder-adjusted estimates and their precision (eg, 95% confidence interval). Make clear which confounders were adjusted for and why they were included	8-9
		(*b*) Report category boundaries when continuous variables were categorized	8-9
		(*c*) If relevant, consider translating estimates of relative risk into absolute risk for a meaningful time period	
Other analyses	17	Report other analyses done—eg analyses of subgroups and interactions, and sensitivity analyses	8-9
**Discussion**			
Key results	18	Summarise key results with reference to study objectives	9-10
Limitations	19	Discuss limitations of the study, taking into account sources of potential bias or imprecision. Discuss both direction and magnitude of any potential bias	10-11
Interpretation	20	Give a cautious overall interpretation of results considering objectives, limitations, multiplicity of analyses, results from similar studies, and other relevant evidence	10-11
Generalisability	21	Discuss the generalisability (external validity) of the study results	11
**Other information**			
Funding	22	Give the source of funding and the role of the funders for the present study and, if applicable, for the original study on which the present article is based	7; 12 supplement

*Give information separately for exposed and unexposed groups.

Note: An Explanation and Elaboration article discusses each checklist item and gives methodological background and published examples of transparent reporting. The STROBE checklist is best used in conjunction with this article (freely available on the Web sites of PLoS Medicine at http://www.plosmedicine.org/, Annals of Internal Medicine at http://www.annals.org/, and Epidemiology at http://www.epidem.com/). Information on the STROBE Initiative is available at http://www.strobe-statement.org.
